# The relationship between immune and cognitive dysfunction in mood and psychotic disorder: a systematic review and a meta-analysis

**DOI:** 10.1038/s41380-022-01582-y

**Published:** 2022-04-28

**Authors:** M. Morrens, C. Overloop, V. Coppens, E. Loots, M. Van Den Noortgate, S. Vandenameele, M. Leboyer, L. De Picker

**Affiliations:** 1 Faculty of Medicine and Health Sciences, Collaborative Antwerp Psychiatric Research Institute (CAPRI), University of Antwerp, Antwerp, Belgium; 2 Scientific Initiative of Neuropsychiatric and Psychopharmacological Studies (SINAPS), University Psychiatric Centre Duffel, Duffel, Belgium; 3 Faculty of Medicine and Health Sciences, Nursing and obstetrics, University of Antwerp, Antwerp, Belgium; 4 University Hospital Brussels, Brussels Health Campus, Jette, Belgium; 5 INSERM U955, Equipe Psychiatrie Translationnelle, Créteil, France; 6 Fondation FondaMental, Créteil, France; 7 AP-HP, Hôpitaux Universitaires Henri Mondor, DHU Pepsy, Pôle de Psychiatrie et d’Addictologie, Créteil, France; 8 Université Paris Est Créteil, Faculté de Médecine, Creteil, France

**Keywords:** Depression, Schizophrenia, Bipolar disorder, Diagnostic markers

## Abstract

**Background:**

In psychotic and mood disorders, immune alterations are hypothesized to underlie cognitive symptoms, as they have been associated with elevated blood levels of inflammatory cytokines, kynurenine metabolites, and markers of microglial activation. The current meta-analysis synthesizes all available clinical evidence on the associations between immunomarkers (IMs) and cognition in these psychiatric illnesses.

**Methods:**

Pubmed, Web of Science, and Psycinfo were searched for peer-reviewed studies on schizophrenia spectrum disorder (SZ), bipolar disorder (BD), or major depressive disorder (MDD) including an association analysis between at least one baseline neuropsychological outcome measure (NP) and one IM (PROSPERO ID:CRD42021278371). Quality assessment was performed using BIOCROSS. Correlation meta-analyses, and random effect models, were conducted in Comprehensive Meta-Analysis version 3 investigating the association between eight cognitive domains and pro-inflammatory and anti-inflammatory indices (PII and AII) as well as individual IM.

**Results:**

Seventy-five studies (*n* = 29,104) revealed global cognitive performance (GCP) to be very weakly associated to PII (*r* = −0.076; *p* = 0.003; *I*^2^ = 77.4) or AII (*r* = 0.067; *p* = 0.334; *I*^2^ = 38.0) in the combined patient sample. Very weak associations between blood–based immune markers and global or domain-specific GCP were found, either combined or stratified by diagnostic subgroup (GCP x PII: SZ: *r* = −0.036, *p* = 0.370, *I*^2^ = 70.4; BD: *r* = −0.095, *p* = 0.013, *I*^2^ = 44.0; MDD: *r* = −0.133, *p* = 0.040, *I*^2^ = 83.5). We found evidence of publication bias.

**Discussion:**

There is evidence of only a weak association between blood-based immune markers and cognition in mood and psychotic disorders. Significant publication and reporting biases were observed and most likely underlie the inflation of such associations in individual studies.

## Introduction

Cognitive deficits are core features of severe mental disorders, and include memory, reasoning, attention, and information processing problems [1–4]. These deficits have been shown to be predictive of clinical and functional outcome, both cross-sectionally and longitudinally, in psychotic [5, 6] as well as mood disorders [7–9]. Regrettably, traditional treatment options such as antipsychotics [10, 11], mood stabilizers [12, 13], or antidepressants [14, 15] have limited or no beneficial effects on these cognitive symptoms. Besides monoaminergic signaling [16–18], other neurotransmitters including the GABAergic [19], and nicotinergic [20] systems but also hormonal changes [13, 21] and altered neuroplasticity [22] have been connected with cognitive dysfunctioning. Several potential cognitive enhancers targeting these mechanisms have been investigated with limited success [12], in turn stimulating the search for new treatment targets.

Disruption of the immune system is an important feature of psychotic and mood disorders [23–25] and is characterized by central immune changes such as altered microglial activity [26–28] as well as peripheral changes in cytokine levels [23], alterations in the kynurenine metabolism [29, 30] and white blood cell ratios [31]. While modest beneficial effects of adjunctive therapy with anti-inflammatory agents have been demonstrated for depressive, negative (i.e., apathy, flattened effect, poverty of thought or speech) and psychotic symptoms of severe mental disorders [32–34], it is unclear if this is also the case for cognitive symptomatology [35, 36]. Cognitive dysfunction in psychotic and mood disorders has been associated with elevated blood levels of inflammatory cytokines [37–39], kynurenine metabolites [40], and markers of microglial activation [40] in observational studies. However, these associations are typically modest and inconsistent in nature and seem to be subject to reporting bias with several studies only highlighting significant correlations between immune and cognitive markers while leaving other non-significant associations unreported [41–45].

The aim of the current meta-analysis is to synthesize all available evidence on the associations between immunomarkers and cognitive symptomatology in clinical observational studies of patients with psychotic and mood disorders.

## Materials and methods

We performed a systematic review and meta-analysis of studies reporting on the association between one or more immunomarkers and cognitive functioning in people with psychotic or mood disorders (PROSPERO ID:CRD42021278371, for updates to protocol, see Supplement). The study was conducted according to the Preferred Reporting Items for Systematic Reviews and Meta-analysis PRISMA 2020 standard (Supplementary Table 1) [46].

### Search strategy

A multistep procedure was used to conduct the literature search and consequent data assessment. The search included original papers published up to 8 November 2021 on Pubmed, Web of Science, and Psycinfo. The full search strings are available in the Supplement. Two authors independently (MM, CO) performed the literature search and completed the screening of article titles and abstracts for eligibility. In case of disagreement, papers were retained for full-text evaluation.

Inclusion criteria were: (1) Cross-sectional or longitudinal studies in peer-reviewed journals, (2) In vivo human studies on patients with primary diagnosis of schizophrenia (DSM-code: 295.90; ICD-10 code: F20.9), schizophreniform (295.40; F20.81), schizoaffective disorder (295.70; F25.2), brief psychotic disorder (298.8; F23), other psychotic disorder (298.9, F29), major depressive disorder (MDD) (296.20–296.36; F32.0-F33.3), persistent depressive disorder (dysthymia) (300.4; F34.1), BD (296.4x, 296.89, F31); (3) measurement of at least one baseline a) standardized neuropsychological outcome measure (NP) and b) immunomarker (IM) in the patient group, and (4) Studies included and reported an association analysis between at least one NP and IM. Exclusion criteria were: Case reports, case series; abstracts, conference proceedings; systematic or narrative reviews; opinion papers; meta-analyses; preprints. No language or time limits were used. Cognitive items on rating scales (e.g., the Positive and Negative Syndrome Scale) were not included as NP. Studies reporting on primary diagnoses of either schizophrenia, schizophreniform, schizoaffective disorder, psychotic disorder not otherwise specified (NOS), or brief psychotic disorder are hereafter combinatorially referred to as schizophrenia spectrum disorder and abbreviated as “SZ”.

### Data extraction and evaluation

For each included study, the following meta-analytic data were extracted (MM, CO): publication year, clinical diagnosis, mean age, gender (male participant ratio), mean duration of illness, mean body-mass index (BMI), smoking (ratio) as well as correlation values (Pearson's *r* or rho value), regression coefficients (beta score) on the association between IM and NP. The number of potentially reportable association measures (n IM × n NP), as well as the number of actually reported association measures, were equally extracted. Authors were contacted by email for additional information if all required data could not be retrieved from the paper; a reminder was sent in case of non-response. Beta coefficients were converted to r values according to the method described by Peterson and Brown [47]. For longitudinal studies, only baseline results were taken into consideration.

The primary NP outcome variable was global cognitive performance (GCP), defined as either (a) the composite score of cognitive functioning as reported by the study or (b) if no composite score was reported a composite correlation coefficient was calculated using the IM × NP associations in two or more separate cognitive domains (based on Fisher’s *Z* transformation). Higher GCP scores represent better cognitive performance.

Secondary domain-specific NP outcome variables were assigned to one of seven cognitive domains in accordance with the NIMH MATRICS initiative (“Measurement and Treatment Research to Improve Cognition in Schizophrenia”): Verbal learning and memory, Visual learning and memory, Working memory, attention and vigilance, Processing Speed, Reasoning and problem-solving [48]. “Language” was added as a separate category (see Supplementary Table 3).

The primary IM outcome variables were two composite inflammatory indices (Pro-inflammatory Index, PII); Anti-Inflammatory Index, AII). If only a single IM was included in the study, that IM×NP correlation coefficient was used for the PII/AII associations. The following immune markers were categorized as PII: (hs)CRP, IL-1b, IL-2, IL-3, IL-6, IL-7, IL-8, IL-12-p70, IL-15, IL-16, IL-17, IL-18, IL-33, IFN-g, TNF-a, TNF-b, sTNFR1, sTNFR2, CCL-11, CCL-17, CCL-22, CXCL-10, MCP-1, sST2. AII were: IL-1ra, IL-4, IL-10, IL-11, IL-13, TGF-b (see Supplementary Table 4). If more IM were included, PII and AII were calculated for each cognitive domain, averaging out the associations between that specific cognitive domain on one hand and the available immunomarker-based correlations on the other (based on Fisher’s z transformation of the *r* values [49]). This resulted in a single r score reflecting the association of the merged correlational values between the cognitive domain and the pro-inflammatory and anti-inflammatory markers. This method of combining multiple pro-inflammatory markers in a single composite inflammatory score is in line with previous similar efforts in psychosocial stress [50], depression [51], schizophrenia [52], psychological trauma [53], cerebrovascular disease [54], atherosclerosis [55], aging [56] and carcinoma [57] research. Secondary IM outcome variables were individual IM when more than three studies were available. In case of a significant association, exploratory subgroup analyses were performed to define which of the diagnoses contributed to the statistically significant association.

Quality assessment of eligible papers was performed with the BIOCROSS evaluation tool [58], which is specifically developed for biomarker-based cross-sectional studies. Quality assessment of each included study was performed independently by two different authors (MM, CO, EL), and any disagreement was resolved by deliberation.

### Data synthesis and analysis

 The correlation meta-analyses were conducted in Comprehensive Meta-Analysis version 3 (CMA v3) using random-effect models, which use the Hedges-Olkin method with a Fisher Z transformation of the correlation coefficient [59]. Heterogeneity was estimated with *I*^2^ (heterogeneity classification: *I*^2^ = 25–49%: low; *I*^2^ = 50–74%: moderate *I*^2^ ≥ 75%: high). Because the *p* value of correlation analysis is known to be strongly influenced by the sample size of the analysis, we opted to evaluate the association based on the strength of the association (using the *r* value) rather than the *p* value. Following Evans [60], meta-analytic correlation effect estimates of |0–0.19| were considered to be “very weak”, |0.20–0.39| as “weak”, |0.40–0.59| “moderate”, |0.60–0.79| as “strong” and | 0.80| or above as “very strong”.

The primary meta-analysis was performed on the effects of pro-inflammatory and anti-inflammatory markers (see Supplementary Table 4) in all mood and psychotic disorders combined for global cognitive function. Secondary analysis was performed for each cognitive domain, and for individual immunomarkers if at least three studies were available. Subgroup analysis was performed to evaluate differences between diagnostic groups primary and secondary analyses. The Benjamini–Hochberg procedure was used to account for the False Discovery Rate (FDR) in order to control for multiple hypothesis testing.

Additional meta-regression analyses (Knapp–Hartung method, maximum likelihood) [61] were conducted to evaluate the effect of the following moderators in the primary analysis: publication year, mean age, male participant percentage, mean duration of illness, mean BMI, smoking ratio and sample size. Publication bias was assessed by visual inspection of the funnel plots and Egger’s regression test [62].

The primary meta-analysis was repeated including only high-quality studies as defined by the BIOCROSS quality assessments.

## Results

### Study selection

The results of the literature search are summarized in the PRISMA Flowchart. Additional data were requested for 88, and granted for 22 papers (response rate 25%). A total of 75 studies [24,41–45,63–130] and 627 NP × IM associations were included in the meta-analysis (see Supplementary Figure 1 for PRISMA Flowchart). Forty-two studies focused on SZ, 17 studies investigated BD, and 18 studies included MDD patients, for a total sample of 29,104 patients (see also Supplementary Tables 5–7). Thirty-one studies reported cognitive associations with a single IM, whereas 44 studies included multiple IM (see Supplementary Table 6).

The following IM were included in the meta-analysis: C-reactive protein ((hs-)CRP), cytokines/chemokines (CCL-11; CCL-17; CCL-22; CXCL-10; IFN-g; IL-1; IL-1b; IL-2; IL-3; IL-4; IL-6; IL-7; IL-10; IL-12; IL-12p70; IL-15; IL-16; IL-17; IL-18; IL-33; MCP-1; sST2; sTNFR-R1; sTNFR2; TGF-b; TNF-a) or kynurenine metabolites (Tryptophan (TRP); Kynurenine (KYN); Kynurenic Acid (KA); 3-hydroxy-kynurenine (3-HK); Quinolinic Acid (QUIN)). A single study [130] looked into the association of cognition with immunomarker levels in CSF while all other studies focused on peripheral assessments (serum/plasma). Therefore, CSF data were not included in the current meta-analysis. No studies investigated associations between NP and leukocyte IM.

PII correlation scores were calculated for a total of 18 out of 39 studies in the verbal memory domain, 6 out of 14 studies in visual memory, working memory (14 out of 15 studies); attention (12 out of 35 studies); processing speed (12 out of 23 studies); reasoning (18 out of 42 studies); language (9 out of 22 studies) (see Supplementary Table 7).

### Association of pro-inflammatory (PII) and anti-inflammatory index (AII) with global cognitive performance (GCP)

PII and AII × GCP interactions were available for 53 studies (*n* = 27.908). Over the three eligible diagnostic groups (MDD, SZ, BD), GCP was very weakly associated with PII (PII × GCP *r* = −0.076; 95% CI = −0.116 to −0.027, *z* = −3.011; *p* = 0.003, *I*² = 77.4; see Table 1; Supplementary Figures 2–9), an association that can be considered to be neglectable.

**Table 1:** Effect sizes for the relationship between pro-inflammatory markers and cognitive dysfunctioning.

Test	*k*	*N*	*r*	95% CI	*z*	*p*	*Q* test	*P* value (*Q* test)	*I*^2^
*All psychotic and mood disorders*									
Global cognitive performance	53	27,908	−0.076	−0.126 to −0.027	−3.011	0.003*	230.141	<0.001	77.4
Verbal memory	36	3501	−0.089	−0.148 to −0.029	−2.900	0.004*	128.63	<0.001	72.79
Visual memory	15	24,174	−0.036	−0.049 to −0.023	−5.533	<0.001*	29.17	0.010	52.01
Working memory	23	1902	−0.065	−0.118 to −0.011	−2.363	0.018*	37.95	0.019	42.03
Attention	27	26,243	−0.027	−0.091 to 0.037	−0.820	0.412	160.69	<0.001	83.82
Processing speed	20	2149	−0.082	−0.151 to −0.012	−2.295	0.022*	47.46	<0.001	59.96
Reasoning	37	3284	−0.025	−0.096 to 0.045	−0.882	0.378	80.18	<0.001	55.10
Language	21	2419	−0.052	−0.144 to 0.041	−1.100	0.271	85.56	<0.001	76.62
*Schizophrenia*									
Global cognitive performance	27	2609	−0.036	−0.113 to 0.042	−0.897	0.370	87.82	<0.001	70.39
Verbal memory	22	2156	−0.072	−0.172 to 0.030	−1.377	0.168	99.34	<0.001	78.86
Visual memory	9	745	−0.142	−0.241 to −0.040	−2.724	0.006*	12.93	0.114	38.13
Working memory	16	1267	0.002	−0.071 to 0.076	0.066	0.947	22.66	0.092	33.79
Attention	16	2011	−0.071	−0.164 to 0.022	−1.496	0.135	49.65	<0.001	69.79
Processing speed	12	1314	−0.080	−0.171 to 0.013	−1.695	0.090	25.11	0.009	56.18
Reasoning	18	1786	−0.026	−0.096 to 0.045	−0.712	0.476	31.52	0.017	46.06
Language	16	1861	−0.033	−0.137 to 0.072	−0.616	0.538	65.62	<0.001	77.14
Bipolar disorder									
Global cognitive performance	13	2012	−0.095	−0.168 to −0.020	−2.474	0.013*	21.42	0.045	43.98
Verbal memory	8	929	−0.085	−0.164 to −0.005	−2.082	0.037	9.52	0.218	26.45
Visual memory	4	839	−0.108	−0.196 to −0.019	−2.379	0.017*	4.017	0.260	25.31
Working memory	6	605	−0.153	−0.231 to −0.074	−3.744	<0.001*	4.65	0.460	0.00
Attention	5	1366	0.025	−0.105 to 0.154	0.371	0.711	11.61	0.021	65.54
Processing speed	5	591	−0.130	−0.318 to 0.067	−1.294	0.196	20.07	<0.001	80.07
Reasoning	12	1036	0.002	−0.124 to 0.128	0.033	0.974	37.42	<0.001	70.60
Language	3	458	−0.162	−0.363 to 0.053	−1.482	0.138	9.01	0.011	77.80
*MDD*									
Global cognitive performance	13	23,245	−0.133	−0.257 to −0.006	−2.051	0.040	72.71	<0.001	83.50
Verbal memory	6	416	−0.186	−0.375 to 0.018	−1.785	0.074	19.62	0.001	74.51
Attention	6	22,866	0.003	−0.117 to 0.123	0.043	0.965	15.25	0.009	67.22
Processing speed	3	244	−0.066	−0.191 to 0.062	−1.009	0.313	0.29	0.863	0.00
Reasoning	7	462	−0.053	−0.183 to 0.079	−0.786	0.432	9.95	0.127	39.67

*K* number of studies, *N* total number of participants, *r* mean effect size of correlation.

*Statistically significant after Benjamini–Hochberg procedure correction.

Subgroup analysis revealed no significant differences between the three diagnostic groups (Q(2)=2.302; p = 0.316), with very weak associations in each group for PII × GCP (SZ: *n* studies = 27, *r* = −0.036, *p* = 0.370, *I*² = 70.4; BD: *n* studies = 13, *r* = −0.095, *p* = 0.013, *I*² = 44.0; MDD n studies = 13, *r* = −0.133, *p* = 0.040 (not significant after FDR correction), *I*² = 83.5); see Table 1).

While only five studies yielded AII × GCP interactions, no significant associations were found (see Table 2; Supplementary Figures S10–S14).

**Table 2:** Effect sizes for the relationship between anti-inflammatory markers and cognitive dysfunctioning.

Test	*k*	*N*	*r*	95% CI	*z*	*p*	*Q* test	*Q* test (*p*)	*I*^2^
Global cognitive performance	5	408	0.067	−0.069 to 0.201	0.966	0.334	6.46	0.168	38.03
Verbal memory	3	264	−0.074	−0.240 to 0.097	−0.846	0.398	3.31	0.191	39.65
Processing speed	4	314	0.125	−0.030 to 0.275	1.587	0.113	5.41	0.144	44.58
Reasoning	4	249	−0.002	−0.245 to 0.241	−0.019	0.985	7.53	0.057	60.14

*K* number of studies, *N* total number of participants, *r* mean effect size of correlation.

### Association of PII and AII with domain-specific cognitive performances

Overall association measures for PII were smaller than |0.10| for all domain-specific cognitive outcome variables. In the diagnostic subgroups analysis, significant but very weak associations were observed for visual memory in SZ, and for visual and verbal memory, working memory, and language in BD (see Table 1). Notably, the analyses reporting a statistically significant association in the diagnostic subgroups typically contained a lower number of included studies and smaller total sample sizes than analyses reporting non-significant associations. Meta-regression analysis for PII × GCP associations in the total sample with sample size as covariate did not reveal a significant confounding effect (coefficient (SE) = 0.00 (0.00); *z* = 0.97; *p* = 0.330).

No significant associations were found between AII and specific domains such as verbal memory, processing speed, and reasoning (see Table 2).

The BIOCROSS evaluation tool [58] was used to assess the quality of the included studies (see Supplementary Table 8). Thirty-seven papers had high quality, 35 papers were rated as having moderate quality, two papers had low quality. When only studies of high quality were retained, the overall correlation between PII and the composite score became non-significant (*r* = −0.042; *z* = −1.955; *p* = 0.051). Similarly, when only considering high quality studies this correlation disappeared in all diagnostic subgroup (BD: *k* = 7, *r* = −0.032, *z* = −1.301, *p* = 0.193; MDD: *k* = 6, *r* = −0.120, *z* = −1.051, *p* = 0.293; SZ: *k* = 14, *r* = −0.064, *z* = −1.363, *p* = 0.173).

### Cognitive performance and individual IM

Only for the immunomarkers CRP, IL-1B, IL-6, TNF-a, and IFN-g, there were at least three studies available that assessed their association with cognitive performance in mood and psychotic disorders (See Table 3; Supplementary Figures S15–S19).

**Table 3:** Correlations between individual immunomarkers and cognition in mood and psychotic disorders.

Test	*k*	*N*	*r*	95% CI	*z*	*p*	*Q* test	*Q* test (*p*)	*I*^2^
*CRP*									
Global cognitive performance	22	25,948	−0.124	−0.185 to −0.062	−3.902	<0.001*	98.89	<0.001	79.78
Verbal memory	16	2304	−0.129	−0.203 to −0.053	−3.322	0.001*	45.03	<0.001	66.69
Visual memory	7	24,160	−0.108	−0.178 to −0.036	−2.960	0.003*	22.55	0.001	73.39
Working memory	10	1133	−0.123	−0.181 to −0.065	−4.109	<0.001*	7.97	0.538	0.00
Attention	14	227,274	−0.065	−0.142 to 0.012	−1.660	0.097	93.39	<0.001	86.08
Processing speed	11	1553	−0.056	−0.123 to 0.012	−1.620	0.105	16.13	0.096	38.01
Reasoning	14	1843	−0.082	−0.152 to −0.010	−2.239	0.025*	26.42	0.015	50.79
Language	8	1644	−0.151	−0.271 to −0.026	−2.364	0.018*	43.58	<0.001	83.94
*IL-1b*									
Global cognitive performance	10	988	−0.083	−0.170 to 0.006	−1.822	0.068	15.48	0.079	41.85
Verbal memory	6	731	−0.105	−0.311 to 0.111	−0.950	0.342	38.71	<0.001	87.08
Visual memory	3	256	−0.245	−0.542 to 0.108	−1.368	0.171	13.28	0.001	84.94
Working memory	5	569	0.092	−0.102 to 0.279	0.925	0.355	17.81	0.001	77.53
Attention	5	389	−0.014	−0.139 to 0.110	−0.225	0.822	5.59	0.232	28.46
Processing speed	7	724	−0.095	−0.253 to 0.068	−1.140	0.254	25.50	<0.001	76.47
Reasoning	6	350	−0.142	−0.298 to 0.022	−1.698	0.090	18.11	0.003	72.40
Language	4	499	−0.035	−0.290 to 0.224	−0.263	0.793	19.07	<0.001	84.27
*IL-6*									
Global cognitive performance	27	2250	−0.167	−0.259 to −0.072	−3.431	0.001*	119.99	<0.001	78.33
Verbal memory	18	1776	−0.131	−0.245 to −0.013	−2.181	0.029	96.98	<0.001	82.47
Visual memory	5	469	−0.016	−0.140 to 0.109	−0.247	0.805	6.32	0.176	36.72
Working memory	14	1200	−0.086	−0.192 to 0.022	−1.555	0.120	34.23	0.001	64.95
Attention	12	1013	−0.021	−0.128 to 0.085	−0.392	0.695	27.44	0.004	59.9
Processing speed	13	1266	−0.132	−0.211 to −0.051	−3.202	0.001*	18.12	0.079	39.28
Reasoning	16	1335	0.002	−0.089 to 0.093	0.037	0.971	35.06	0.002	57.22
Language	10	1179	−0.100	−0.213 to 0.014	−1.714	0.087	30.62	<0.001	70.60
*TNF-a*									
Global cognitive performance	23	1868	−0.028	−0.077 to 0.021	−1.119	0.263	23.75	0.360	7.37
Verbal memory	14	1280	−0.016	−0.108 to 0.077	−0.331	0.740	31.98	0.002	59.35
Visual memory	5	326	−0.081	−0.190 to 0.030	−1.437	0.151	3.23	0.520	0.00
Working memory	13	1017	−0.098	−0.197 to 0.002	−1.917	0.055	25.85	0.011	53.58
Attention	10	732	0.034	−0.103 to 0.169	0.482	0.630	26.89	0.001	66.53
Processing speed	14	1178	−0.020	−0.143 to 0.105	−0.307	0.759	45.54	<0.001	73.65
Reasoning	20	1619	0.019	−0.066 to 0.105	0.445	0.656	47.05	<0.001	59.62
Language	7	764	−0.004	−0.153 to 0.144	−0.057	0.955	20.92	0.002	71.32
*IFN-g*									
Global cognitive performance	5	203	0.055	−0.112 to 0.219	0.647	0.517	5.04	0.284	20.59
Verbal memory	4	187	0.060	−0.088 to 0.205	0.792	0.428	2.37	0.500	0.00
Working memory	3	127	0.035	−0.274 to 0.337	0.216	0.829	5.12	0.077	60.93
Attention	3	97	−0.144	−0.397 to 0.129	−1.033	0.302	2.65	0.266	24.39
Reasoning	4	158	0.062	−0.103 to 0.223	0.732	0.464	3.08	0.380	2.46

*K* number of studies, *N* total number of participants, *r* mean effect size of correlation.

*Statistically significant after Benjamini–Hochberg procedure correction.

CRP (22 studies; 25,948 patients) was significantly but very weakly associated with GCP (*r* = −0.124; *p* < 0.001, *I*² = 79.8), as well as with verbal and visual memory, working memory, reasoning, and language in the total patient cohort. Follow-up subgroup analyses revealed a significant but very weak association with global cognition to be reflected in schizophrenia (*r* = 0.139; *p* = 0.013) and bipolar disorder (*r* = 0.126; *p* = 0.016), but not in MDD. Associations remained significant after FDR correction. Significant and weak correlations were observed between CRP and attention, verbal memory and visual memory in SZ, and verbal memory, processing speed, reasoning, and language in BD.

IL-6 (27 studies; *n* = 2.250) was significantly but very weakly associated with global cognition (*r* = −0.167; *p* = 0.001), verbal memory, and processing speed but not with any of the other cognitive domains in the total patient cohort.

IL-1b (10 studies; *n* = 988), TNF-a (23 studies; *n* = 1868), and IFN-g (five studies; *n* = 203) were not associated with either the composite cognitive score or any of the separate cognitive domains.

Seven studies probed relations between several tryptophan catabolism (TRYCAT) metabolites (TRP, KYN KA, 3-HK, QUIN) and cognitive performance in mood and psychotic disorders. Again, none of the metabolites interacted significantly with cognition (see Table 4).

**Table 4:** Correlations between global cognitive performance and TRYCAT metabolites in mood and psychotic disorders.

Test	*k*	*N*	*r*	95% CI	*z*	*p*	*Q* test	*Q* test (p)	*I*^2^
TRP	4	243	0.047	−0.085 to 0.177	0.692	0.489	1.05	0.592	0.00
KYN	7	379	−0.030	−0.132 to 0.074	−0.560	0.575	2.45	0.875	0.00
KA	6	364	−0.127	−0.365 to 0.127	−0.977	0.329	24.63	<0.001	79.70
3-HK	3	129	0.064	−0.141 to 0.263	0.609	0.543	2.42	0.299	17.18
QA	4	205	−0.088	−0.237 to 0.066	−1.118	0.264	3.46	0.315	15.36

*K* number of studies, *N* total number of participants, *r* mean effect size of correlation.

### Covariate assessment

For the association between pro-inflammatory cytokines and global cognition, publication year (coefficient(SE) = −0.02(0.01); *z* = −0.38; *p* = 0.7035), mean patient age (coefficient(SE) = 0.01(0.01); *z* = 0.36; *p* = 0.720), mean Duration of Illness (coefficient(SE) = 0.00(0.02); *z* = −0.17; *p* = 0.862), BMI (coefficient(SE) = −0.01(0.04); *z* = −0.40; *p* = 0.689), gender ratio (coefficient(SE) = 0.00(0.01); *z* = 0.46; *p* = 0.642) and smoker/non-smoker ratio (coefficient(SE) = 0.01(0.00); *z* = 1.24; *p* = 0.217) all proved non-significant covariates in meta-regression analyses.

### Assessment of publication bias

Out of the included 75 papers, a total number of 627 out of the potential 1810 associations (number of cognitive measures × number of immunomarkers) were at disposition for the current meta-analysis (i.e., 35%). On average, 53% of the potential total number of associations were reported per study (MDD: 63%; SZ: 53%; BD: 43%).

Visual inspection of the funnel plots of the standard errors by Fisher’s Z scores for associations between GCP and PII, CRP and IL-6 respectively (see Supplementary Figures 20–22) indeed suggested the presence of a publication bias in favor of more pronounced negative correlations. The Egger regression test confirmed the potential presence of a publication bias (intercept = −1.30; *t* = 4.26; *p* < 0.0001). However, when conducted for each diagnosis independently, Egger’s test was only significant for MDD (intercept = −1.74; *t* = 2.68; *p* = 0.023), but not for SZ (intercept = 0.64; *t* = 0.68; *p* = 0.501) or bipolar disorder (intercept = −0.94; *t* = 1.37; *p* = 0.199).

Heterogeneity over studies was moderate to high (see Tables 1–4), especially for immunomarkers investigated in a larger number of studies. When evaluating individual biomarkers (see Table 4), heterogeneity tended to be higher for those associations with cognitive performance that yielded statistically significant correlations.

## Discussion

This is the first meta-analysis to synthesize the results from all available 75 studies investigating the association between cognitive functioning and blood-based immune marker levels in psychotic and mood disorders. Strengths of this study were the availability of a large total patient sample (*n* = 29,104), the use of a systematic multistep approach toward data assessment and meta-analysis in a transdiagnostic patient group, and the assessment of inflammatory composite scores as well as individual immunomarkers. We found no reliable evidence of a meaningful association between blood-based immune markers and global or domain-specific cognitive performance in mood and psychotic disorders (all *r*|<0.10|), either combined or stratified by diagnostic subgroup. Slightly larger but still weak associations were observed for individual IM (CRP, IL-6). Quality assessments revealed significant publication and reporting biases that may have contributed to the inflation of observed associations in individual studies.

This unexpected result warrants careful and thorough interpretation. The total sample size in our meta-analysis was sufficient to generate significant outcomes despite very weak association effect estimates, indicating they are not merely attributable to a lack of power. Findings are in line with a previous meta-analysis (including nine studies; *n* = 1477) demonstrating comparable associations between CRP and cognitive functioning (*r* = 0.13, *p* < 0.001) in schizophrenia [131]. We can therefore conclude that observations of blood-based immune markers are not or only (very) weakly related to neuropsychological performance scores in cross-sectional studies of patients with mood and cognitive disorders. However, the absence of an association between immune and cognitive markers does not necessarily imply that the immune system does not affect cognition in these disorders. Because of the dimensional and dynamic nature of both processes, other study designs, including longitudinal and intervention studies, may be required to evaluate the complex interaction between immune mechanisms and cognitive performance. Animals exposed to prenatal immune challenges demonstrate cognitive deficits comparable to those seen in mood and psychotic disorder patients [132]. Similarly, manipulation of brain kynurenic acid levels improves cognitive performance in mice [133, 134]. In humans, several clinical trials with immunomodulatory agents revealed mild cognitive improvements in schizophrenia patients [135, 136] although this was only the case for working memory and not any other cognitive domains, as demonstrated by Jeppesen et al. [137].

An alleged interaction between the altered immune system and cognitive functioning could still be present in the absence of cross-sectional intercorrelations. Two hypothetical mechanisms could be proposed: (1) Prolonged inflammatory stress may accelerate the normal cognitive decline over time through a number of indirect effects (oxidative stress, excitotoxicity, glial dysfunction, decreased synaptic plasticity, “inflammaging”), and/or (2) episodic excessive inflammatory reactions to psychological or environmental stressors could erode cognition step-by-step in a cascade-like fashion. As such, evaluating trait abnormalities like immune-related genetic markers or pre-illness immune abnormalities instead of volatile “snapshots” of individual compounds may be more informative to assess the relationship with cognitive functioning. Furthermore, while peripheral immunomarker levels fluctuate heavily over time [138], cognitive deficits are a more stable or slowly progressive phenomenon in mood and psychotic disorders [139]. As such, the temporal resolution of cross-sectional assessments of blood-based immunomarkers may not be sufficient to detect the long-term and/or long-ago repercussions of immunological causes on cognitive performance. Peripheral CRP and several cytokines (including IL-6 and TNF-a) have been demonstrated to be highly state-dependent in mood and psychotic illness [23, 24,140–143], suggesting they are more relevant as biomarkers for episodic symptoms like psychosis or mania. Alternatively, longitudinal characterization of immunomarkers and cognitive outcomes over longer time periods may be more informative than single cross-sectional assessments.

Another consideration is that peripheral blood concentrations may not reflect latent or undetected central immune-related processes [144, 145] that do interfere with cognitive performance. Undoubtedly, the lack of studies focusing on central assessments of immune activity in major psychiatric disorders is hampering our ability to determine the interplay between the immune system and cognitive function. The few available studies of central inflammatory responses are mostly small-scale and cross-sectional in nature, and typically do not include neuropsychological assessments [26, 27]. Even so, the most investigated of these molecules (CRP, IL-6, IL-1b) are potent but nonspecific immune markers that are produced by a variety of cells and have a myriad of pleiotropic effects in the brain [146, 147], rendering the interpretation of their potential impact on cognitive functioning difficult. It has been proposed that kynurenine metabolites are more closely related to cognitive functioning, due to their interaction with the glutamatergic and nicotinergic system [25] and while the current meta-analysis did not reveal an association with cognitive functioning, the number of studies focusing on these immune markers was very limited, and studies with larger sample sizes are needed. Finally, a few studies demonstrated increases of anti-inflammatory cytokines in processes associated with low-grade systemic inflammation [148, 149]. As a result, it has been questioned to what extent these changes that may be comparable to those seen in psychiatric illness actually represent anti-inflammatory properties in such conditions.

Several limitations should be acknowledged. Meta-analysis of correlation measures remains methodologically challenging, and this is particularly true for associations between two complex dimensions without standardized outcome measures, as is the case for immunological assays and neuropsychological testing in psychiatric disorders. The method used by meta-analysis software CMA tends to overestimate pooled effects [150], especially when correlation coefficients are higher. However, these coefficients tended to be low in the current series of meta-analyses, and even when present would only confirm the conclusions drawn in the current review. Moreover, although efforts were made to mitigate account for publication and reporting biases, they were demonstrably present and will have fundamentally impacted the results of the current meta-analysis. Several studies had small to very small sample sizes and were of moderate quality, which seems to have impacted the results, as analyses only including high-quality papers revealed even more modest associations between immune markers and cognition. The moderate to high overall heterogeneity might be attributable to several meta-analyses of inherent and individual study design-related factors. First, the calculated pro- and anti-inflammatory indexes amalgamate individual inflammatory compounds into two potentially arbitrary categories, at the risk of oversimplification or effect diffusion. Another limitation to be kept in mind is that basal blood levels of immunomarkers do not necessarily reflect the in vivo reactivity upon immune challenge. It should also be noted that both cognitive and immune assessments have methodological limitations contributing to measurement errors and other forms of noise that may mask the detection of an actual association. Finally, while we did not find a significant influence of publication year, age, gender, and duration of illness as covariates, other sources of confounding such as medication status or psychiatric symptom severity were not well accounted for in the included studies [151].

In conclusion, we found evidence of only a weak association between blood-based immune markers and cognition in mood and psychotic disorders. Although assuming an interaction between immune changes and cognitive symptomatology is appealing, evidence to convincingly support such a relationship in severe mental disorders is weak. Significant publication and reporting biases were observed and most likely underlie the inflation of such associations in individual studies. Efforts including central measures of immune activity, trait markers, longitudinal data, and immune challenges might prove more fruitful to uncover a hypothetical relationship between immune alterations and cognitive functioning in mood and psychotic disorders. Potentially, an extant relationship between these parameters can merely not be unveiled by the currently available methodologies and requires assessment techniques with higher resolution.

## Supplementary information

Supplementary Materials

## References

[1] Bora E, Pantelis C (2015). Meta-analysis of cognitive impairment in first-episode bipolar disorder: comparison with first-episode schizophrenia and healthy controls. Schizophr Bull.

[2] Bora E, Yucel M, Pantelis C (2009). Cognitive functioning in schizophrenia, schizoaffective disorder and affective psychoses: meta-analytic study. Br J Psychiatry.

[3] Rock PL, Roiser JP, Riedel WJ, Blackwell AD (2014). Cognitive impairment in depression: a systematic review and meta-analysis. Psychol Med.

[4] Morrens M, Hulstijn W, Matton C, Madani Y, van Bouwel L, Peuskens J (2008). Delineating psychomotor slowing from reduced processing speed in schizophrenia. Cogn Neuropsychiatry.

[5] Cowman M, Holleran L, Lonergan E, O’Connor K, Birchwood M, Donohoe G (2021). Cognitive predictors of social and occupational functioning in early psychosis: a systematic review and meta-analysis of cross-sectional and longitudinal data. Schizophr Bull.

[6] Fett A-KJ, Viechtbauer W, Dominguez M-G, Penn DL, van Os J, Krabbendam L (2011). The relationship between neurocognition and social cognition with functional outcomes in schizophrenia: a meta-analysis. Neurosci Biobehav Rev.

[7] Tse S, Chan S, Ng KL, Yatham LN (2014). Meta-analysis of predictors of favorable employment outcomes among individuals with bipolar disorder. Bipolar Disord.

[8] Baune BT, Malhi GS (2015). A review on the impact of cognitive dysfunction on social, occupational, and general functional outcomes in bipolar disorder. Bipolar Disord.

[9] Wang G, Si T-M, Li L, Fang Y, Wang C-X, Wang L-N (2019). Cognitive symptoms in major depressive disorder: associations with clinical and functional outcomes in a 6-month, non-interventional, prospective study in China. Neuropsychiatr Dis Treat.

[10] Nielsen RE, Levander S, Kjaersdam Telléus G, Jensen SOW, Østergaard Christensen T, Leucht S (2015). Second-generation antipsychotic effect on cognition in patients with schizophrenia–a meta-analysis of randomized clinical trials. Acta Psychiatr Scand.

[11] Clissold M, Crowe SF (2019). Comparing the effect of the subcategories of atypical antipsychotic medications on cognition in schizophrenia using a meta-analytic approach. J Clin Exp Neuropsychol.

[12] Vreeker A, van Bergen AH, Kahn RS (2015). Cognitive enhancing agents in schizophrenia and bipolar disorder. Eur Neuropsychopharmacol.

[13] Soria V, González-Rodríguez A, Huerta-Ramos E, Usall J, Cobo J, Bioque M (2018). Targeting hypothalamic-pituitary-adrenal axis hormones and sex steroids for improving cognition in major mood disorders and schizophrenia: a systematic review and narrative synthesis. Psychoneuroendocrinology.

[14] Baune BT, Brignone M, Larsen KG (2018). A network meta-analysis comparing effects of various antidepressant classes on the digit symbol substitution test (DSST) as a measure of cognitive dysfunction in patients with major depressive disorder. Int J Neuropsychopharmacol.

[15] Prado CE, Watt S, Crowe SF (2018). A meta-analysis of the effects of antidepressants on cognitive functioning in depressed and non-depressed samples. Neuropsychol Rev.

[16] Meyer-Lindenberg A, Miletich RS, Kohn PD, Esposito G, Carson RE, Quarantelli M (2002). Reduced prefrontal activity predicts exaggerated striatal dopaminergic function in schizophrenia. Nat Neurosci.

[17] Yang AC, Tsai S-J. New targets for schizophrenia treatment beyond the dopamine hypothesis. Int J Mol Sci. 2017;18:1689. 10.3390/ijms18081689 PMC5578079 28771182

[18] Reddy-Thootkur M, Kraguljac NV, Lahti AC. The role of glutamate and GABA in cognitive dysfunction in schizophrenia and mood disorders - a systematic review of magnetic resonance spectroscopy studies. Schizophr Res. 2020;S0920–9964:30077–300783. 10.1016/j.schres.2020.02.001 PMC7874516 32107102

[19] Salavati B, Rajji TK, Price R, Sun Y, Graff-Guerrero A, Daskalakis ZJ (2015). Imaging-based neurochemistry in schizophrenia: a systematic review and implications for dysfunctional long-term potentiation. Schizophr Bull.

[20] Quisenaerts C, Morrens M, Hulstijn W, de Bruijn E, Timmers M, Streffer J (2014). The nicotinergic receptor as a target for cognitive enhancement in schizophrenia: barking up the wrong tree?. Psychopharmacology.

[21] Ferrer A, Labad J, Salvat-Pujol N, Monreal JA, Urretavizcaya M, Crespo JM (2020). Hypothalamic-pituitary-adrenal axis-related genes and cognition in major mood disorders and schizophrenia: a systematic review. Prog Neuropsychopharmacol Biol Psychiatry.

[22] Sanada K, Zorrilla I, Iwata Y, Bermúdez-Ampudia C, Graff-Guerrero A, Martínez-Cengotitabengoa M et al. The efficacy of non-pharmacological interventions on brain-derived neurotrophic factor in schizophrenia: a systematic review and meta-analysis. Int J Mol Sci 2016;17:1766. 10.3390/ijms17101766 PMC5085790 27783051

[23] Goldsmith DR, Rapaport MH, Miller BJ (2016). A meta-analysis of blood cytokine network alterations in psychiatric patients: comparisons between schizophrenia, bipolar disorder and depression. Mol Psychiatry.

[24] De Picker L, Fransen E, Coppens V, Timmers M, de Boer P, Oberacher H (2019). Immune and neuroendocrine trait and state markers in psychotic illness: decreased kynurenines marking psychotic exacerbations. Front Immunol.

[25] Morrens M, Coppens V, Walther S (2020). Do immune dysregulations and oxidative damage drive mood and psychotic disorders?. Neuropsychobiology.

[26] De Picker LJ, Morrens M, Chance SA, Boche D (2017). Microglia and brain plasticity in acute psychosis and schizophrenia illness course: a meta-review. Front Psychiatry.

[27] Plavén-Sigray P, Matheson GJ, Coughlin JM, Hafizi S, Laurikainen H, Ottoy J (2021). Meta-analysis of the glial marker TSPO in psychosis revisited: reconciling inconclusive findings of patient-control differences. Biol Psychiatry.

[28] De Picker LJ, Haarman BCM. Applicability, potential and limitations of TSPO PET imaging as a clinical immunopsychiatry biomarker. Eur J Nucl Med Mol Imaging 2021;49:164–173. 10.1007/s00259-021-05308-0 33735406

[29] Morrens M, De Picker L, Kampen JK, Coppens V (2020). Blood-based kynurenine pathway alterations in schizophrenia spectrum disorders: a meta-analysis. Schizophr Res.

[30] Hebbrecht K, Skorobogatov K, Giltay EJ, Coppens V, De Picker L, Morrens M (2021). Tryptophan catabolites in bipolar disorder: a meta-analysis. Front Immunol.

[31] Karageorgiou V, Milas GP, Michopoulos I (2019). Neutrophil-to-lymphocyte ratio in schizophrenia: a systematic review and meta-analysis. Schizophr Res.

[32] Çakici N, van Beveren NJM, Judge-Hundal G, Koola MM, Sommer IEC (2019). An update on the efficacy of anti-inflammatory agents for patients with schizophrenia: a meta-analysis. Psychol Med.

[33] Köhler-Forsberg O, N Lydholm C, Hjorthøj C, Nordentoft M, Mors O, Benros ME (2019). Efficacy of anti-inflammatory treatment on major depressive disorder or depressive symptoms: meta-analysis of clinical trials. Acta Psychiatr Scand.

[34] Rosenblat JD, Kakar R, Berk M, Kessing LV, Vinberg M, Baune BT (2016). Anti-inflammatory agents in the treatment of bipolar depression: a systematic review and meta-analysis. Bipolar Disord.

[35] Khandaker GM, Dantzer R (2016). Is there a role for immune-to-brain communication in schizophrenia?. Psychopharmacology.

[36] Capuron L, Miller AH (2011). Immune system to brain signaling: neuropsychopharmacological implications. Pharm Ther.

[37] Rosenblat JD, Brietzke E, Mansur RB, Maruschak NA, Lee Y, McIntyre RS (2015). Inflammation as a neurobiological substrate of cognitive impairment in bipolar disorder: evidence, pathophysiology and treatment implications. J Affect Disord.

[38] Stuart MJ, Baune BT (2014). Chemokines and chemokine receptors in mood disorders, schizophrenia, and cognitive impairment: a systematic review of biomarker studies. Neurosci Biobehav Rev.

[39] Misiak B, Stańczykiewicz B, Kotowicz K, Rybakowski JK, Samochowiec J, Frydecka D (2018). Cytokines and C-reactive protein alterations with respect to cognitive impairment in schizophrenia and bipolar disorder: a systematic review. Schizophr Res.

[40] Ribeiro-Santos A, Lucio Teixeira A, Salgado JV (2014). Evidence for an immune role on cognition in schizophrenia: a systematic review. Curr Neuropharmacol.

[41] Chakrabarty T, Torres IJ, Bond DJ, Yatham LN (2019). Inflammatory cytokines and cognitive functioning in early-stage bipolar I disorder. J Affect Disord.

[42] Barbosa IG, Rocha NP, Huguet RB, Ferreira RA, Salgado JV, Carvalho LA (2012). Executive dysfunction in euthymic bipolar disorder patients and its association with plasma biomarkers. J Affect Disord.

[43] Barbosa IG, Ferreira R, de A, Rocha NP, Mol GC, da Mata Chiaccjio Leite F (2018). Predictors of cognitive performance in bipolar disorder: The role of educational degree and inflammatory markers. J Psychiatr Res.

[44] Asevedo E, Rizzo LB, Gadelha A, Mansur RB, Ota VK, Berberian AA (2014). Peripheral interleukin-2 level is associated with negative symptoms and cognitive performance in schizophrenia. Physiol Behav.

[45] Bobińska K, Gałecka E, Szemraj J, Gałecki P, Talarowska M (2017). Is there a link between TNF gene expression and cognitive deficits in depression?. Acta Biochim Pol.

[46] Page MJ, McKenzie JE, Bossuyt PM, Boutron I, Hoffmann TC, Mulrow CD (2021). The PRISMA 2020 statement: an updated guideline for reporting systematic reviews. BMJ.

[47] Peterson RA, Brown SP (2005). On the use of beta coefficients in meta-analysis. J Appl Psychol.

[48] Green MF, Nuechterlein KH, Gold JM, Barch DM, Cohen J, Essock S (2004). Approaching a consensus cognitive battery for clinical trials in schizophrenia: the NIMH-MATRICS conference to select cognitive domains and test criteria. Biol Psychiatry.

[49] Silver NC, Dunlap WP (1987). Averaging correlation coefficients: should Fisher’s z transformation be used?. J Appl Psychol.

[50] Miller GE, White SF, Chen E, Nusslock R (2021). Association of inflammatory activity with larger neural responses to threat and reward among children living in poverty. Am J Psychiatry.

[51] Beydoun MA, Obhi HK, Weiss J, Canas JA, Beydoun HA, Evans MK (2020). Systemic inflammation is associated with depressive symptoms differentially by sex and race: a longitudinal study of urban adults. Mol Psychiatry.

[52] Sirivichayakul S, Kanchanatawan B, Thika S, Carvalho AF, Maes M (2019). A new schizophrenia model: immune activation is associated with the induction of different neurotoxic products which together determine memory impairments and schizophrenia symptom dimensions. CNS Neurol Disord Drug Targets.

[53] Mehta ND, Stevens JS, Li Z, Gillespie CF, Fani N, Michopoulos V (2020). Inflammation, reward circuitry and symptoms of anhedonia and PTSD in trauma-exposed women. Soc Cogn Affect Neurosci.

[54] Altendahl M, Maillard P, Harvey D, Cotter D, Walters S, Wolf A (2020). An IL-18-centered inflammatory network as a biomarker for cerebral white matter injury. PLoS One.

[55] Nandkeolyar S, Naqvi A, Fan W, Sharma A, Rana JS, Rozanski A (2019). Utility of novel serum biomarkers to predict subclinical atherosclerosis: a sub-analysis of the EISNER study. Atherosclerosis.

[56] Tait JL, Duckham RL, Milte CM, Main LC, Daly RM (2019). Associations between inflammatory and neurological markers with quality of life and well-being in older adults. Exp Gerontol.

[57] Osterlund P, Orpana A, Elomaa I, Repo H, Joensuu H (2002). Raltitrexed treatment promotes systemic inflammatory reaction in patients with colorectal carcinoma. Br J Cancer.

[58] Wirsching J, Graßmann S, Eichelmann F, Harms LM, Schenk M, Barth E (2018). Development and reliability assessment of a new quality appraisal tool for cross-sectional studies using biomarker data (BIOCROSS). BMC Med Res Methodol.

[59] Hedges LV, Hedges LV, Olkin I. Statistical methods for meta-analysis. Academic Press, 1985.

[60] Evans JD. Straightforward Statistics for the behavioral sciences. Thomson Brooks/Cole, 1996.

[61] Tipton E, Pustejovsky JE, Ahmadi H (2019). Current practices in meta-regression in psychology, education, and medicine. Res Synth Methods.

[62] Higgins JPT, Thomas J (2019). Cochrane Handbook for Systematic Reviews of Interventions.

[63] Ali NS, Hashem AHH, Hassan AM, Saleh AA, El-Baz HN (2018). Serum interleukin-6 is related to lower cognitive functioning in elderly patients with major depression. Aging Ment Health.

[64] Asevedo E, Gadelha A, Noto C, Mansur RB, Zugman A, Belangero SIN (2013). Impact of peripheral levels of chemokines, BDNF and oxidative markers on cognition in individuals with schizophrenia. J Psychiatr Res.

[65] Amin MM, Rasyid A, Effendy E, Amir N, Suryandari DA (2020). The level of tumour necrosis factor-alpha and its relationship to the cognitive function of Malayan-Mongoloid patients with schizophrenia. Med Glas.

[66] Belge J-B, Van Diermen L, Sabbe B, Morrens M, Coppens V, de Timary P (2021). Inflammatory markers may inform the effects of electroconvulsive therapy on cognition in patients with depression. Neuropsychobiology.

[67] Boozalis T, Teixeira AL, Cho RY-J, Okusaga O (2017). C-reactive protein correlates with negative symptoms in patients with schizophrenia. Front Public Health.

[68] Borovcanin MM, Minic Janicijevic S, Jovanovic IP, Gajovic NM, Jurisevic MM, Arsenijevic NN. Type 17 immune response facilitates progression of inflammation and correlates with cognition in stable schizophrenia. Diagnostics (Basel) 2020;10:926. 10.3390/diagnostics10110926 PMC7698203 33182582

[69] Cathomas F, Guetter K, Seifritz E, Klaus F, Kaiser S (2021). Quinolinic acid is associated with cognitive deficits in schizophrenia but not major depressive disorder. Sci Rep.

[70] Chang HH, Lee IH, Gean PW, Lee S-Y, Chi MH, Yang YK (2012). Treatment response and cognitive impairment in major depression: association with C-reactive protein. Brain Behav Immun.

[71] Chung K-H, Huang S-H, Wu J-Y, Chen P-H, Hsu J-L, Tsai S-Y (2013). The link between high-sensitivity C-reactive protein and orbitofrontal cortex in euthymic bipolar disorder. Neuropsychobiology.

[72] Dal Santo F, González-Blanco L, García-Álvarez L, de la Fuente-Tomás L, Velasco Á, Álvarez-Vázquez CM (2020). Cognitive impairment and C-reactive protein in clinically stable schizophrenia outpatients: a focus on sex differences. Sci Rep.

[73] Doganavsargil-Baysal O, Cinemre B, Aksoy UM, Akbas H, Metin O, Fettahoglu C (2013). Levels of TNF-α, soluble TNF receptors (sTNFR1, sTNFR2), and cognition in bipolar disorder. Hum Psychopharmacol.

[74] Elderkin-Thompson V, Irwin MR, Hellemann G, Kumar A (2012). Interleukin-6 and memory functions of encoding and recall in healthy and depressed elderly adults. Am J Geriatr Psychiatry.

[75] Ergün S, Yanartaş Ö, Kandemir G, Yaman A, Yıldız M, Haklar G (2018). The relationship between psychopathology and cognitive functions with cytokines in clinically stable patients with schizophrenia. Psyc Clin Psychopharmacol.

[76] Fang X, Wang Y, Chen Y, Ren J, Zhang C (2019). Association between IL-6 and metabolic syndrome in schizophrenia patients treated with second-generation antipsychotics. Neuropsychiatr Dis Treat.

[77] Fathian F, Løberg E-M, Gjestad R, Steen VM, Kroken RA, Jørgensen HA (2019). Associations between C-reactive protein levels and cognition during the first 6 months after acute psychosis. Acta Neuropsychiatr.

[78] Frydecka D, Misiak B, Pawlak-Adamska E, Karabon L, Tomkiewicz A, Sedlaczek P (2015). Interleukin-6: the missing element of the neurocognitive deterioration in schizophrenia? The focus on genetic underpinnings, cognitive impairment and clinical manifestation. Eur Arch Psychiatry Clin Neurosci.

[79] Frydecka D, Misiak B, Pawlak-Adamska E, Karabon L, Tomkiewicz A, Sedlaczek P (2015). Sex differences in TGFB-β signaling with respect to age of onset and cognitive functioning in schizophrenia. NDT.

[80] Grassi-Oliveira R, Bauer ME, Pezzi JC, Teixeira AL, Brietzke E (2011). Interleukin-6 and verbal memory in recurrent major depressive disorder. Neuro Endocrinol Lett.

[81] Hori H, Yoshimura R, Katsuki A, Atake K, Igata R, Konishi Y (2017). Relationships between serum brain-derived neurotrophic factor, plasma catecholamine metabolites, cytokines, cognitive function and clinical symptoms in Japanese patients with chronic schizophrenia treated with atypical antipsychotic monotherapy. World J Biol Psychiatry.

[82] Huang J, Tong J, Zhang P, Zhou Y, Cui Y, Tan S (2021). Effects of neuroactive metabolites of the tryptophan pathway on working memory and cortical thickness in schizophrenia. Transl Psychiatry.

[83] Johnsen E, Fathian F, Kroken RA, Steen VM, Jørgensen HA, Gjestad R (2016). The serum level of C-reactive protein (CRP) is associated with cognitive performance in acute phase psychosis. BMC Psychiatry.

[84] Kindler J, Lim CK, Weickert CS, Boerrigter D, Galletly C, Liu D (2020). Dysregulation of kynurenine metabolism is related to proinflammatory cytokines, attention, and prefrontal cortex volume in schizophrenia. Mol Psychiatry.

[85] King S, Holleran L, Mothersill D, Patlola S, Rokita K, McManus R (2021). Early life Adversity, functional connectivity and cognitive performance in Schizophrenia: The mediating role of IL-6. Brain Behav Immun.

[86] Klaus F, Mitchell K, Liou SC, Eyler LT, Nguyen TT (2021). Chemokine MCP1 is associated with cognitive flexibility in schizophrenia: a preliminary analysis. J Psychiatr Res.

[87] Kogan S, Ospina LH, Kimhy D (2018). Inflammation in individuals with schizophrenia - Implications for neurocognition and daily function. Brain Behav Immun.

[88] Kudo N, Yamamori H, Ishima T, Nemoto K, Yasuda Y, Fujimoto M (2018). Plasma levels of soluble tumor necrosis factor receptor 2 (sTNFR2) are associated with hippocampal volume and cognitive performance in patients with schizophrenia. Int J Neuropsychopharmacol.

[89] Lizano P, Lutz O, Ling G, Lee AM, Eum S, Bishop JR (2019). Association of choroid plexus enlargement with cognitive, inflammatory, and structural phenotypes across the psychosis spectrum. Am J Psychiatry.

[90] Lizano P, Lutz O, Xu Y, Rubin LH, Paskowitz L, Lee AM (2021). Multivariate relationships between peripheral inflammatory marker subtypes and cognitive and brain structural measures in psychosis. Mol Psychiatry.

[91] Martínez-Cengotitabengoa M, Mac-Dowell KS, Leza JC, Micó JA, Fernandez M, Echevarría E (2012). Cognitive impairment is related to oxidative stress and chemokine levels in first psychotic episodes. Schizophr Res.

[92] Millett CE, Perez-Rodriguez M, Shanahan M, Larsen E, Yamamoto HS, Bukowski C (2019). C-reactive protein is associated with cognitive performance in a large cohort of euthymic patients with bipolar disorder. Mol Psychiatry.

[93] Milton DC, Ward J, Ward E, Lyall DM, Strawbridge RJ, Smith DJ (2021). The association between C-reactive protein, mood disorder, and cognitive function in UK Biobank. Eur Psychiatry.

[94] Mora E, Portella MJ, Piñol-Ripoll G, López R, Cuadras D, Forcada I (2019). High BDNF serum levels are associated to good cognitive functioning in bipolar disorder. Eur Psychiatry.

[95] Moustafa SR, Al-Rawi KF, Stoyanov D, Al-Dujaili AH, Supasitthumrong T, Al-Hakeim HK et al. The endogenous opioid system in schizophrenia and treatment resistant schizophrenia: increased plasma endomorphin 2, and κ and μ opioid receptors are associated with Interleukin-6. Diagnostics (Basel) 2020;10:633. 10.3390/diagnostics10090633 PMC7554941 32858974

[96] North HF, Bruggemann J, Cropley V, Swaminathan V, Sundram S, Lenroot R (2021). Increased peripheral inflammation in schizophrenia is associated with worse cognitive performance and related cortical thickness reductions. Eur Arch Psychiatry Clin Neurosci.

[97] Noyan H, Erdağ E, Tüzün E, Yaylım İ, Küçükhüseyin Ö, Hakan MT (2021). Association of the kynurenine pathway metabolites with clinical, cognitive features and IL-1β levels in patients with schizophrenia spectrum disorder and their siblings. Schizophr Res.

[98] Orhan F, Fatouros-Bergman H, Schwieler L, Cervenka S, Flyckt L, Sellgren CM (2018). First-episode psychosis patients display increased plasma IL-18 that correlates with cognitive dysfunction. Schizophr Res.

[99] Peters AT, Ren X, Bessette KL, Goldstein BI, West AE, Langenecker SA (2019). Interplay between pro-inflammatory cytokines, childhood trauma, and executive function in depressed adolescents. J Psychiatr Res.

[100] Qu N, Zhang S-F, Xia B, Xie J-Z, Wang X-M, Liu J (2019). Sex difference in IL-6 modulation of cognition among Chinese individuals with major depressive disorder. J Clin Neurosci.

[101] Rahmani N, Hatch J, Dimick M, Naiberg MR, Fiksenbaum L, Andreazza AC (2021). Lower pro- to anti-inflammatory ratios associated with reduced neurocognitive flexibility in symptomatic adolescents with bipolar disorder. J Affect Disord.

[102] Rebouças DB, Rabelo-da-Ponte FD, Massuda R, Czepielewski LS, Gama CS (2018). The relationship between cytokines and verbal memory in individuals with schizophrenia and their unaffected siblings. Neuroimmunomodulation.

[103] Ribeiro-Santos R, de Campos-Carli SM, Ferretjans R, Teixeira-Carvalho A, Martins-Filho OA, Teixeira AL (2020). The association of cognitive performance and IL-6 levels in schizophrenia is influenced by age and antipsychotic treatment. Nord J Psychiatry.

[104] Sanchez-Autet M, Arranz B, Safont G, Sierra P, Garcia-Blanco A, de la Fuente L (2018). Gender differences in C-reactive protein and homocysteine modulation of cognitive performance and real-world functioning in bipolar disorder. J Affect Disord.

[105] Smagula SF, Lotrich FE, Aizenstein HJ, Diniz BS, Krystek J, Wu GF (2017). Immunological biomarkers associated with brain structure and executive function in late-life depression: exploratory pilot study. Int J Geriatr Psychiatry.

[106] Saraykar S, Cao B, Barroso LS, Pereira KS, Bertola L, Nicolau M (2018). Plasma IL-17A levels in patients with late-life depression. Braz J Psychiatry.

[107] Strawbridge R, Carter R, Saldarini F, Tsapekos D, Young AH Inflammatory biomarkers and cognitive functioning in individuals with euthymic bipolar disorder: exploratory study. BJPsych Open 2021;7.E126. 10.1192/bjo.2021.966 PMC8281256 36043690

[108] Tanaka T, Matsuda T, Hayes LN, Yang S, Rodriguez K, Severance EG (2017). Infection and inflammation in schizophrenia and bipolar disorder. Neurosci Res.

[109] Tateishi H, Setoyama D, Kang D, Matsushima J, Kojima R, Fujii Y (2021). The changes in kynurenine metabolites induced by rTMS in treatment-resistant depression: a pilot study. J Psychiatr Res.

[110] Zazula R, Dodd S, Dean OM, Berk M, Bortolasci CC, Verri WA (2021). Cognition-immune interactions between executive function and working memory, tumour necrosis factor-alpha (TNF-alpha) and soluble TNF receptors (sTNFR1 and sTNFR2) in bipolar disorder. World J Biol Psychiatry.

[111] Tseng H-H, Chang HH, Wei S-Y, Lu T-H, Hsieh Y-T, Yang YK (2021). Peripheral inflammation is associated with dysfunctional corticostriatal circuitry and executive dysfunction in bipolar disorder patients. Brain Behav Immun.

[112] Vinberg M, Weikop P, Olsen NV, Kessing LV, Miskowiak K (2016). Effect of recombinant erythropoietin on inflammatory markers in patients with affective disorders: a randomised controlled study. Brain Behav Immun.

[113] van den Ameele S, van Nuijs AL, Lai FY, Schuermans J, Verkerk R, van Diermen L (2020). A mood state-specific interaction between kynurenine metabolism and inflammation is present in bipolar disorder. Bipolar Disord.

[114] Weiser M, Levi L, Burshtein S, Chiriță R, Cirjaliu D, Gonen I (2019). The effect of minocycline on symptoms in schizophrenia: Results from a randomized controlled trial. Schizophr Res.

[115] Wilson KE, Demyanovich H, Rubin LH, Wehring HJ, Kilday C, Kelly DL (2018). Relationship of interferon-γ to cognitive function in midlife women with schizophrenia. Psychiatr Q.

[116] Wu JQ, Chen DC, Tan YL, Tan SP, Xiu MH, Wang ZR (2016). Altered interleukin-18 levels are associated with cognitive impairment in chronic schizophrenia. J Psychiatr Res.

[117] Xiu M-H, Wang D, Chen S, Du X-D, Chen D-C, Chen N (2018). Interleukin-3, symptoms and cognitive deficits in first-episode drug-naïve and chronic medicated schizophrenia. Psychiatry Res.

[118] Ye G, Yin GZ, Tang Z, Fu JL, Chen J, Chen SS (2018). Association between increased serum interleukin-6 levels and sustained attention deficits in patients with major depressive disorder. Psychol Med.

[119] Zhang L, Liu F, Zheng H, Wu R, Zhao J (2021). Serum Interleukin-1β and tumor necrosis factor-α in first-episode drug-naive and chronic schizophrenia patients: Associated with cognitive deficits. Asian J Psychiatr.

[120] Zhou Y, Zheng W, Liu W, Wang C, Zhan Y, Li H (2019). Cross-sectional relationship between kynurenine pathway metabolites and cognitive function in major depressive disorder. Psychoneuroendocrinology.

[121] Zhang XY, Tang W, Xiu MH, Chen DC, Yang FD, Tan YL (2013). Interleukin 18 and cognitive impairment in first episode and drug naïve schizophrenia versus healthy controls. Brain Behav Immun.

[122] Xiu MH, Tian L, Chen S, Tan YL, Chen DC, Chen J (2016). Contribution of IL-10 and its -592 A/C polymorphism to cognitive functions in first-episode drug-naive schizophrenia. Brain Behav Immun.

[123] Miller BJ, Herzig K-H, Jokelainen J, Karhu T, Keinänen-Kiukaanniemi S, Järvelin M-R (2021). Inflammation, hippocampal volume, and cognition in schizophrenia: results from the Northern Finland Birth Cohort 1966. Eur Arch Psychiatry Clin Neurosci.

[124] Dorofeikova M, Neznanov N, Petrova N (2018). Cognitive deficit in patients with paranoid schizophrenia: Its clinical and laboratory correlates. Psychiatry Res.

[125] Millet CE, Jarder J, Locascio JJ, Shanahan M, Santone G, Fichorova RN (2020). TNF-α and its soluble receptors mediate the relationship between prior severe mood episodes and cognitive dysfunction in euthymic bipolar disorder. Brain Behav Immun.

[126] Jin K, Lu J, Yu Z, Shen Z, Li H, Mou T (2020). Linking peripheral IL-6, IL-1β and hypocretin-1 with cognitive impairment from major depression. J Affect Disord.

[127] Joseph J, Depp C, Martin AS, Daly RE, Glorioso DK, Palmer BW (2015). Associations of high sensitivity C-reactive protein levels in schizophrenia and comparison groups. Schizophr Res.

[128] de Campos-Carli SM, Miranda AS, Dias ICS, de Oliveira A, Cruz BF, Vieira ÉLM (2017). Serum levels of interleukin-33 and its soluble form receptor (sST2) are associated with cognitive performance in patients with schizophrenia. Compr Psychiatry.

[129] Goldsmith DR, Haroon E, Woolwine BJ, Jung MY, Wommack EC, Harvey PD (2016). Inflammatory markers are associated with decreased psychomotor speed in patients with major depressive disorder. Brain Behav Immun.

[130] Malmqvist A, Schwieler L, Orhan F, Fatouros-Bergman H, Bauer M, Flyckt L (2019). Increased peripheral levels of TARC/CCL17 in first episode psychosis patients. Schizophr Res.

[131] Bora E (2019). Peripheral inflammatory and neurotrophic biomarkers of cognitive impairment in schizophrenia: a meta-analysis. Psychol Med.

[132] Macêdo DS, Araújo DP, Sampaio LRL, Vasconcelos SMM, Sales PMG, Sousa FCF (2012). Animal models of prenatal immune challenge and their contribution to the study of schizophrenia: a systematic review. Braz J Med Biol Res.

[133] Blanco Ayala TB, Ramírez Ortega DR, Ovalle Rodríguez PO, Pineda B, Pérez de la Cruz GP de la, González Esquivel DG et al. Subchronic -acetylcysteine treatment decreases brain kynurenic acid levels and improves cognitive performance in mice. Antioxidants (Basel) 2021;10:147. 10.3390/antiox10020147 PMC7909398 33498402

[134] Kozak R, Campbell BM, Strick CA, Horner W, Hoffmann WE, Kiss T (2014). Reduction of brain kynurenic acid improves cognitive function. J Neurosci.

[135] Zhang L, Zheng H, Wu R, Kosten TR, Zhang X-Y, Zhao J (2019). The effect of minocycline on amelioration of cognitive deficits and pro-inflammatory cytokines levels in patients with schizophrenia. Schizophr Res.

[136] Levkovitz Y, Mendlovich S, Riwkes S, Braw Y, Levkovitch-Verbin H, Gal G (2010). A double-blind, randomized study of minocycline for the treatment of negative and cognitive symptoms in early-phase schizophrenia. J Clin Psychiatry.

[137] Jeppesen R, Christensen RHB, Pedersen EMJ, Nordentoft M, Hjorthøj C, Köhler-Forsberg O, Benros ME. Efficacy and safety of anti-inflammatory agents in treatment of psychotic disorders – a comprehensive systematic review and meta-analysis. Brain Behav Immun 2020;90:364–80. 10.1016/j.bbi.2020.08.028 32890697

[138] Bogaty P, Dagenais GR, Joseph L, Boyer L, Leblanc A, Bélisle P (2013). Time variability of C-reactive protein: implications for clinical risk stratification. PLoS One.

[139] Nuechterlein KH, Ventura J, Subotnik KL, Bartzokis G (2014). The early longitudinal course of cognitive deficits in schizophrenia. J Clin Psychiatry.

[140] Orsolini L, Sarchione F, Vellante F, Fornaro M, Matarazzo I, Martinotti G (2018). Protein-C reactive as biomarker predictor of schizophrenia phases of illness? a systematic review. Curr Neuropharmacol.

[141] Capuzzi E, Bartoli F, Crocamo C, Clerici M, Carrà G (2017). Acute variations of cytokine levels after antipsychotic treatment in drug-naïve subjects with a first-episode psychosis: a meta-analysis. Neurosci Biobehav Rev.

[142] Miller BJ, Buckley P, Seabolt W, Mellor A, Kirkpatrick B (2011). Meta-analysis of cytokine alterations in schizophrenia: clinical status and antipsychotic effects. Biol Psychiatry.

[143] Fernandes BS, Steiner J, Bernstein H-G, Dodd S, Pasco JA, Dean OM (2016). C-reactive protein is increased in schizophrenia but is not altered by antipsychotics: meta-analysis and implications. Mol Psychiatry.

[144] Skorobogatov K, De Picker L, Verkerk R, Coppens V, Leboyer M, Müller N (2021). Brain blood: a systematic review on the concordance between peripheral and central kynurenine pathway measures in psychiatric disorders. Front Immunol.

[145] Felger JC, Haroon E, Patel TA, Goldsmith DR, Wommack EC, Woolwine BJ (2020). What does plasma CRP tell us about peripheral and central inflammation in depression?. Mol Psychiatry.

[146] Tsai S-J (2017). Effects of interleukin-1beta polymorphisms on brain function and behavior in healthy and psychiatric disease conditions. Cytokine Growth Factor Rev.

[147] Prins BP, Abbasi A, Wong A, Vaez A, Nolte I, Franceschini N (2016). Investigating the causal relationship of c-reactive protein with 32 complex somatic and psychiatric outcomes: a large-scale cross-consortium mendelian randomization study. PLoS Med.

[148] Atwater AQ, Immergluck LC, Davidson AJ, Castanon-Cervantes O. Shift work predicts increases in lipopolysaccharide-binding protein, interleukin-10, and leukocyte counts in a cross-sectional study of healthy volunteers carrying low-grade systemic inflammation. Int J Environ Res Public Health 2021;18,13158. 10.3390/ijerph182413158 PMC8701724 34948768

[149] Subhi Y, Krogh Nielsen M, Molbech CR, Oishi A, Singh A, Nissen MH (2019). Plasma markers of chronic low-grade inflammation in polypoidal choroidal vasculopathy and neovascular age-related macular degeneration. Acta Ophthalmol.

[150] Field AP (2001). Meta-analysis of correlation coefficients: a Monte Carlo comparison of fixed- and random-effects methods. Psychol Methods.

[151] van den Ameele S, van Diermen L, Staels W, Coppens V, Dumont G, Sabbe B (2016). The effect of mood-stabilizing drugs on cytokine levels in bipolar disorder: a systematic review. J Affect Disord.

