# Supplementary material for: The relationship between immune and cognitive dysfunction in mood and psychotic disorder: a systematic review and a meta-analysis
Source: Mol Psychiatry. 2022 Apr 28;27(8):3237–46. doi: 10.1038/s41380-022-01582-y (PMC9708549; doi:10.1038/s41380-022-01582-y)
Supplement: Supplementary file 1 — Supplementary Materials [file 41380_2022_1582_MOESM1_ESM.pdf]

Supplementary Table 1: PRISMA Checklist

Section and Topic	Item #	Checklist item	Location where item is reported
**TITLE**			
Title	1	Identify the report as a systematic review.	Title
**ABSTRACT**			
Abstract	2	See the PRISMA 2020 for Abstracts checklist.	Abstract
**INTRODUCTION**			
Rationale	3	Describe the rationale for the review in the context of existing knowledge.	Introduction
Objectives	4	Provide an explicit statement of the objective(s) or question(s) the review addresses.	Introduction
**METHODS**			
Eligibility criteria	5	Specify the inclusion and exclusion criteria for the review and how studies were grouped for the syntheses.	Methods and materials, search strategy
Information sources	6	Specify all databases, registers, websites, organisations, reference lists and other sources searched or consulted to identify studies. Specify the date when each source was last searched or consulted.	Methods and materials, search strategy
Search strategy	7	Present the full search strategies for all databases, registers and websites, including any filters and limits used.	Supplementary table 2
Selection process	8	Specify the methods used to decide whether a study met the inclusion criteria of the review, including how many reviewers screened each record and each report retrieved, whether they worked independently, and if applicable, details of automation tools used in the process.	Methods and materials, search strategy, Supplementary Figure 1
Data collection process	9	Specify the methods used to collect data from reports, including how many reviewers collected data from each report, whether they worked independently, any processes for obtaining or confirming data from study investigators, and if applicable, details of automation tools used in the process.	Methods and materials, data extraction and evaluation
Data items	10a	List and define all outcomes for which data were sought. Specify whether all results that were compatible with each outcome domain in each study were sought (e.g. for all measures, time points, analyses), and if not, the methods used to decide which results to collect.	Methods and materials, data extraction and evaluation
	10b	List and define all other variables for which data were sought (e.g. participant and intervention characteristics, funding sources). Describe any assumptions made about any missing or unclear information.	Methods and materials, data extraction and evaluation
Study risk of bias assessment	11	Specify the methods used to assess risk of bias in the included studies, including details of the tool(s) used, how many reviewers assessed each study and whether they worked independently, and if applicable, details of automation tools used in the process.	Methods and materials, data extraction and evaluation
Effect measures	12	Specify for each outcome the effect measure(s) (e.g. risk ratio, mean difference) used in the synthesis or presentation of results.	Methods and materials, data extraction and

Section and Topic	Item #	Checklist item	Location where item is reported
			evaluation
Synthesis methods	13a	Describe the processes used to decide which studies were eligible for each synthesis (e.g. tabulating the study intervention characteristics and comparing against the planned groups for each synthesis (item #5)).	Methods and materials, data extraction and evaluation
	13b	Describe any methods required to prepare the data for presentation or synthesis, such as handling of missing summary statistics, or data conversions.	Methods and materials, data extraction and evaluation
	13c	Describe any methods used to tabulate or visually display results of individual studies and syntheses.	Methods and materials, data extraction and evaluation, Supplementary table 5-7
	13d	Describe any methods used to synthesize results and provide a rationale for the choice(s). If meta-analysis was performed, describe the model(s), method(s) to identify the presence and extent of statistical heterogeneity, and software package(s) used.	Methods and materials, Data synthesis and analysis
	13e	Describe any methods used to explore possible causes of heterogeneity among study results (e.g. subgroup analysis, meta-regression).	Methods and materials, Data synthesis and analysis
	13f	Describe any sensitivity analyses conducted to assess robustness of the synthesized results.	Methods and materials, Data synthesis and analysis
Reporting bias assessment	14	Describe any methods used to assess risk of bias due to missing results in a synthesis (arising from reporting biases).	Methods and materials, data extraction and evaluation
Certainty assessment	15	Describe any methods used to assess certainty (or confidence) in the body of evidence for an outcome.	Methods and materials, Data synthesis and analysis
**RESULTS**			
Study selection	16a	Describe the results of the search and selection process, from the number of records identified in the search to the number of studies included in the review, ideally using a flow diagram.	Supplementary Figure 1 PRISMA Flowchart
	16b	Cite studies that might appear to meet the inclusion criteria, but which were excluded, and explain why they were excluded.	Supplementary Figure 1 PRISMA Flowchart
Study characteristics	17	Cite each included study and present its characteristics.	Results, study selection
Risk of bias in studies	18	Present assessments of risk of bias for each included study.	Results, assessment of publication bias, quality

Section and Topic	Item #	Checklist item	Location where item is reported
			assessment, Supplementary table 8
Results of individual studies	19	For all outcomes, present, for each study: (a) summary statistics for each group (where appropriate) and (b) an effect estimate and its precision (e.g. confidence/credible interval), ideally using structured tables or plots.	Results, supplementary figures 2-13
Results of syntheses	20a	For each synthesis, briefly summarise the characteristics and risk of bias among contributing studies.	Tables 1-4
	20b	Present results of all statistical syntheses conducted. If meta-analysis was done, present for each the summary estimate and its precision (e.g. confidence/credible interval) and measures of statistical heterogeneity. If comparing groups, describe the direction of the effect.	Tables 1-4
	20c	Present results of all investigations of possible causes of heterogeneity among study results.	Results, covariate assessment, assessment of publication bias supplementary figures 14-16
	20d	Present results of all sensitivity analyses conducted to assess the robustness of the synthesized results.	Tables 1-4
Reporting biases	21	Present assessments of risk of bias due to missing results (arising from reporting biases) for each synthesis assessed.	Results, assessment of publication bias supplementary figures 14-16
Certainty of evidence	22	Present assessments of certainty (or confidence) in the body of evidence for each outcome assessed.	Results, quality assesment
**DISCUSSION**			
Discussion	23a	Provide a general interpretation of the results in the context of other evidence.	Discussion
	23b	Discuss any limitations of the evidence included in the review.	Discussion, limitations section
	23c	Discuss any limitations of the review processes used.	Discussion, limitations section
	23d	Discuss implications of the results for practice, policy, and future research.	Discussion
**OTHER INFORMATION**			
	24a	Provide registration information for the review, including register name and registration number, or state that the review was not registered.	Methods and materials
	24b	Indicate where the review protocol can be accessed, or state that a protocol was not prepared.	Protocol as supplementary document
	24c	Describe and explain any amendments to information provided at registration or in the protocol.	N/A
Support	25	Describe sources of financial or non-financial support for the review, and the role of the funders or sponsors in the	Disclosures

Section and Topic	Item #	Checklist item	Location where item is reported
		review.	
Competing interests	26	Declare any competing interests of review authors.	Disclosures
Availability of data, code and other materials	27	Report which of the following are publicly available and where they can be found: template data collection forms; data extracted from included studies; data used for all analyses; analytic code; any other materials used in the review.	Collected data are not publlcly available

Supplementary Table 2: Search strings on Pubmed, Web of Science and Psycinfo

platform	Search strategy
Pubmed	(schizophreni* OR schizoaffective OR psychotic OR psychosis OR MDD OR “depressive disorder” OR “major depression” OR manic OR mania OR bipolar OR “mood disorder” OR “affective disorder” OR dysthym*) AND (immun* OR chemokine* OR cytokine* OR interleukin* OR CRP OR C-reactive OR IL-1b OR IL-1β OR IL-1ra OR IL-2 OR IL-4 OR IL-6 OR IL-8 OR IL-10 OR IL-12 OR “tumor necrosis factor” OR TNF OR IFN OR IFN-γ OR Interferon* OR kynuren* OR quinolinic OR xanthurenic OR picolinic OR anthranilic OR HLA OR lymphocyt* OR monocyt* OR Leukocyt* OR neutrophil* OR basophil* or eosinophil*) AND (cogniti* OR attenti* OR vigilan* OR “executive functioning” OR reasoning OR “problem solving” OR memory OR learning OR “processing speed”) NOT (review [Publication type])
Web of Science	(ALL=(schizophreni*) OR ALL=(schizoaffective) OR ALL=(psychotic) OR ALL=(psychosis) OR ALL=(MDD) OR ALL=(depressive disorder) OR ALL=(major depression) OR ALL=(manic) OR ALL=(mania) OR ALL=(bipolar) OR ALL=(mood disorder) OR ALL=(affective disorder) OR ALL=(dysthym*)) AND (ALL=(immun*) OR ALL=(chemokine*) OR ALL=(cytokine*) OR ALL=(interleukin*) OR ALL=(CRP) OR ALL=(C-reactive) OR ALL=(IL-1b) OR ALL=(IL-1β) OR ALL=(IL-1ra) OR ALL=(IL-2) OR ALL=(IL-4) OR ALL=(IL-6) OR ALL=(IL-8) OR ALL=(IL-10) OR ALL=(IL-12) OR ALL=(tumor necrosis factor) OR ALL=(TNF) OR ALL=(IFN) OR ALL=(IFN-γ) OR ALL=(Interferon*) OR ALL=(kynuren*) OR ALL=(quinolinic) OR ALL=(xanthurenic) OR ALL=(picolinic) OR ALL=(anthranilic) OR ALL=(HLA) OR ALL=(lymphocyt*) OR ALL=(monocyt*) OR ALL=(leukocyt*) OR ALL=(neutrophil*) OR ALL=(basophil*) OR ALL=(eosinophil*)) AND (ALL=(cognitive*) OR ALL=(attenti*) OR ALL=(vigilan*) OR ALL=(executive functioning) OR ALL=(reasoning) OR ALL=(problem solving) OR ALL=(memory) OR ALL=(learning) OR ALL=(processing speed))
Psycinfo	**Any Field**: schizophrenia *OR* **Any Field**: schizophreniform *OR* **Any Field**: psychotic *OR* **Any Field**: psychosis *OR* **Any Field**: MDD *OR* **Any Field**: depressive disorder *OR* **Any Field**: major depression *OR* **Any Field**: manic *OR* **Any Field**: mania *OR* **Any Field**: bipolar *OR* **Any Field**: mood disorder *OR* **Any Field**: affective disorder *OR* **Any Field**: dysthymia *AND* **Any Field**: Immune OR cytokine OR chemokine OR interleukin OR CRP OR IL-1b OR IL-1β OR IL-1ra OR IL-2 OR IL-4 OR IL-6 OR IL-8 OR IL-10 OR IL-12 OR tumor necrosis factor OR TNF OR IFN OR IFN-γ OR Interferon OR kynurenine OR kynurenic OR quinolinic OR xanthurenic OR picolinic OR anthranilic OR HLA OR lymphocyte OR monocyte OR Leukocyte OR neutrophil OR basophil or eosinophil *AND* **Any Field**: cognition *OR* **Any Field**: cognitive *OR* **Any Field**: attention *OR* **Any Field**: vigilance *OR* **Any Field**: executive function *OR* **Any Field**: executive functioning *OR* **Any Field**: reasoning *OR* **Any Field**: problem solving *OR* **Any Field**: memory *OR* **Any Field**: learning *OR* **Any Field**: processing speed *AND* **Peer-Reviewed Journals only**

Supplementary Table 3: Investigated cognitive domains and cognitive tests included

Domain	Tests
Verbal learning and memory	List learning tasks (RBANS-memory, BACS, CVLT, RaVLT,…)
Visual learning and memory	RBANS-visuospatial; pairs matching test;
Working memory	LNS, Letter sequencing, Digit backward, spatial span test, Digit sequencing test, BACS-WM, MCCB-WM, delayed match to sample test,
Attention and vigilance	RBANS-attention, MCCB-attention, BACS-attention, CANTAB-attention, CPT, Digit span, simple reaction time
Processing speed	Digit Symbol Coding tests, TMTB
Reasoning and problem solving	WCST, Tower of London, CANTAB-set shift, Stroop test, Mazes, BACS-EF, abstraction test, matrix reasoning, MCCB reasoning,
Language	RBANS-language, Verbal Fluency tests, COWAT, naming test

Supplementary Table 4: Included inflammatory markers

Category	Inflammatory markers
Pro-inflammatory markers	(hs)CRP, IL-1b, IL-2, IL-3, IL-6, IL-7, IL-8, IL-12-p70, IL-15, IL-16, IL-17, IL-18, IL-33, IFN-g, TNF-a, TNF-b, sTNFR1, sTNFR2, CCL-11, CCL-17, CCL-22, CXCL-10, MCP-1, sST2
Anti-inflammatory markers	IL-1ra, IL-4, IL-10, IL-11, IL-13, TGFb

Supplementary Table 5: Demographic characteristics of included studies

Study Name	year	Diagnosis	N	mean age	DOI	BMI	%mP	smoking%
Ali	2018	MDD	40	64,1	29,4	24,01	53	NR
Amin	2020	SZ	50	35,4	NR	21,6	76	NR
Asevedo	2013	SZ	30	33,7	NR	NR	83	48
Asevedo	2014	SZ	29	33,2	11,8	NR	83	46
Barbosa	2018	BD	20	43,8	20,2	NR	25	NR
Barbosa	2012	BD	25	50,9	27,9	29,1	32	NR
Belge	2021	MDD	62	58,2	NR	24,6	23	21
Bobinska	2016	MDD	89	39,9	7	NR	44	NR
Boozalis	2018	SZ	39	32,8	NR	27,3	74	NR
Borovcanin	2020	SZ	27	36,2	9,95	25,4	59	NR
Cathomas	2021	MDD	44	35,8	7	22,9	40	23
		SZ	45	34	10,1	26,4	69	64
Chakrabarty	2018	BD	51	23,2	25,3	25,2	47	NR
Chang	2012	MDD	112	38,8	NR	NR	28	16
Chung	2013	BD	17	31,3	8,3	23,3	23,3	41
Dal Santo	2020	SZ	132	37,8	8,93	28,2	62	49
de Campos-Carli	2017	SZ	40	40,8	11,9	NR	53	NR
De Picker	2020	SZ	49	32,4	6,1	24,9	86	64
Doganavsargil-Baysal	2013	BD	54	39,5	12,5	NR	33	NR
Dorofeikova	2018	SZ	125	35,8	12	NR	58	NR
Elderkin-Thompson	2012	MDD	42	69,7	NR	25,6	33	NR
Ergun	2018	SZ	30	36,4	11,4	27,2	70	53
Fang	2019	SZ	174	35,8	NR	24,1	47	NR
Fathian	2019	SZ	208	33,5	NR	23,7	69	NR

Frydecka	2015a	SZ	151	37,8	12,2	NR	46	NR
Frydecka	2015b	SZ	151	37,8	12,2	NR	46	NR
Goldsmith	2016	MDD	93	39,4	NR	31,5	NR	NR
Grassi-Oliveira	2011	MDD	30	39,2	11,5	25,8	NR	NR
Hori	2016	SZ	146	33,6	8,7	NR	51	36
Huang	2020	SZ	30	27,6	2,9	NR	50	NR
Jin	2020	MDD	100	27,8	NR	NR	38	NR
Johnsen	2016	SZ	124	33,5	NR	23,5	68	52
Joseph	2015	SZ	88	49,4	23,6	31	39,4	49
Kindler	2020	SZ	96	35,7	12,9	NR	61	49
King	2021	SZ	104	42	NR	29,5	70	NR
Klaus	2021	SZ	16	42	NR	NR	25	NR
Kogan	2018	SZ	32	37	NR	31,1	64	24
Kudo	2018	SZ	87	42,5	12,3	NR	50	NR
Lizano	2019	SZ	223	35,1	15,5	NR	66	NR
		BD	190	36,1	19,5	NR	31	NR
Lizano	2021	SZ	140	34,3	18,5	NR	43	NR
Malmqvist	2019	SZ	41	28	0,5	21,6	63	8
Martinez-Cengotitabengoa	2012	SZ	28	27,7	0	NR	75	NR
Miller	2020	SZ	31	31	10,4	25,8	61	45
Millet	2019	BD	222	44	NR	29,9	53	NR
Millet	2020	BD	219	44	22	29,9	53	NR
Milton	2021	BD	969	55,5	NR	28,2	54	NR
		MDD	22441	56	NR	27,8	51	NR
Mora	2018	BD	84	45,1	NR	28	52	NR
Moustafa	2020	SZ	115	36,3	8	28,9	68	NR

North	2021	SZ	499	39,6	15,8	NR	65	72
Noyan	2021	SZ	150	31,6	5,6	25,2	75	56
Orhan	2018	SZ	25	28,5	NR	21,8	71	26
Peters	2019	MDD	40	14,5	2,4	NR	40	NR
Qu	2019	MDD	104	34,1	9,3	22,7	36	19
Rahmani	2021	BD	26	17,6	1,9	21,9	42	15
Reboucas	2018	SZ	35	40	NR	26,1	69	43
Ribeiro-Santos	2019	SZ	63	42,4	15,9	25,6	52,4	30,1
Sanchez-autet	2018	BD	224	47,1	19,5	28,5	34,7	45,2
Saraykar	2018	MDD	74	73	NR	26,6	9	NR
Smagula	2017	MDD	31	68,8	NR	30,9	77	NR
Strawbridge	2021	BD	44	43,7	NR	27,8	30	NR
Tanaka	2017	BD	32	39	NR	NR	64	39
Tateishi	2020	MDD	11	52,2	6	NR	55	NR
Tseng	2019	BD	24	36,5	NR	NR	36	24
Van den Ameele	2020	BD	67	43,1	NR	25,3	48	36
Vinberg	2016	MDD	44	43	NR	26	69	NR
Weiser	2019	SZ	200	43	13	NR	44	NR
Wilson	2018	SZ	14	47	NR	40	0	57
Wu	2016	SZ	70	46,2	21,5	24,9	41	68
Xiu	2016	SZ	128	26,4	1	20,1	59	28
Xiu	2018	SZ	45	26,4	1	20,1	59	28
Ye	2018	MDD	30	42,6	5,8	22,9	40	17
Zazula	2021	BD	31	40	19	28	19	NR
Zhang	2013	SZ	77	29,2	2,7	22,6	52	25
Zhang	2021	SZ	75	NR	NR	NR	NR	NR
Zhou	2021	MDD	149	39	NR	22	22	15

Supplementary Table 6: included immunomarkers in the included studies

Study Name	year	Diagnosis	# markers	Inflammation marker
Ali	2018	MDD	1	IL-6
Amin	2020	SZ	1	TNF-a
Asevedo	2013	SZ	6	CXCL-10/IP-10, CXCL-8/IL-8, CCL-11, CCL-24/Eotaxin-2, CCL-2/MCP-1, CCL-3/MIP-1
Asevedo	2014	SZ	1	IL-2
Barbosa	2018	BD	9	IL2, IL4,IL6,IL10,IFNg,TNFa,IL17a,sTNFR1,sTNFR2
Barbosa	2012	BD	3	TNF-a, TNFR1, sTNFR2
Belge	2021	MDD	7	IL6,IL1b,IFNg,CRP,IL8,TNFa, IL-10
Bobinska	2016	MDD	3	TNFa, TNFRSF1A, TNFRS1B
Boozalis	2018	SZ	1	CRP
Borovcanin	2020	SZ	4	IL-17, IL-6, TNF-a; sST2)
Cathomas	2021	MDD, SZ	5	TRYCATS
Chakrabarty	2018	BD	5	IL1b,IL4,IL6,IL10,TNFa
Chang	2012	MDD	1	CRP
Chung	2013	BD	1	hs-CRP
Dal Santo	2020	SZ	1	CRP
de Campos-Carli	2017	SZ	2	IL-33, sST2
De Picker	2020	SZ	12	TRYCATS, CRP, IL6
Doganavsargil-Baysal	2013	BD	3	TNFa, sTNFR1, sTNFR2
Dorofeikova	2018	SZ	1	CRP
Elderkin-Thompson	2012	MDD	2	CRP, IL-6
Ergun	2018	SZ	2	IL-6, TNFa
Fang	2019	SZ	1	IL-6
Fathian	2019	SZ	1	CRP

Frydecka	2015a	SZ	2	hs-CRP, IL-6
Frydecka	2015b	SZ	1	TGF-b
Goldsmith	2016	MDD	8	TNF, IL1b, IL6,IL10, MCP1,IL1RA,IL6sr, TNFr2
Grassi-Oliveira	2011	MDD	2	IL-6, TNFa
Hori	2016	SZ	2	IL-6, TNF-a
Huang	2020	SZ	3	TRP, KYN, KYNA
Jin	2020	MDD	2	IL-1b, IL-6
Johnsen	2016	SZ	1	CRP
Joseph	2015	SZ	1	hs-CRP
Kindler	2020	SZ	10	TRP, KYN, KYNA, 3HK, QUINA, CRP, IL-1, IL6, IL-8, IL-18
King	2021	SZ	1	IL6
Klaus	2021	SZ	13	CRP, IL6, IL-10, IP-10, IFNg, TNFa, Eotaxin, MCP1, MIP1b, Fractalkine, SAA, ICAM1, VCAM1
Kogan	2018	SZ	4	IL-1b, IL-6, TNFa, IL-12p70
Kudo	2018	SZ	1	sTNFR2
Lizano	2019	SZ, BD	12	CRP, IL6, TNFa, IL1a, IL1b, IL4, IL8, IL10, IL12, IL23, IL12p70, IFNg
Lizano	2021	SZ	12	CRP, IL6, TNFa, IL1a, IL1b, IL4, IL8, IL10, IL12, IL23, IL12p70, IFNg
Malmqvist	2019	SZ	4	TARC/CCL17, eotaxin/CCL11, MDC/CCL22, IP-10/CXCL10
Martinez-Cengotitabengoa	2012	SZ	1	MCP-1
Miller	2020	SZ	13	IL-6, CRP, IL17, IL1a, IL1b, IL1RA, IL4,IL6,IL8,IL10,MCP1,sCD40L,TNFa
Millet	2019	BD	1	CRP
Millet	2020	BD	3	TNFa sTNF-R1, sTNF-R2
Milton	2021	BD	1	CRP
Milton	2021	MDD	1	CRP
Mora	2018	BD	3	TNF-a, IL10, IL6
Moustafa	2020	SZ	3	IL6, IL10, CRP
North	2021	SZ	1	CRP

Noyan	2021	SZ	4	TRP, KYN, KYNA, IL-1b
Orhan	2018	SZ	1	IL-18
Peters	2019	MDD	3	IL6,IL1b, TNFa
Qu	2019	MDD	3	CRP, IL1b, IL6
Rahmani	2021	BD	6	CRP,IL1b, IL6, IL10, IL4, TNFa
Reboucas	2018	SZ	2	TNF-a, IL-6
Ribeiro-Santos	2019	SZ	7	IL2, IL4, IL6, IL10,IL17a,TNFa,IFNg
Sanchez-autet	2018	BD	1	CRP
Saraykar	2018	MDD	1	IL17A
Smagula	2017	MDD	7	TNFa, IL2, IL4, IL6, IL10, IL17, MCP-1
Strawbridge	2021	BD	19	CRP, IFNg, IL10, IL12, IL15,IL16,IL17,IL1a,IL6,IL7,IL8,IP10,MCP1,MCP4,MIP1a,MIP1b,TARC,SAA,TNFa,TNFb
Tanaka	2017	BD	1	CRP
Tateishi	2020	MDD	3	IL1b, IL6, TNFa
Tseng	2019	BD	1	CRP
Van den Ameele	2020	BD	10	CRP, TNF-α, IFN-γ, IL-1β, IL-4, IL-6, TRP, KYN, KYNA, QA
Vinberg	2016	MDD	3	hsCRP, IL18, IL6
Weiser	2019	SZ	1	CRP
Wilson	2018	SZ	1	IFNg
Wu	2016	SZ	1	I-18
Xiu	2016	SZ	1	IL-10
Xiu	2018	SZ	1	IL-3
Ye	2018	MDD	1	IL6
Zazula	2021	BD	3	TNF-a, sTNFR1, sTNFR2
Zhang	2013	SZ	3	IL-1b, IL-6, TNFa
Zhang	2021	SZ	2	IL-1b, TNFa
Zhou	2021	MDD	3	TRP, KYN, KA

Supplementary Table 7: Overview of the cognitive domains assessed per included study

Study Name	year	Diagnosis	VeM&L	ViM&L	WM	A&V	PS	La	R/P	SC	GC
Ali	2018	MDD	x			x	x	x			x
Amin	2020	SZ									x
Asevedo	2013	SZ	x		x		x	x	x		x
Asevedo	2014	SZ	x		x		x	x	x		x
Barbosa	2018	BD	x		x		x	x		x	x
Barbosa	2012	BD							x		
Belge	2021	MDD	x				x				
Bobinska	2016	MDD	x				x	x			x
Boozalis	2018	SZ	x	x	x	x	x	x			x
Borovcanin	2020	SZ									x
Cathomas	2021	MDD			x		x				x
Cathomas	2021	SZ			x		x				x
Chakrabarty	2018	BD	x	x	x	x	x	x		x	x
Chang	2012	MDD				x			x		x
Chung	2013	BD							x		
Dal Santo	2020	SZ	x	x	x	x	x	x	x	x	x
de Campos-Carli	2017	SZ									x
De Picker	2020	SZ			x		x				
Doganavsargil-Baysal	2013	BD	x						x		
Dorofeikova	2018	SZ	x		x		x		x		x
Elderkin-Thompson	2012	MDD	x	x		x	x		x		x
Ergun	2018	SZ	x	x	x	x	x	x	x		x
Fang	2019	SZ	x	x		x		x			x
Fathian	2019	SZ	x	x		x		x			x
Frydecka	2015a	SZ	x		x	x	x		x		x

Frydecka	2015b	SZ	x		x	x	x		x		x
Goldsmith	2016	MDD					x				
Grassi-Oliveira	2011	MDD	x								
Hori	2016	SZ	x		x	x	x		x		x
Huang	2020	SZ	x	x	x	x	x		x	x	x
Jin	2020	MDD	x	x	x	x	x		x	x	x
Johnsen	2016	SZ	x	x		x		x			x
Joseph	2015	SZ									
Kindler	2020	SZ	x		x	x	x				x
King	2021	SZ					x	x	x	x	x
Klaus	2021	SZ					x				
Kogan	2018	SZ	x	x	x	x	x		x	x	x
Kudo	2018	SZ	x	x							x
Lizano	2019	SZ	x		x	x	x	x	x	x	x
Lizano	2019	BD	x		x	x	x	x	x	x	x
Lizano	2021	SZ	x		x	x	x	x	x	x	x
Malmqvist	2019	SZ	x	x	x	x	x		x	x	x
Martinez-Cengotitabengoa	2012	SZ	x		x	x	x		x		x
Miller	2020	SZ	x								
Millet	2019	BD	x	x	x	x	x		x	x	x
Millet	2020	BD	x	x	x	x	x		x	x	x
Milton	2021	BD		x			x				x
Milton	2021	MDD		x			x				x
Mora	2018	BD	x	x		x	x	x			x
Moustafa	2020	SZ	x		x		x	x	x		x
North	2021	SZ	x	x		x		x			x
Noyan	2021	SZ					x		x		x
Orhan	2018	SZ	x	x	x	x	x		x	x	x

Peters	2019	MDD				x	x				
Qu	2019	MDD									x
Rahmani	2021	BD						x	x		x
Reboucas	2018	SZ	x								
Ribeiro-Santos	2019	SZ	x		x		x		x		x
Sanchez-autet	2018	BD	x		x		x	x			
Saraykar	2018	MDD	x	x		x			x		x
Smagula	2017	MDD					x				
Strawbridge	2021	BD	x		x		x		x		
Tanaka	2017	BD									x
Tateishi	2020	MDD					x	x	x		x
Tseng	2019	BD				x			x		x
Van den Ameele	2020	BD			x		x				x
Vinberg	2016	MDD	x								
Weiser	2019	SZ	x	x	x		x		x		x
Wilson	2018	SZ	x	x		x		x			x
Wu	2016	SZ	x	x		x		x			x
Xiu	2016	SZ	x	x		x		x			x
Xiu	2018	SZ	x	x		x		x			x
Ye	2018	MDD		x		x		x			
Zazula	2021	BD	x	x	x	x	x		x		x
Zhang	2013	SZ	x	x	x	x	x		x		x
Zhang	2021	SZ	x	x	x	x	x		x		x
Zhou	2021	MDD	x	x	x		x				x

Legend: VeM&L: Verbal Memory and Learning; ViM&L: Visual Memory and Learning; WM: Working Memory; A&V: Attention and Vigilance; PS: Processing Speed; La: Language; R/P: Reasoning/Problem Solving; SC: Social Cognition; GC: Global Cognition

Supplementary Table 8: BIOCROSS quality assessment of the included studies

**Study Name**	**year**	1. Hypo thesis	2. Study pop. sel	3. St pop rep	4. Study pop char	5. Stat. analysis	6. Context-specific disc.	7. Study limit.	8. assay methods	9. Labo meas.	10. Biomarker data modeling	SUM	**Quality**
Ali	2018	1	1	1	0	0	1	1	0	0	0	**5**	Moderate
Amin	2020	1	1	1	0	0	1	1	0	0	0	**5**	Moderate
Asevedo	2013	1	1	0	1	1	1	1	1	0	1	**8**	High
Asevedo	2014	1	1	0	1	1	1	1	0	0	0	**6**	Moderate
Barbosa	2018	1	1	0	0	1	1	1	1	0	0	**6**	Moderate
Barbosa	2012	1	1	0	1	0	1	0	1	0	1	**6**	Moderate
Belge	2021	1	1	0	0	0	1	0	1	0	0	**4**	Moderate
Bobinska	2016	1	1	0	0	0	1	0	1	0	0	**4**	Moderate
Boozalis	2018	1	1	0	1	1	1	1	0	0	0	**6**	Moderate
Borovcanin	2020	1	1	0	1	0	1	1	0	0	0	**5**	Moderate
Cathomas	2021	1	1	0	1	1	1	0	1	0	0	**6**	Moderate
Chakrabarty	2018	1	1	0	0	1	1	1	0	0	1	**6**	Moderate
Chang	2012	1	1	1	1	1	1	1	1	0	1	**9**	High
Chung	2013	1	1	0	1	1	1	0	1	0	0	**6**	Moderate
Dal Santo	2020	1	1	1	1	1	1	1	0	0	0	**7**	High
de Campos-Carli	2017	1	1	0	1	1	1	0	1	0	0	**6**	Moderate
De Picker	2020	1	1	0	1	1	1	1	1	0	1	**8**	High
Doganavsargil-Baysal	2013	1	1	1	1	1	1	1	0	0	1	**8**	High
Dorofeikova	2018	1	1	0	0	0	1	1	0	0	0	**4**	Moderate
Elderkin-Thompson	2012	1	1	0	1	1	1	1	1	0	0	**7**	High
Ergun	2018	1	1	0	1	1	1	1	0	0	1	**7**	High
Fang	2019	1	1	1	1	1	1	1	1	0	1	**9**	High
Fathian	2019	1	1	0	1	1	1	1	1	0	0	**7**	High

Frydecka	2015b	1	1	0	1	1	1	1	1	0	0	**7**	High
Frydecka	2015a	1	1	0	0	1	1	0	1	1	0	**6**	Moderate
Goldsmith	2016	1	1	0	0	1	1	0	1	0	0	**5**	Moderate
Grassi-Oliveira	2011	1	1	0	1	1	1	1	1	0	0	**7**	High
Hori	2016	1	1	1	0	1	1	1	1	0	0	**7**	High
Huang	2020	1	1	0	0	1	1	0	0	0	0	**4**	Moderate
Jin	2020	1	1	0	1	1	1	0	1	0	1	**7**	High
Johnsen	2016	1	1	0	1	1	1	0	1	0	0	**6**	Moderate
Joseph	2015	1	1	0	1	1	1	1	0	0	0	**6**	Moderate
Kindler	2020	1	1	0	0	0	1	1	1	0	1	**6**	Moderate
King	2021	1	1	0	1	1	1	1	1	0	0	**7**	High
Klaus	2021	1	1	0	0	1	1	1	0	0	0	**5**	Moderate
Kogan	2018	1	1	0	1	0	1	0	1	0	0	**5**	Moderate
Kudo	2018	1	1	0	1	1	1	0	1	1	1	**8**	High
Lizano	2019	1	1	0	1	1	1	1	1	0	0	**7**	High
Lizano	2021	1	1	0	1	1	1	1	1	1	1	**9**	High
Malmqvist	2019	1	1	0	1	1	1	0	1	1	0	**7**	High
Martinez-Cengotitabengoa	2012	1	1	0	0	1	1	0	1	0	0	**5**	Moderate
Miller	2020	1	1	1	1	1	1	0	1	0	0	**7**	High
Millet	2019	1	1	1	1	1	1	1	1	1	0	**9**	High
Millet	2020	1	1	0	1	1	1	0	1	0	0	**6**	Moderate
Milton	2021	1	1	1	1	1	1	0	1	0	0	**7**	High
Mora	2018	1	1	0	1	1	1	1	1	0	1	**8**	High
Moustafa	2020	1	1	1	1	1	1	0	1	0	1	**8**	High
North	2021	1	1	0	1	1	1	1	1	0	0	**7**	High
Noyan	2021	1	1	0	1	1	1	1	1	0	0	**7**	High
Orhan	2018	1	1	0	0	1	1	0	0	0	0	**4**	Low

Peters	2019	1	1	0	1	1	1	1	1	0	1	**8**	High
Qu	2019	1	1	0	1	1	1	1	1	0	0	**7**	High
Rahmani	2021	1	1	1	1	1	1	1	0	0	1	**8**	High
Reboucas	2018	1	1	0	1	1	1	0	1	0	0	**6**	Moderate
Ribeiro-Santos	2019	1	1	0	0	1	1	1	1	1	0	**7**	High
Sanchez-autet	2018	1	1	0	1	1	1	0	0	0	0	**5**	Moderate
Saraykar	2018	1	1	0	0	1	1	1	1	0	0	**6**	Moderate
Samagula	2017	1	1	0	1	1	1	1	1	0	1	**8**	High
Strawbridge	2021	1	1	0	1	1	1	1	1	0	1	**8**	High
Tanaka	2017	1	1	0	1	1	1	1	0	0	0	**6**	Moderate
Tateishi	2020	1	1	0	0	0	1	0	1	1	0	**5**	Moderate
Tseng	2019	1	1	0	1	1	1	1	0	0	0	**6**	Moderate
Van den Ameele	2020	1	1	0	0	1	1	1	1	0	1	**7**	High
Vinberg	2016	1	1	0	0	0	1	0	1	1	0	**5**	Moderate
Weiser	2019	1	1	1	0	1	1	1	1	0	0	**7**	High
Wilson	2018	1	1	1	1	1	1	0	0	0	0	**6**	Moderate
Wu	2016	1	1	0	1	1	1	1	1	1	0	**8**	High
Xiu	2016	1	1	0	1	1	1	1	1	0	0	**7**	High
Xiu	2018	1	1	0	1	1	1	0	0	1	0	**6**	Moderate
Ye	2018	1	1	0	0	0	1	1	1	1	1	**7**	High
Zazula	2021	1	1	0	1	1	1	0	0	0	0	**5**	Moderate
Zhang	2013	1	1	0	1	1	1	0	1	1	0	**7**	High
Zhang	2021	1	1	0	0	1	1	0	0	0	0	**4**	Low
Zhou	2021	1	1	0	1	1	1	0	1	0	0	**6**	Moderate

Supplementary Figure 1: PRISMA Flowchart

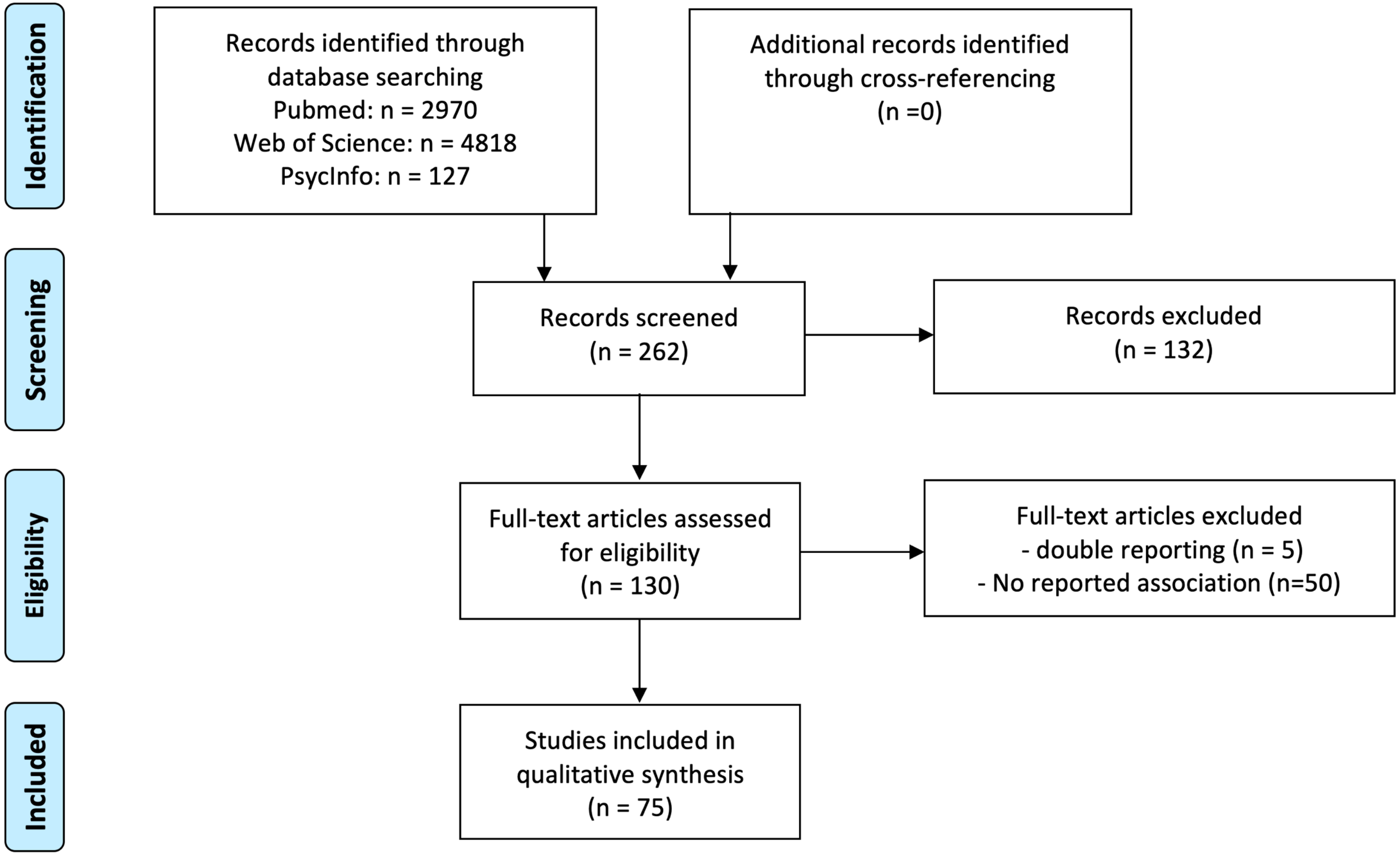

Supplementary Figure 2: Forest Plot for correlation studies between Pro Inflammatory Index (PII) and Global Cognition for all studies, as well as diagnostic subgroups (Bipolar Disorder (BD), Major Depressive Disorder (MDD), and schizophrenia spectrum disorders (SZ))

Group by Subgroup within study	Study name	Diagnosis	Statistics for each study Correlation	Lower limit	Upper limit	Z-Value	p-Value
BD	Lizano 2019	BD	-0.096	-0.235	0.047	-1.318	0.187
BD	Milton 2021	BD	-0.027	-0.089	0.037	-0.824	0.410
BD	Doganavsargil-Baysal 2013	BD	-0.108	-0.365	0.165	-0.774	0.439
BD	Tanaka 2017	BD	-0.001	-0.350	0.348	-0.005	0.996
BD	Barbosa 2018	BD	-0.461	-0.750	-0.023	-2.056	0.040
BD	Chakrabarty 2018	BD	-0.333	-0.557	-0.063	-2.399	0.016
BD	Mora 2018	BD	0.000	-0.214	0.215	0.002	0.999
BD	Sanchez-autet 2018	BD	-0.270	-0.387	-0.144	-4.116	0.000
BD	Millet 2019	BD	-0.017	-0.148	0.115	-0.244	0.807
BD	Tseng 2019	BD	-0.241	-0.587	0.180	-1.127	0.260
BD	Van den Ameele 2020	BD	-0.029	-0.267	0.213	-0.228	0.820
BD	Strawbridge 2021	BD	0.058	-0.243	0.349	0.374	0.708
BD	Zazula 2021	BD	0.070	-0.291	0.414	0.373	0.709
BD			-0.095	-0.168	-0.020	-2.474	0.013
MDD	Chang 2012	MDD	-0.211	-0.382	-0.026	-2.236	0.025
MDD	Elderkin-Thompson 2012	MDD	-0.250	-0.515	0.058	-1.595	0.111
MDD	Bobinska 2016	MDD	0.006	-0.203	0.214	0.056	0.956
MDD	Goldsmith 2016	MDD	-0.110	-0.307	0.096	-1.048	0.295
MDD	Ali 2018	MDD	-0.552	-0.737	-0.290	-3.779	0.000
MDD	Saraykar 2018	MDD	-0.290	-0.486	-0.066	-2.516	0.012
MDD	Ye 2018	MDD	0.115	-0.256	0.456	0.600	0.548
MDD	Peters 2019	MDD	0.139	-0.180	0.432	0.851	0.395
MDD	Qu 2019	MDD	-0.464	-0.603	-0.298	-5.049	0.000
MDD	Belge 2020	MDD	-0.044	-0.291	0.208	-0.338	0.735
MDD	Tateishi 2020	MDD	0.073	-0.551	0.645	0.207	0.836
MDD	Milton 2021	MDD	0.059	0.045	0.072	8.773	0.000
MDD	Zhou 2021	MDD	0.005	-0.156	0.166	0.059	0.953
MDD			-0.133	-0.257	-0.006	-2.051	0.040
SZ	Asevedo 2014	SZ	0.464	0.117	0.710	2.562	0.010
SZ	Frydecka 2015	SZ	0.175	0.016	0.326	2.151	0.031
SZ	Hori 2016	SZ	-0.029	-0.191	0.134	-0.347	0.729
SZ	Johnsen 2016	SZ	-0.247	-0.406	-0.074	-2.774	0.006
SZ	Wu 2016	SZ	0.292	0.061	0.493	2.462	0.014
SZ	de Campos-Carli 2017	SZ	0.316	0.005	0.571	1.990	0.047
SZ	Boozalis 2018	SZ	-0.030	-0.342	0.288	-0.180	0.857
SZ	Ergun 2018	SZ	0.023	-0.340	0.380	0.120	0.905
SZ	Kogan 2018	SZ	-0.292	-0.581	0.063	-1.620	0.105
SZ	Kudo 2018	SZ	0.040	-0.172	0.249	0.367	0.714
SZ	Wilson 2018	SZ	-0.390	-0.763	0.177	-1.366	0.172
SZ	Xiu 2018	SZ	0.080	-0.219	0.365	0.520	0.603
SZ	Fang 2019	SZ	-0.059	-0.206	0.091	-0.772	0.440
SZ	Fathian 2019	SZ	-0.370	-0.482	-0.246	-5.561	0.000
SZ	Lizano 2019	SZ	-0.020	-0.151	0.112	-0.292	0.770
SZ	Ribeiro-Santos 2019	SZ	-0.159	-0.391	0.092	-1.242	0.214
SZ	Weiser 2019	SZ	0.008	-0.091	0.108	0.165	0.869
SZ	Malmqvist 2019	SZ	0.020	-0.297	0.333	0.120	0.904
SZ	Amin 2020	SZ	-0.011	-0.288	0.268	-0.075	0.940
SZ	Borovcanin 2020	SZ	0.387	0.008	0.669	2.000	0.045
SZ	Dal Santo 2020	SZ	-0.148	-0.311	0.023	-1.693	0.090
SZ	De Picker 2020	SZ	-0.109	-0.379	0.178	-0.742	0.458
SZ	Lizano 2020	SZ	-0.256	-0.405	-0.094	-3.065	0.002
SZ	Moustafa 2020	SZ	0.178	-0.005	0.350	1.904	0.057
SZ	King 2021	SZ	-0.158	-0.340	0.036	-1.601	0.109
SZ	Klaus 2021	SZ	-0.394	-0.744	0.126	-1.502	0.133
SZ	Zhang 2021	SZ	-0.192	-0.401	0.037	-1.650	0.099
SZ			-0.036	-0.113	0.042	-0.897	0.370
Overall			-0.076	-0.126	-0.027	-3.011	0.003

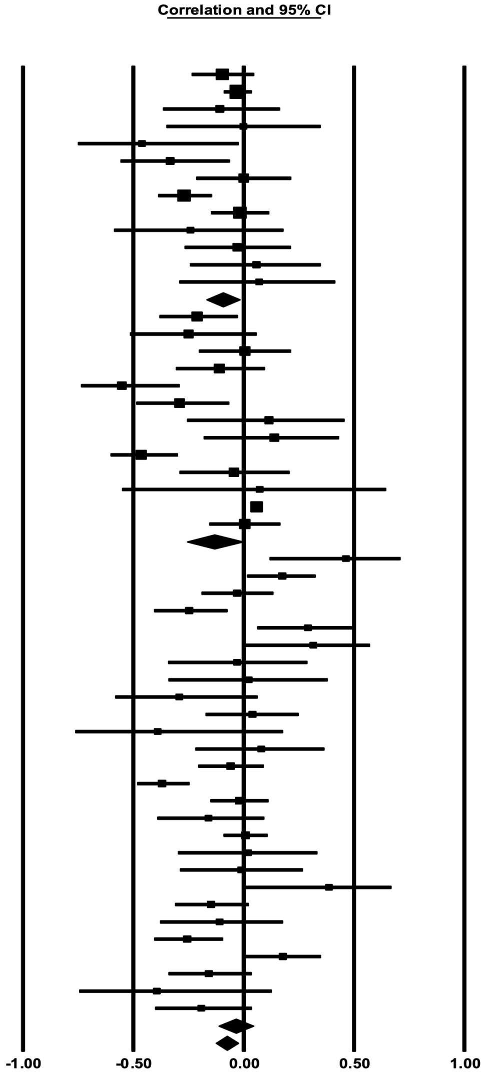

Supplementary Figure 3: Forest Plot for correlation studies between Pro Inflammatory Index (PII) and Cognitive Domain Attention for all studies, as well as diagnostic subgroups (Bipolar Disorder (BD), Major Depressive Disorder (MDD), and schizophrenia spectrum disorders (SZ))

Group by Subgroup within study	Study name	Diagnosis	Statistics for each study				
			Correlation	Lower limit	Upper limit	Z-Value	p-Value
BD	Milton 2021	BD	0.127	0.065	0.188	3.969	0.000
BD	Mora 2018	BD	0.108	-0.109	0.315	0.976	0.329
BD	Millet 2019	BD	-0.115	-0.243	0.017	-1.709	0.087
BD	Tseng 2019	BD	-0.048	-0.443	0.362	-0.220	0.826
BD	Vanden Ameele 2020	BD	-0.020	-0.259	0.221	-0.160	0.873
BD			0.025	-0.105	0.154	0.371	0.711
MDD	Chang 2012	MDD	-0.195	-0.367	-0.010	-2.062	0.039
MDD	Goldsmith 2016	MDD	-0.108	-0.305	0.098	-1.029	0.304
MDD	Samagula 2017	MDD	0.240	-0.125	0.548	1.295	0.195
MDD	Peters 2019	MDD	0.017	-0.296	0.327	0.103	0.918
MDD	Milton 2021	MDD	0.100	0.087	0.113	15.030	0.000
MDD	Zhou 2021	MDD	0.018	-0.143	0.178	0.218	0.828
MDD			0.003	-0.117	0.123	0.043	0.965
SZ	Asevedo 2014	SZ	0.416	0.058	0.679	2.258	0.024
SZ	Frydecka 2015	SZ	-0.143	-0.296	0.017	-1.752	0.080
SZ	Hori 2016	SZ	-0.010	-0.172	0.153	-0.120	0.905
SZ	de Campos-Carli 2017	SZ	0.316	0.005	0.571	1.990	0.047
SZ	Boozalis 2018	SZ	0.140	-0.184	0.436	0.846	0.398
SZ	Ergun 2018	SZ	0.020	-0.343	0.378	0.104	0.917
SZ	Kogan 2018	SZ	0.029	-0.323	0.374	0.158	0.875
SZ	Wilson 2018	SZ	-0.330	-0.732	0.243	-1.137	0.256
SZ	Fang 2019	SZ	-0.076	-0.222	0.074	-0.996	0.319
SZ	Fathian 2019	SZ	-0.380	-0.491	-0.257	-5.728	0.000
SZ	Malmqvist 2019	SZ	-0.119	-0.419	0.204	-0.717	0.473
SZ	Weiser 2019	SZ	-0.068	-0.166	0.032	-1.331	0.183
SZ	Dal Santo 2020	SZ	-0.132	-0.296	0.040	-1.508	0.132
SZ	Klaus 2021	SZ	0.487	-0.011	0.792	1.919	0.055
SZ	North 2021	SZ	-0.146	-0.231	-0.059	-3.275	0.001
SZ	Zhang 2021	SZ	-0.358	-0.541	-0.143	-3.179	0.001
SZ			-0.071	-0.164	0.022	-1.496	0.135
Overall			-0.027	-0.091	0.037	-0.820	0.412

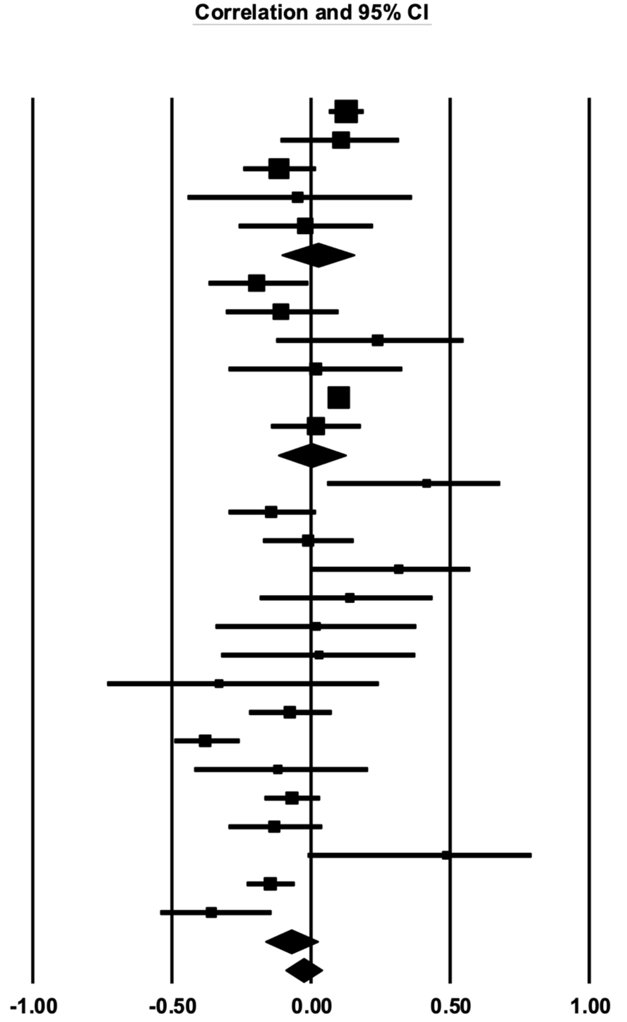

Supplementary Figure 4: Forest Plot for correlation studies between Pro Inflammatory Index (PII) and cognitive domain Language for all studies, as well as diagnostic subgroups (Bipolar Disorder (BD), Major Depressive Disorder (MDD), and schizophrenia spectrum disorders (SZ))

Group by Subgroup within study	Study name	Diagnosis	Statistics for each study: Correlation	Lower limit	Upper limit	Z-Value	p-Value
BD	Lizano 2019	BD	-0.065	-0.205	0.078	-0.890	0.373
BD	Sanchez-autet 2018	BD	-0.329	-0.441	-0.207	-5.080	0.000
BD	Strawbridge 2021	BD	-0.031	-0.325	0.268	-0.199	0.843
BD			-0.196	-0.283	-0.105	-4.198	0.000
MDD	Bobinska 2016	MDD	-0.064	-0.269	0.146	-0.594	0.552
MDD	Tateishi 2020	MDD	0.469	-0.182	0.834	1.439	0.150
MDD			-0.015	-0.214	0.185	-0.149	0.882
SZ	Frydecka 2015	SZ	-0.106	-0.261	0.055	-1.294	0.196
SZ	Zhang 2021	SZ	-0.354	-0.538	-0.138	-3.140	0.002
SZ	Hori 2016	SZ	-0.020	-0.182	0.143	-0.239	0.811
SZ	de Campos-Carli 20[illegible]	SZ	0.321	0.011	0.575	2.024	0.043
SZ	Boozalis 2018	SZ	-0.300	-0.562	0.017	-1.857	0.063
SZ	Ergun 2018	SZ	-0.088	-0.434	0.281	-0.458	0.647
SZ	Kudo 2018	SZ	0.140	-0.073	0.341	1.292	0.196
SZ	Fang 2019	SZ	-0.062	-0.209	0.088	-0.812	0.417
SZ	Fathian 2019	SZ	-0.350	-0.464	-0.225	-5.232	0.000
SZ	Lizano 2019	SZ	0.018	-0.114	0.149	0.260	0.795
SZ	Malmqvist 2019	SZ	0.058	-0.262	0.367	0.348	0.728
SZ	Weiser 2019	SZ	0.006	-0.094	0.106	0.121	0.903
SZ	Borovcanin 2020	SZ	0.569	0.241	0.780	3.165	0.002
SZ	Moustafa 2020	SZ	0.233	0.052	0.399	2.512	0.012
SZ	King 2021	SZ	-0.079	-0.276	0.125	-0.759	0.448
SZ	Orhan 2018	SZ	-0.380	-0.674	0.018	-1.876	0.061
SZ			-0.047	-0.093	-0.001	-2.016	0.044
Overall			-0.075	-0.115	-0.034	-3.631	0.000

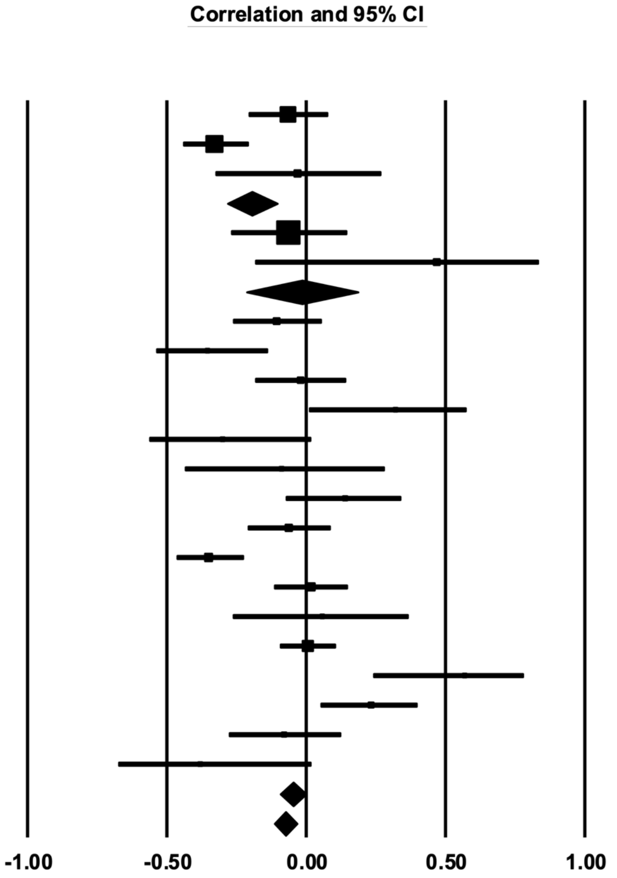

Supplementary Figure 5: Forest Plot for correlation studies between Pro Inflammatory Index (PII) and cognitive domain Processing Speed for all studies, as well as diagnostic subgroups (Bipolar Disorder (BD), Major Depressive Disorder (MDD), and schizophrenia spectrum disorders (SZ))

Group by Subgroup within study	Study name	Diagnosis	Statistics for each study				
			Correlation	Lower limit	Upper limit	Z-Value	p-Value
BD	Lizano 2019	BD	-0.065	-0.205	0.078	-0.890	0.373
BD	Chakrabarty 2018	BD	-0.562	-0.725	-0.339	-4.405	0.000
BD	Mora 2018	BD	0.065	-0.152	0.276	0.586	0.558
BD	Millet 2019	BD	-0.185	-0.309	-0.055	-2.770	0.006
BD	Strawbridge 2021	BD	0.149	-0.155	0.427	0.961	0.336
BD			-0.130	-0.318	0.067	-1.294	0.196
MDD	Bobinska 2016	MDD	-0.034	-0.241	0.175	-0.315	0.752
MDD	Goldsmith 2016	MDD	-0.110	-0.307	0.096	-1.048	0.295
MDD	Belge 2020	MDD	-0.044	-0.291	0.208	-0.338	0.735
MDD			-0.066	-0.191	0.062	-1.009	0.313
SZ	Frydecka 2015	SZ	-0.335	-0.470	-0.185	-4.239	0.000
SZ	Boozalis 2018	SZ	0.000	-0.316	0.316	0.000	1.000
SZ	Ergun 2018	SZ	0.063	-0.304	0.414	0.328	0.743
SZ	Kogan 2018	SZ	-0.364	-0.632	-0.018	-2.054	0.040
SZ	Lizano 2019	SZ	0.018	-0.114	0.149	0.260	0.795
SZ	Malmqvist 2019	SZ	0.056	-0.264	0.365	0.336	0.737
SZ	Ribeiro-Santos 2019	SZ	-0.024	-0.270	0.225	-0.186	0.852
SZ	Weiser 2019	SZ	0.066	-0.034	0.164	1.285	0.199
SZ	Dal Santo 2020	SZ	-0.140	-0.304	0.032	-1.601	0.109
SZ	De Picker 2020	SZ	-0.098	-0.369	0.188	-0.667	0.505
SZ	King 2021	SZ	-0.128	-0.322	0.077	-1.228	0.220
SZ	Zhang 2021	SZ	-0.066	-0.288	0.164	-0.557	0.578
SZ			-0.080	-0.171	0.013	-1.695	0.090
Overall			-0.082	-0.151	-0.012	-2.295	0.022

Correlation and 95% CI

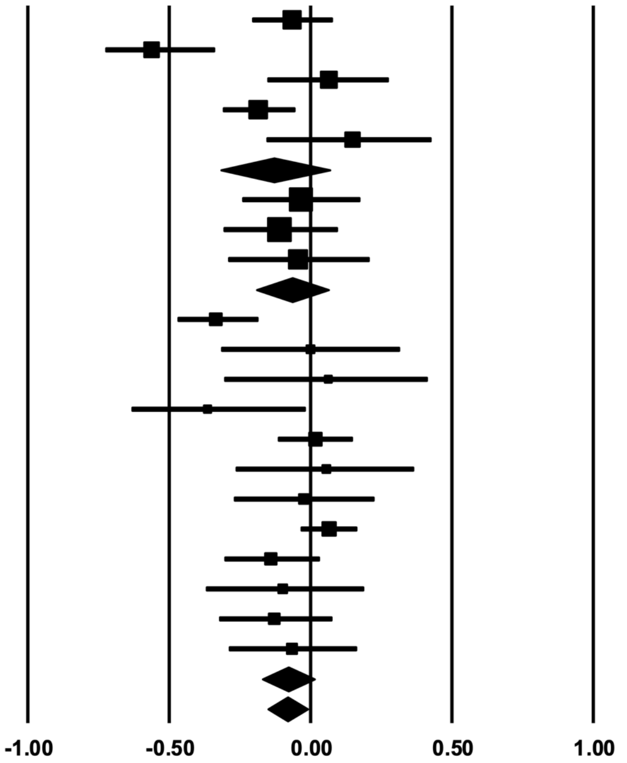

Supplementary Figure 6: Forest Plot for correlation studies between Pro Inflammatory Index (PII) and cognitive domain Reasoning for all studies, as well as diagnostic subgroups (Bipolar Disorder (BD), Major Depressive Disorder (MDD), and schizophrenia spectrum disorders (SZ))

Group by Subgroup within study	Study name	Diagnosis	Statistics for each study				
			Correlation	Lower limit	Upper limit	Z-Value	p-Value
BD	Lizano 2019	BD	-0.078	-0.217	0.066	-1.062	0.288
BD	Barbosa 2021	BD	0.500	0.131	0.747	2.576	0.010
BD	Doganavsargil-Baysal 2013	BD	-0.094	-0.353	0.178	-0.673	0.501
BD	Chakrabarty 2018	BD	0.259	-0.018	0.499	1.836	0.066
BD	Mora 2018	BD	0.061	-0.155	0.272	0.550	0.583
BD	Millet 2019	BD	-0.236	-0.357	-0.108	-3.560	0.000
BD	Tseng 2019	BD	-0.433	-0.717	-0.025	-2.073	0.038
BD	Millet 2020	BD	-0.200	-0.324	-0.069	-2.980	0.003
BD	Vanden Ameele 2020	BD	-0.030	-0.268	0.212	-0.240	0.810
BD	Rahmani 2021	BD	0.430	0.051	0.701	2.206	0.027
BD	Strawbridge 2021	BD	0.022	-0.277	0.317	0.141	0.888
BD	Zazula 2021	BD	0.200	-0.166	0.518	1.073	0.283
BD			0.002	-0.124	0.128	0.033	0.974
MDD	Chang 2012	MDD	-0.227	-0.396	-0.043	-2.412	0.016
MDD	Bobinska 2016	MDD	-0.163	-0.359	0.047	-1.525	0.127
MDD	Samagula 2017	MDD	0.055	-0.305	0.402	0.291	0.771
MDD	Ye 2018	MDD	0.120	-0.251	0.460	0.627	0.531
MDD	Peters 2019	MDD	0.247	-0.070	0.519	1.534	0.125
MDD	Tateishi 2020	MDD	-0.348	-0.784	0.318	-1.027	0.304
MDD	Zhou 2021	MDD	-0.017	-0.178	0.144	-0.210	0.833
MDD			-0.053	-0.183	0.079	-0.786	0.432
SZ	Asevedo 2013	SZ	0.510	0.183	0.735	2.924	0.003
SZ	Frydecka 2015	SZ	-0.257	-0.400	-0.101	-3.198	0.001
SZ	Joseph 2015	SZ	-0.030	-0.238	0.181	-0.277	0.782
SZ	Hori 2016	SZ	-0.025	-0.187	0.138	-0.299	0.765
SZ	de Campos-Carli 2017	SZ	0.216	-0.102	0.494	1.335	0.182
SZ	Boozalis 2018	SZ	0.010	-0.306	0.324	0.060	0.952
SZ	Ergun 2018	SZ	-0.056	-0.408	0.311	-0.289	0.773
SZ	Kogan 2018	SZ	-0.184	-0.501	0.176	-1.002	0.316
SZ	Kudo 2018	SZ	0.030	-0.182	0.239	0.275	0.783
SZ	Lizano 2019	SZ	-0.033	-0.163	0.099	-0.482	0.630
SZ	Malmqvist 2019	SZ	0.111	-0.212	0.412	0.669	0.504
SZ	Ribeiro-Santos 2019	SZ	-0.116	-0.354	0.136	-0.903	0.367
SZ	Weiser 2019	SZ	-0.040	-0.139	0.060	-0.782	0.434
SZ	Dal Santo 2020	SZ	-0.102	-0.268	0.070	-1.163	0.245
SZ	Moustafa 2020	SZ	0.200	0.018	0.370	2.146	0.032
SZ	King 2021	SZ	-0.194	-0.383	0.010	-1.864	0.062
SZ	Klaus 2021	SZ	-0.167	-0.612	0.358	-0.608	0.543
SZ	Zhang 2021	SZ	-0.031	-0.256	0.197	-0.263	0.792
SZ			-0.026	-0.096	0.045	-0.712	0.476
Overall			-0.025	-0.081	0.031	-0.882	0.378

Correlation and 95% CI

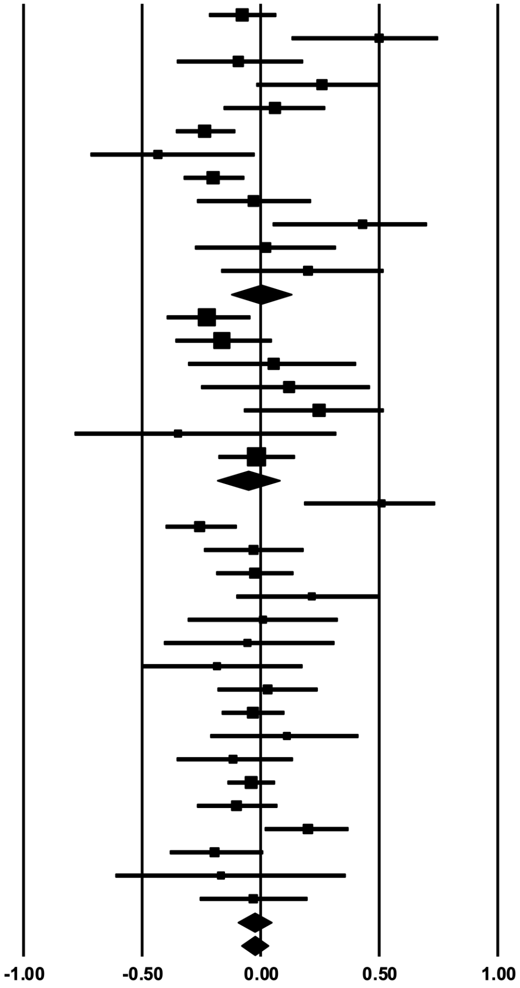

Supplementary Figure 7: Forest Plot for correlation studies between Pro Inflammatory Index (PII) and cognitive domain Verbal Memory for all studies, as well as diagnostic subgroups (Bipolar Disorder (BD), Major Depressive Disorder (MDD), and schizophrenia spectrum disorders (SZ))

Group by Subgroup within study	Study name	Diagnosis	Statistics for each study: Correlation	Lower limit	Upper limit	Z-Value	p-Value
BD	Lizano 2019	BD	-0.180	-0.314	-0.039	-2.489	0.013
BD	Doganavsargil-Baysal 2013	BD	-0.121	-0.377	0.152	-0.868	0.385
BD	Vinberg 2016	BD	-0.023	-0.318	0.276	-0.147	0.883
BD	Mora 2018	BD	0.076	-0.141	0.286	0.685	0.493
BD	Sanchez-autet 2018	BD	-0.208	-0.330	-0.079	-3.138	0.002
BD	Millet 2019	BD	-0.036	-0.167	0.096	-0.533	0.594
BD	Vanden Ameele 2020	BD	-0.012	-0.251	0.229	-0.096	0.924
BD	Strawbridge 2021	BD	0.083	-0.219	0.371	0.533	0.594
BD			-0.085	-0.164	-0.005	-2.082	0.037
MDD	Grassi-Oliveira 2011	MDD	-0.686	-0.839	-0.433	-4.367	0.000
MDD	Elderkin-Thompson 2012	MDD	-0.340	-0.584	-0.040	-2.211	0.027
MDD	Bobinska 2016	MDD	-0.111	-0.312	0.100	-1.034	0.301
MDD	Vinberg	MDD	0.019	-0.279	0.314	0.122	0.903
MDD	Belge 2020	MDD	-0.044	-0.291	0.208	-0.338	0.735
MDD	Zhou 2021	MDD	0.006	-0.155	0.167	0.077	0.938
MDD			-0.186	-0.375	0.018	-1.785	0.074
SZ	Martinez-Cengotitabengoa 2012	SZ	-0.382	-0.661	-0.010	-2.012	0.044
SZ	Frydecka 2015	SZ	-0.261	-0.404	-0.106	-3.250	0.001
SZ	Hori 2016	SZ	-0.045	-0.206	0.118	-0.538	0.590
SZ	Wu 2016	SZ	0.273	0.041	0.477	2.293	0.022
SZ	de Campos-Carli 2017	SZ	0.421	0.126	0.648	2.731	0.006
SZ	Boozalis 2018	SZ	-0.180	-0.469	0.144	-1.092	0.275
SZ	Dorofeikova 2018	SZ	-0.280	-0.434	-0.110	-3.178	0.001
SZ	Ergun 2018	SZ	0.012	-0.350	0.371	0.062	0.950
SZ	Kogan 2018	SZ	-0.134	-0.461	0.225	-0.726	0.468
SZ	Reboucas 2018	SZ	0.162	-0.181	0.470	0.925	0.355
SZ	Wilson 2018	SZ	0.382	-0.186	0.759	1.335	0.182
SZ	Xiu 2018	SZ	-0.250	-0.538	0.091	-1.445	0.149
SZ	Fang 2019	SZ	-0.054	-0.201	0.096	-0.707	0.480
SZ	Fathian 2019	SZ	-0.410	-0.517	-0.290	-6.237	0.000
SZ	Lizano 2019	SZ	-0.053	-0.183	0.079	-0.787	0.431
SZ	Malmqvist	SZ	0.055	-0.265	0.364	0.330	0.741
SZ	Weiser 2019	SZ	-0.001	-0.101	0.098	-0.025	0.980
SZ	Borovcanin 2020	SZ	0.416	0.043	0.687	2.169	0.030
SZ	Dal Santo 2020	SZ	-0.183	-0.343	-0.013	-2.102	0.036
SZ	Miller 2020	SZ	-0.406	-0.665	-0.060	-2.280	0.023
SZ	Moustafa 2020	SZ	0.238	0.057	0.404	2.568	0.010
SZ	Zhang 2021	SZ	-0.412	-0.584	-0.204	-3.717	0.000
SZ			-0.072	-0.172	0.030	-1.377	0.168
Overall			-0.089	-0.148	-0.029	-2.900	0.004

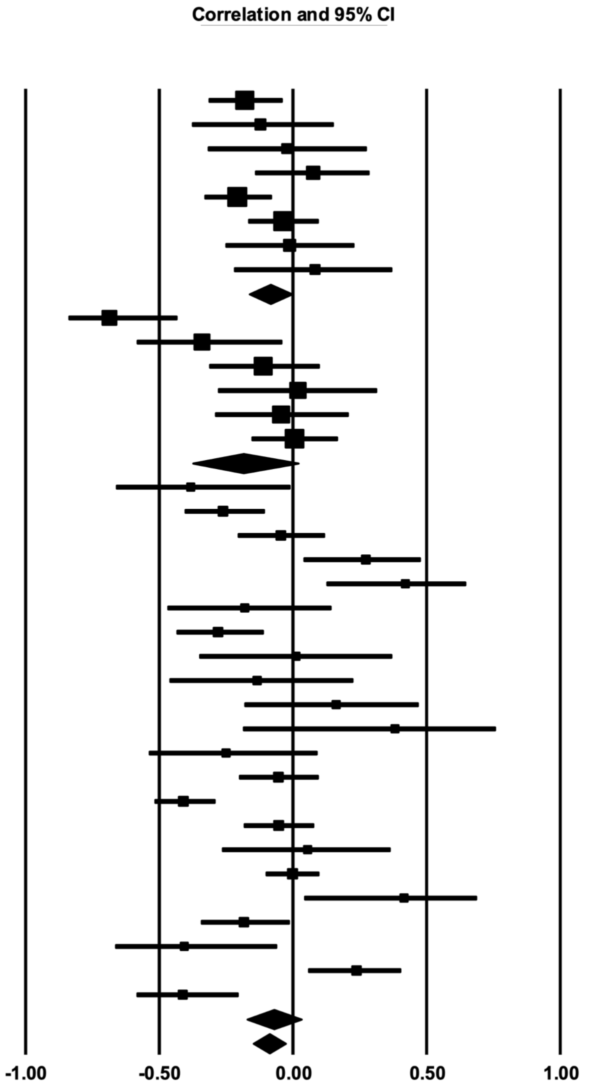

Supplementary Figure 8: Forest Plot for correlation studies between Pro Inflammatory Index (PII) and cognitive domain Visual Memory for all studies, as well as diagnostic subgroups (Bipolar Disorder (BD), Major Depressive Disorder (MDD), and schizophrenia spectrum disorders (SZ))

Group by Subgroup within study	Study name	Diagnosis	Statistics for each study				
			Correlation	Lower limit	Upper limit	Z-Value	p-Value
BD	Milton 2021	BD	-0.128	-0.213	-0.041	-2.875	0.004
BD	Mora 2018	BD	-0.108	-0.315	0.109	-0.976	0.329
BD	Millet 2019	BD	-0.140	-0.267	-0.008	-2.086	0.037
BD	Zazula 2021	BD	0.247	-0.118	0.553	1.335	0.182
BD			-0.108	-0.196	-0.019	-2.379	0.017
MDD	Milton 2021	MDD	-0.033	-0.046	-0.020	-4.945	0.000
MDD	Zhou 2021	MDD	-0.015	-0.176	0.146	-0.184	0.854
MDD			-0.033	-0.046	-0.020	-4.944	0.000
SZ	Zhang 2013	SZ	-0.112	-0.328	0.115	-0.968	0.333
SZ	Boozalis 2018	SZ	-0.240	-0.516	0.082	-1.469	0.142
SZ	Ergun 2018	SZ	0.030	-0.334	0.386	0.156	0.876
SZ	Kogan 2018	SZ	-0.342	-0.617	0.008	-1.919	0.055
SZ	Wilson 2018	SZ	-0.550	-0.836	-0.027	-2.051	0.040
SZ	Fang 2019	SZ	0.048	-0.101	0.195	0.628	0.530
SZ	Fathian 2019	SZ	-0.190	-0.318	-0.055	-2.754	0.006
SZ	Malmqvist 2019	SZ	-0.044	-0.355	0.275	-0.266	0.790
SZ	Dal Santo 2020	SZ	-0.221	-0.378	-0.052	-2.552	0.011
SZ			-0.142	-0.241	-0.040	-2.724	0.006
Overall			-0.036	-0.049	-0.023	-5.533	0.000

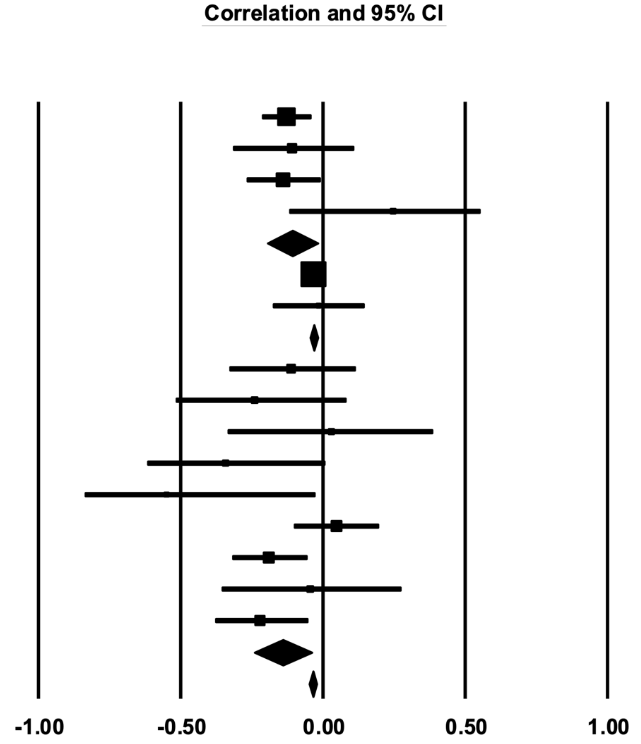

Supplementary Figure 9: Forest Plot for correlation studies between Pro Inflammatory Index (PII) and cognitive domain Working Memory for all studies, as well as diagnostic subgroups (Bipolar Disorder (BD), Major Depressive Disorder (MDD), and schizophrenia spectrum disorders (SZ))

Group by Subgroup within study	Study name	Diagnosis	Statistics for each study: Correlation	Lower limit	Upper limit	Z-Value	p-Value
BD	Lizano 2019	BD	-0.093	-0.232	0.050	-1.275	0.202
BD	Chakrabarty 2018	BD	-0.309	-0.539	-0.037	-2.213	0.027
BD	Millet 2019	BD	-0.214	-0.336	-0.085	-3.217	0.001
BD	Vanden Ameele 202[illegible]	BD	-0.052	-0.289	0.191	-0.416	0.677
BD	Strawbridge 2021	BD	-0.007	-0.303	0.291	-0.042	0.967
BD	Zazula 2021	BD	-0.236	-0.545	0.129	-1.273	0.203
BD			-0.153	-0.231	-0.074	-3.744	0.000
MDD	Ye 2018	MDD	0.110	-0.261	0.452	0.574	0.566
MDD			0.110	-0.261	0.452	0.574	0.566
SZ	Asevedo 2013	SZ	-0.400	-0.665	-0.046	-2.201	0.028
SZ	Frydecka 2015	SZ	0.066	-0.095	0.223	0.804	0.421
SZ	Hori 2016	SZ	-0.035	-0.196	0.128	-0.419	0.675
SZ	de Campos-Carli 20[illegible]	SZ	0.235	-0.083	0.509	1.457	0.145
SZ	Boozalis 2018	SZ	0.020	-0.297	0.333	0.120	0.904
SZ	Ergun 2018	SZ	0.170	-0.203	0.500	0.892	0.372
SZ	Kogan 2018	SZ	-0.055	-0.396	0.299	-0.296	0.767
SZ	Kudo 2018	SZ	0.080	-0.133	0.286	0.735	0.462
SZ	Lizano 2019	SZ	-0.048	-0.178	0.084	-0.713	0.476
SZ	Malmqvist 2019	SZ	0.026	-0.292	0.339	0.156	0.876
SZ	Ribeiro-Santos 2019	SZ	-0.024	-0.270	0.225	-0.186	0.852
SZ	Dal Santo 2020	SZ	-0.107	-0.273	0.065	-1.220	0.222
SZ	De Picker 2020	SZ	-0.120	-0.388	0.167	-0.818	0.413
SZ	Moustafa 2020	SZ	0.240	0.060	0.405	2.590	0.010
SZ	Klaus 2021	SZ	-0.500	-0.798	-0.006	-1.981	0.048
SZ	Zhang 2021	SZ	0.036	-0.193	0.261	0.306	0.760
SZ			0.002	-0.071	0.076	0.066	0.947
Overall			-0.065	-0.118	-0.011	-2.363	0.018

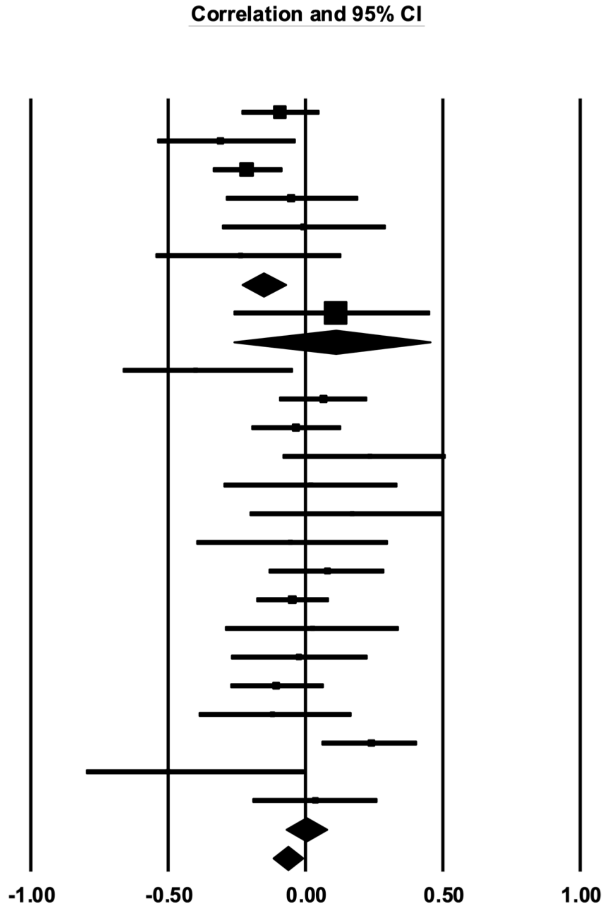

Supplementary Figure 10: Forest Plot for correlation studies between Anti Inflammatory Index (AII) and Global Cognition for all studies, as well as diagnostic subgroups (Bipolar Disorder (BD), Major Depressive Disorder (MDD), and schizophrenia spectrum disorders (SZ))

Study name	Diagnosis	Statistics for each study				
		Correlation	Lower limit	Upper limit	Z-Value	p-Value
Xiu 2016	SZ	0.217	0.045	0.376	2.465	0.014
Klaus 2021	SZ	-0.072	-0.548	0.439	-0.260	0.795
Belge 2020	MDD	-0.159	-0.393	0.095	-1.232	0.218
Strawbridge 2021	BD	0.143	-0.161	0.422	0.919	0.358
Frydecka 2015	SZ	0.053	-0.104	0.207	0.660	0.509
		0.067	-0.069	0.201	0.966	0.334

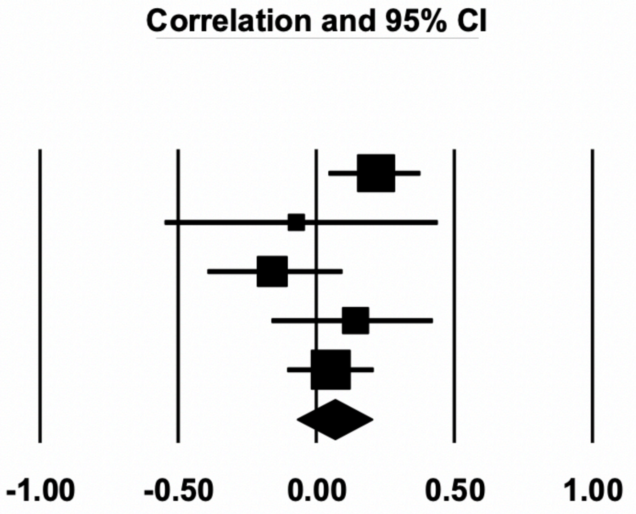

Supplementary Figure 11: Forest Plot for correlation studies between Anti Inflammatory Index (AII) and Cognitive Domain Language for all studies, as well as diagnostic subgroups (Bipolar Disorder (BD), Major Depressive Disorder (MDD), and schizophrenia spectrum disorders (SZ))

Group by Subgroup within study	Study name	Diagnosis	Correlation	Lower limit	Upper limit	Z-Value	p-Value
			Statistics for each study				
BD	Lizano 2019	BD	-0.065	-0.205	0.078	-0.890	0.373
BD	Dickerson 2013	BD	-0.204	-0.379	-0.014	-2.105	0.035
BD	Sanchez-autet 2018	BD	-0.329	-0.441	-0.207	-5.080	0.000
BD	Strawbridge 2021	BD	-0.031	-0.325	0.268	-0.199	0.843
BD			-0.177	-0.322	-0.024	-2.266	0.023
MDD	Bobinska 2016	MDD	-0.064	-0.269	0.146	-0.594	0.552
MDD	Tateishi 2020	MDD	0.469	-0.182	0.834	1.439	0.150
MDD			0.123	-0.383	0.572	0.459	0.646
SZ	Frydecka 2015	SZ	-0.106	-0.261	0.055	-1.294	0.196
SZ	Hori 2016	SZ	-0.020	-0.182	0.143	-0.239	0.811
SZ	de Campos-Carli 20[illegible]	SZ	0.321	0.011	0.575	2.024	0.043
SZ	Boozalis 2018	SZ	-0.300	-0.562	0.017	-1.857	0.063
SZ	Ergun 2018	SZ	-0.088	-0.434	0.281	-0.458	0.647
SZ	Kudo 2018	SZ	0.140	-0.073	0.341	1.292	0.196
SZ	Orhan 2018	SZ	-0.380	-0.674	0.018	-1.876	0.061
SZ	Fang 2019	SZ	-0.062	-0.209	0.088	-0.812	0.417
SZ	Fathian 2019	SZ	-0.350	-0.464	-0.225	-5.232	0.000
SZ	Weiser 2019	SZ	0.006	-0.094	0.106	0.121	0.903
SZ	Lizano 2019	SZ	0.018	-0.114	0.149	0.260	0.795
SZ	Borovcanin 2020	SZ	0.569	0.241	0.780	3.165	0.002
SZ	Moustafa 2020	SZ	0.233	0.052	0.399	2.512	0.012
SZ	Zhang 2021	SZ	-0.354	-0.538	-0.138	-3.140	0.002
SZ	King 2021	SZ	-0.079	-0.276	0.125	-0.759	0.448
SZ			-0.037	-0.145	0.071	-0.673	0.501
Overall			-0.078	-0.165	0.009	-1.750	0.080

Correlation and 95% CI

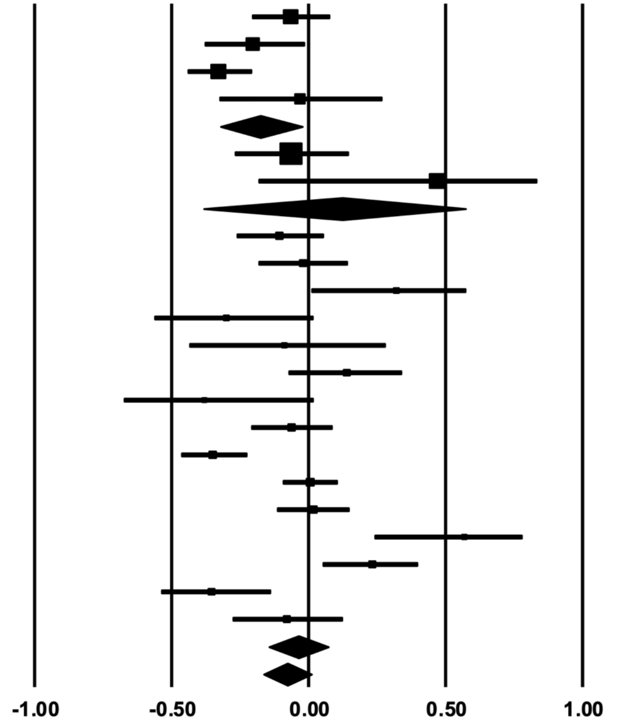

Supplementary Figure 12: Forest Plot for correlation studies between Anti Inflammatory Index (AII) and Cognitive Domain Processing Speed for all studies, as well as diagnostic subgroups (Bipolar Disorder (BD), Major Depressive Disorder (MDD), and schizophrenia spectrum disorders (SZ))

Study name	Diagnosis	Statistics for each study				
		Correlation	Lower limit	Upper limit	Z-Value	p-Value
Moustafa 2020	SZ	0.251	0.071	0.415	2.714	0.007
Goldsmith 2016	MDD	0.210	0.007	0.397	2.022	0.043
Belge 2020	MDD	-0.075	-0.319	0.178	-0.579	0.562
Strawbridge 2021	BD	0.011	-0.287	0.307	0.070	0.944
		0.125	-0.030	0.275	1.587	0.113

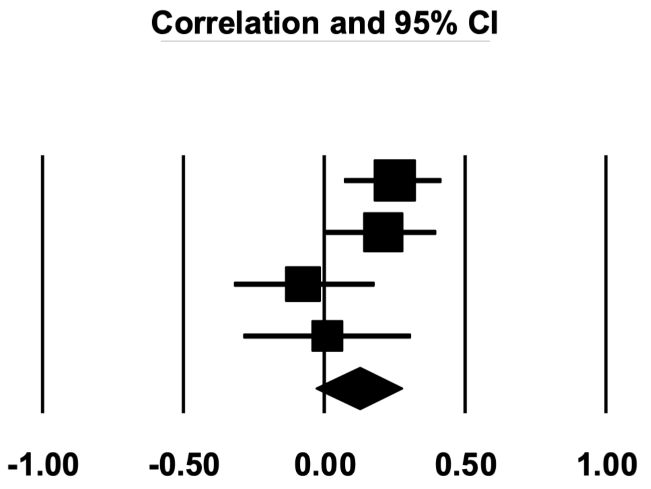

Supplementary Figure 13: Forest Plot for correlation studies between Anti Inflammatory Index (AII) and Cognitive Domain Reasoning for all studies, as well as diagnostic subgroups (Bipolar Disorder (BD), Major Depressive Disorder (MDD), and schizophrenia spectrum disorders (SZ))

Study name	Diagnosis	Correlation	Lower limit	Upper limit	Z-Value	p-Value
Klaus 2021	SZ	-0.243	-0.659	0.287	-0.894	0.371
Samagula 2017	MDD	-0.173	-0.497	0.193	-0.925	0.355
Strawbridge 2021	BD	-0.061	-0.352	0.240	-0.391	0.696
Frydecka 2015	SZ	0.230	0.077	0.373	2.916	0.004
		-0.002	-0.245	0.241	-0.019	0.985

Statistics for each study

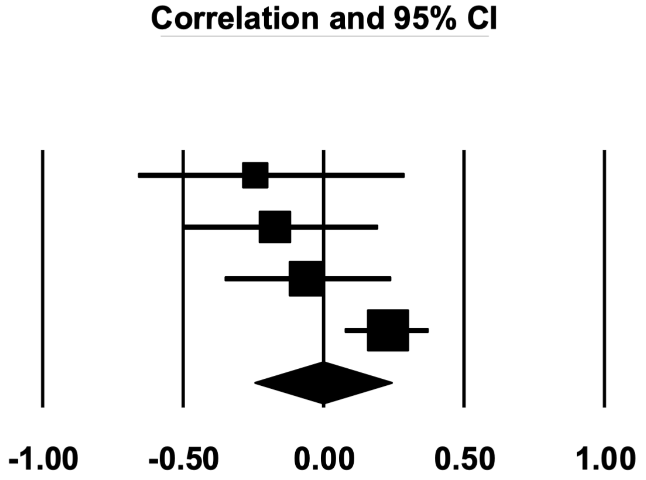

Supplementary Figure 14: Forest Plot for correlation studies between Anti Inflammatory Index (AII) and Cognitive Domain Verbal Memory for all studies, as well as diagnostic subgroups (Bipolar Disorder (BD), Major Depressive Disorder (MDD), and schizophrenia spectrum disorders (SZ))

Study name	Diagnosis	Statistics for each study				
		Correlation	Lower limit	Upper limit	Z-Value	p-Value
Belge 2020	MDD	-0.242	-0.464	0.008	-1.896	0.058
Strawbridge 2021	BD	0.118	-0.185	0.401	0.759	0.448
Frydecka 2015	SZ	-0.060	-0.214	0.097	-0.748	0.455
		-0.074	-0.240	0.097	-0.846	0.398

Correlation and 95% CI

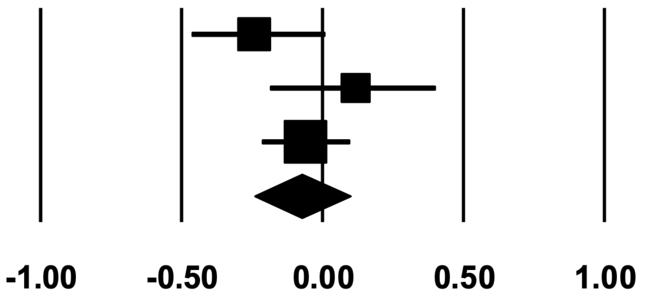

Supplementary Figure 15: Forest Plot for correlation studies between C Reactive Protein (CRP) and Global Cognition for all studies, as well as diagnostic subgroups (Bipolar Disorder (BD), Major Depressive Disorder (MDD), and schizophrenia spectrum disorders (SZ))

Group by Subgroup within study	Study name	Diagnosis	Statistics for each study: Correlation	Lower limit	Upper limit	Z-Value	p-Value
BD	Lizano 2019	BD	-0.220	-0.351	-0.080	-3.058	0.002
BD	Millet 2019	BD	-0.130	-0.257	0.002	-1.935	0.053
BD	Milton 2021	BD	0.000	-0.063	0.063	0.000	1.000
BD	Sanchez-autet 201[illegible]	BD	-0.270	-0.387	-0.144	-4.116	0.000
BD	Strawbridge 2021	BD	-0.019	-0.314	0.280	-0.120	0.905
BD	Tanaka 2017	BD	-0.001	-0.350	0.348	-0.005	0.996
BD	Tseng 2016	BD	-0.251	-0.594	0.170	-1.175	0.240
BD	Vanden Ameele 20[illegible]	BD	-0.075	-0.310	0.168	-0.601	0.548
BD			-0.126	-0.225	-0.024	-2.412	0.016
MDD	Belge 2020	MDD	-0.226	-0.451	0.025	-1.769	0.077
MDD	Milton 2021	MDD	0.009	-0.005	0.022	1.273	0.203
MDD	Qu 2019	MDD	-0.330	-0.491	-0.147	-3.445	0.001
MDD	Zhou 2021	MDD	0.058	-0.104	0.217	0.702	0.483
MDD			-0.103	-0.264	0.062	-1.223	0.221
SZ	Boozalis 2018	SZ	-0.030	-0.342	0.288	-0.180	0.857
SZ	Dal Santo 2020	SZ	-0.162	-0.323	0.010	-1.850	0.064
SZ	Fathian 2019	SZ	-0.370	-0.482	-0.246	-5.561	0.000
SZ	Frydecka 2015	SZ	0.050	-0.111	0.208	0.609	0.543
SZ	Johnsen 2016	SZ	-0.247	-0.406	-0.074	-2.774	0.006
SZ	Klaus 2021	SZ	-0.198	-0.632	0.330	-0.723	0.469
SZ	Lizano 2019	SZ	-0.080	-0.209	0.052	-1.189	0.234
SZ	Lizano 2020	SZ	-0.216	-0.369	-0.052	-2.569	0.010
SZ	Weiser 2019	SZ	0.008	-0.091	0.108	0.165	0.869
SZ			-0.139	-0.245	-0.030	-2.489	0.013
Overall			-0.127	-0.194	-0.059	-3.658	0.000

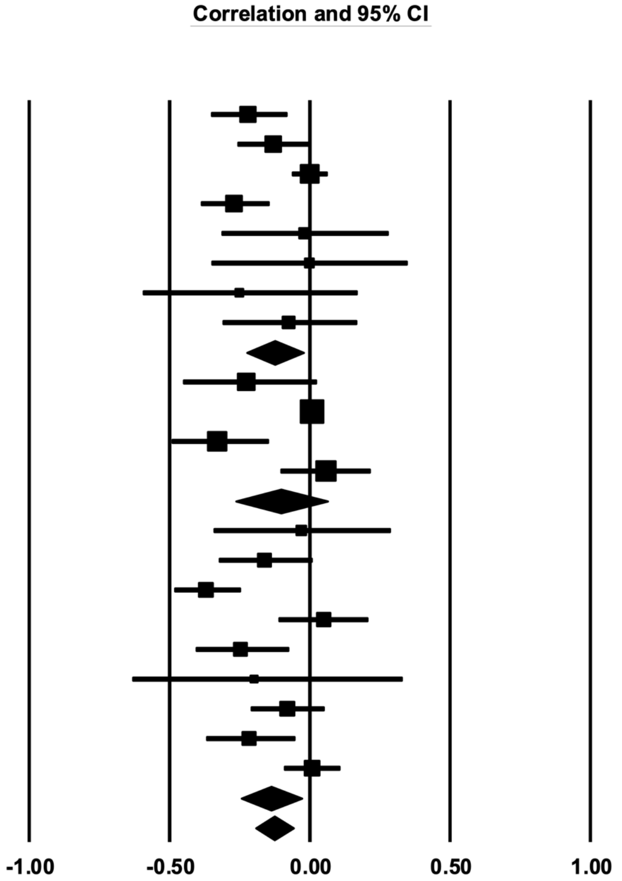

Supplementary Figure 16: Forest Plot for correlation studies between IFN-g and Global Cognition for all studies, as well as diagnostic subgroups (Bipolar Disorder (BD), Major Depressive Disorder (MDD), and schizophrenia spectrum disorders (SZ))

Study name	Diagnosis	Correlation	Lower limit	Upper limit	Z-Value	p-Value
Belge	MDD	0.139	-0.115	0.376	1.075	0.283
Wilson	SZ	-0.390	-0.763	0.177	-1.366	0.172
Klaus 2021	SZ	-0.295	-0.690	0.235	-1.096	0.273
Strawbridge 2021	BD	0.140	-0.164	0.419	0.902	0.367
Vanden Ameele 2021	BD	0.121	-0.123	0.351	0.973	0.331
		0.055	-0.112	0.219	0.647	0.517

(Statistics for each study)

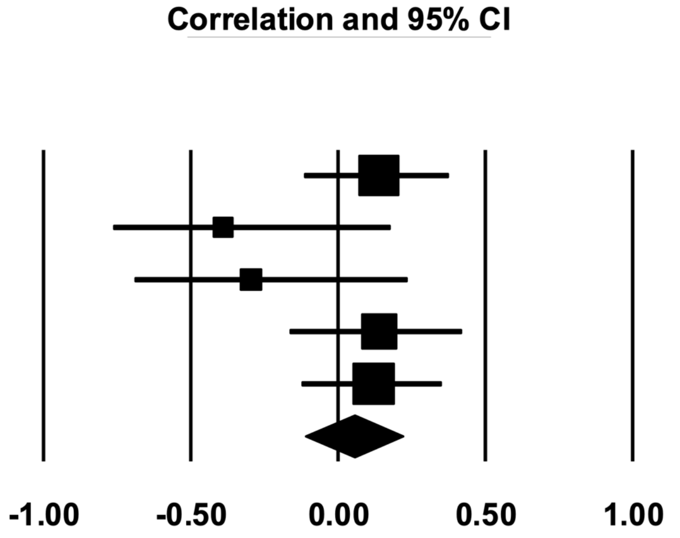

Supplementary Figure 17: Forest Plot for correlation studies between IL-1b and Global Cognition for all studies, as well as diagnostic subgroups (Bipolar Disorder (BD), Major Depressive Disorder (MDD), and schizophrenia spectrum disorders (SZ))

Group by Subgroup within study	Study name	Diagnosis	Statistics for each study				
			Correlation	Lower limit	Upper limit	Z-Value	p-Value
BD	Lizano 2019	BD	-0.100	-0.239	0.043	-1.372	0.170
BD			-0.100	-0.239	0.043	-1.372	0.170
MDD	Belge 2020	MDD	0.076	-0.177	0.320	0.585	0.559
MDD	Goldsmith 2016	MDD	-0.004	-0.208	0.200	-0.040	0.968
MDD	Qu 2019	MDD	-0.290	-0.457	-0.103	-3.001	0.003
MDD	Tateishi 2020	MDD	0.050	-0.567	0.631	0.142	0.887
MDD	Zhou 2021	MDD	0.078	-0.084	0.236	0.944	0.345
MDD			-0.035	-0.198	0.130	-0.416	0.677
SZ	De Picker 2020	SZ	-0.124	-0.392	0.163	-0.845	0.398
SZ	Kogan 2018	SZ	-0.186	-0.502	0.174	-1.013	0.311
SZ	Lizano 2019	SZ	-0.020	-0.151	0.112	-0.297	0.767
SZ	Zhang 2021	SZ	-0.296	-0.490	-0.074	-2.589	0.010
SZ			-0.131	-0.270	0.013	-1.789	0.074
Overall			-0.094	-0.179	-0.007	-2.122	0.034

Correlation and 95% CI

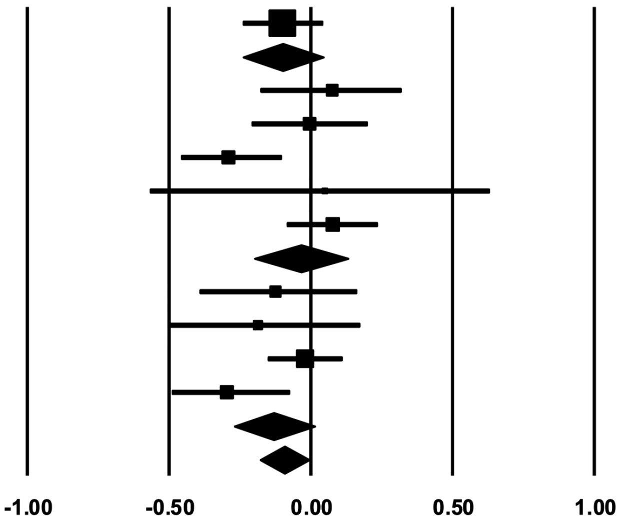

Supplementary Figure 18: Forest Plot for correlation studies between IL-6 and Global Cognition for all studies, as well as diagnostic subgroups (Bipolar Disorder (BD), Major Depressive Disorder (MDD), and schizophrenia spectrum disorders (SZ))

Group by Subgroup within study	Study name	Diagnosis	Statistics for each study: Correlation	Lower limit	Upper limit	Z-Value	p-Value
BD	Barbosa 2018	BD	-0.461	-0.750	-0.023	-2.056	0.040
BD	Lizano 2019	BD	-0.340	-0.460	-0.208	-4.842	0.000
BD	Mora 2018	BD	0.165	-0.051	0.366	1.499	0.134
BD	Strawbridge 2021	BD	-0.080	-0.368	0.223	-0.510	0.610
BD	vanden Ameele 2021	BD	-0.075	-0.310	0.168	-0.604	0.546
BD			-0.146	-0.365	0.089	-1.217	0.224
MDD	Ali 2018	MDD	-0.552	-0.737	-0.290	-3.779	0.000
MDD	Belge 2020	MDD	-0.261	-0.479	-0.012	-2.049	0.040
MDD	Elderkin-Thompson 2012	MDD	-0.250	-0.515	0.058	-1.595	0.111
MDD	Goldsmith 2016	MDD	0.049	-0.157	0.250	0.460	0.645
MDD	Peters 2019	MDD	-0.048	-0.354	0.268	-0.289	0.772
MDD	Qu 2019	MDD	-0.700	-0.787	-0.586	-8.716	0.000
MDD	Samagula 2017	MDD	0.220	-0.146	0.533	1.183	0.237
MDD	Tateishi 2020	MDD	0.007	-0.595	0.604	0.020	0.984
MDD	Ye 2018	MDD	-0.083	-0.430	0.286	-0.432	0.666
MDD	Zhou 2021	MDD	-0.046	-0.206	0.115	-0.561	0.575
MDD			-0.203	-0.416	0.030	-1.708	0.088
SZ	De Picker 2020	SZ	-0.181	-0.440	0.106	-1.241	0.215
SZ	Ergun 2018	SZ	0.041	-0.324	0.395	0.213	0.831
SZ	Fang 2019	SZ	-0.059	-0.206	0.091	-0.772	0.440
SZ	Frydecka 2015	SZ	-0.167	-0.318	-0.007	-2.046	0.041
SZ	Hori 2016	SZ	0.049	-0.115	0.209	0.580	0.562
SZ	King 2021	SZ	-0.158	-0.340	0.036	-1.601	0.109
SZ	Klaus 2021	SZ	-0.469	-0.783	0.035	-1.834	0.067
SZ	Kogan 2018	SZ	-0.301	-0.588	0.053	-1.673	0.094
SZ	Lizano 2019	SZ	-0.110	-0.238	0.022	-1.638	0.101
SZ	Lizano 2020	SZ	-0.296	-0.440	-0.137	-3.571	0.000
SZ	Moustafa 2020	SZ	0.175	-0.008	0.347	1.871	0.061
SZ	Ribeiro-Santos 2019	SZ	-0.395	-0.585	-0.163	-3.236	0.001
SZ			-0.131	-0.227	-0.034	-2.635	0.008
Overall			-0.143	-0.224	-0.059	-3.320	0.001

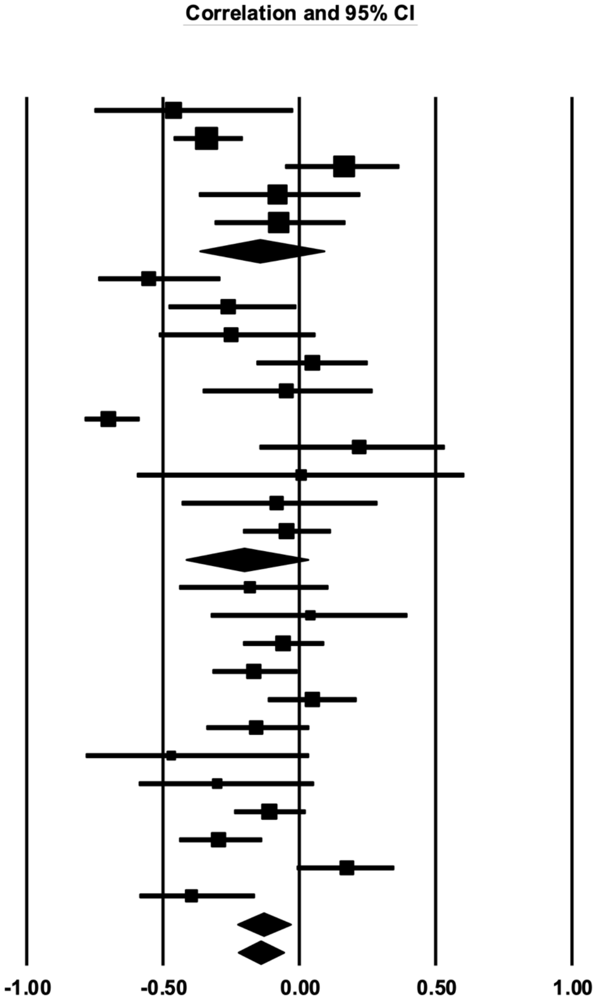

Supplementary Figure 19: Forest Plot for correlation studies between TNF-a and Global Cognition for all studies, as well as diagnostic subgroups (Bipolar Disorder (BD), Major Depressive Disorder (MDD), and schizophrenia spectrum disorders (SZ))

Group by Subgroup within study	Study name	Diagnosis	Statistics for each study				
			Correlation	Lower limit	Upper limit	Z-Value	p-Value
BD	Chakrabarty 2018	BD	-0.333	-0.557	-0.063	-2.399	0.016
BD	Doganavsargil-Baysal 2013	BD	-0.128	-0.383	0.145	-0.919	0.358
BD	Lizano 2019	BD	0.150	0.008	0.286	2.067	0.039
BD	Millet 2020	BD	-0.006	-0.138	0.127	-0.088	0.930
BD	Mora 2018	BD	0.038	-0.178	0.250	0.342	0.732
BD	Strawbridge 2021	BD	0.025	-0.274	0.319	0.157	0.875
BD	vanden Ameele 2021	BD	-0.057	-0.293	0.186	-0.454	0.650
BD			-0.018	-0.127	0.091	-0.329	0.742
MDD	Belge 2020	MDD	-0.127	-0.365	0.127	-0.982	0.326
MDD	Bobinska 2016	MDD	-0.093	-0.296	0.118	-0.865	0.387
MDD	Goldsmith 2016	MDD	-0.028	-0.231	0.176	-0.269	0.788
MDD	Peters 2019	MDD	0.120	-0.199	0.416	0.733	0.463
MDD	Tateishi 2020	MDD	0.083	-0.544	0.650	0.235	0.814
MDD	Zhou 2021	MDD	0.001	-0.160	0.162	0.009	0.993
MDD			-0.030	-0.125	0.065	-0.624	0.533
SZ	Amin 2020	SZ	-0.011	-0.288	0.268	-0.075	0.940
SZ	De Picker 2020	SZ	-0.059	-0.335	0.226	-0.401	0.689
SZ	Ergun 2018	SZ	0.022	-0.341	0.379	0.114	0.909
SZ	Hori 2016	SZ	-0.046	-0.207	0.117	-0.550	0.582
SZ	Klaus 2021	SZ	-0.610	-0.849	-0.164	-2.556	0.011
SZ	Kogan 2018	SZ	-0.303	-0.589	0.051	-1.685	0.092
SZ	Lizano 2019	SZ	-0.060	-0.190	0.072	-0.891	0.373
SZ	Ribeiro-Santos 2019	SZ	0.100	-0.152	0.339	0.777	0.437
SZ	Zazula 2021	SZ	-0.033	-0.382	0.326	-0.172	0.863
SZ	Zhang 2021	SZ	-0.027	-0.252	0.201	-0.229	0.819
SZ			-0.055	-0.131	0.022	-1.403	0.160
Overall			-0.039	-0.091	0.013	-1.459	0.144

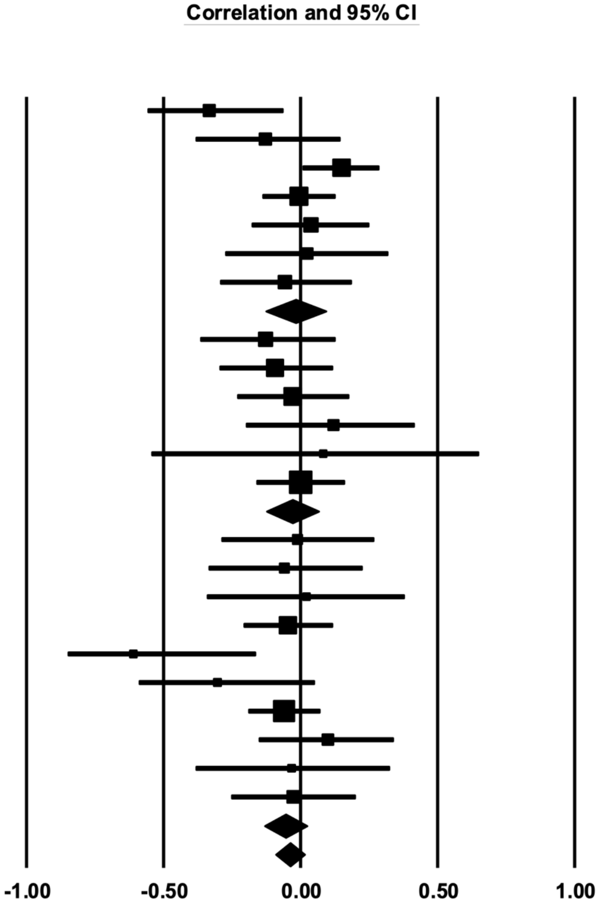

Supplementary Figure 20: Funnel Plot Correlation studies of Pro Inflammatory Index (PII) with Global Cognition

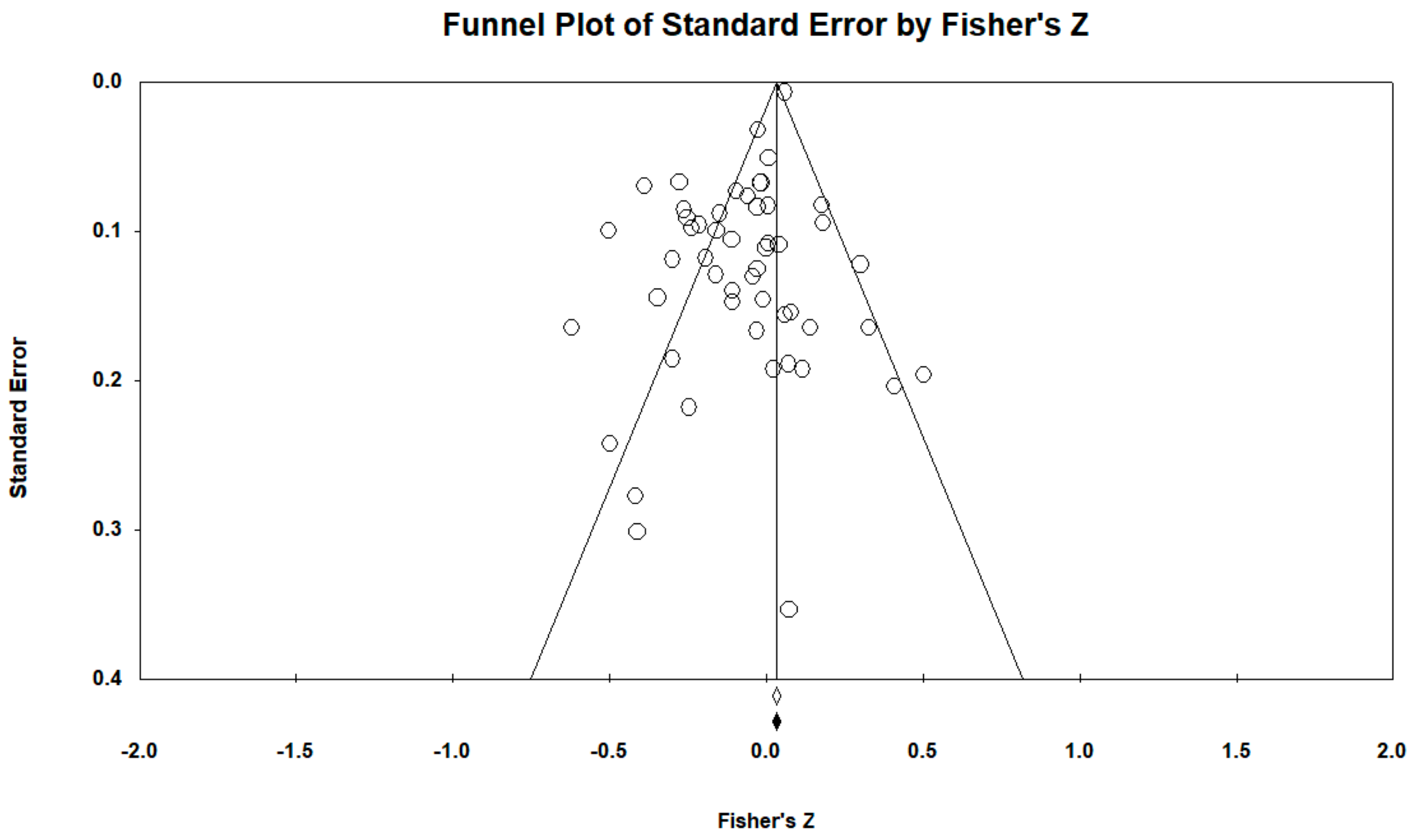

Supplementary Figure 21: Funnel Plot Correlation Studies CRP with Global Cognition

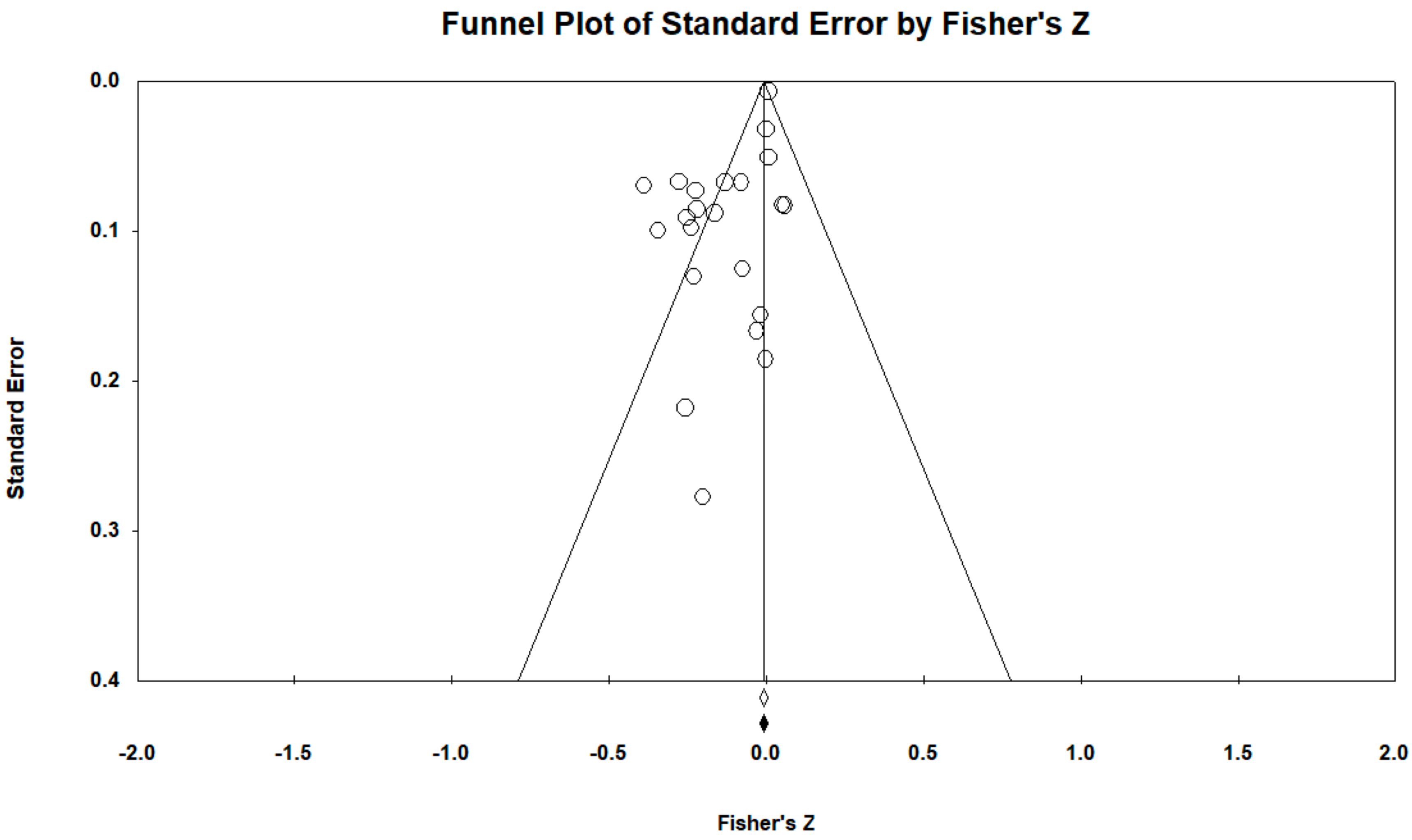

Supplementary Figure 22: Funnel Plot Correlation Studies IL-6 with Global Cognition

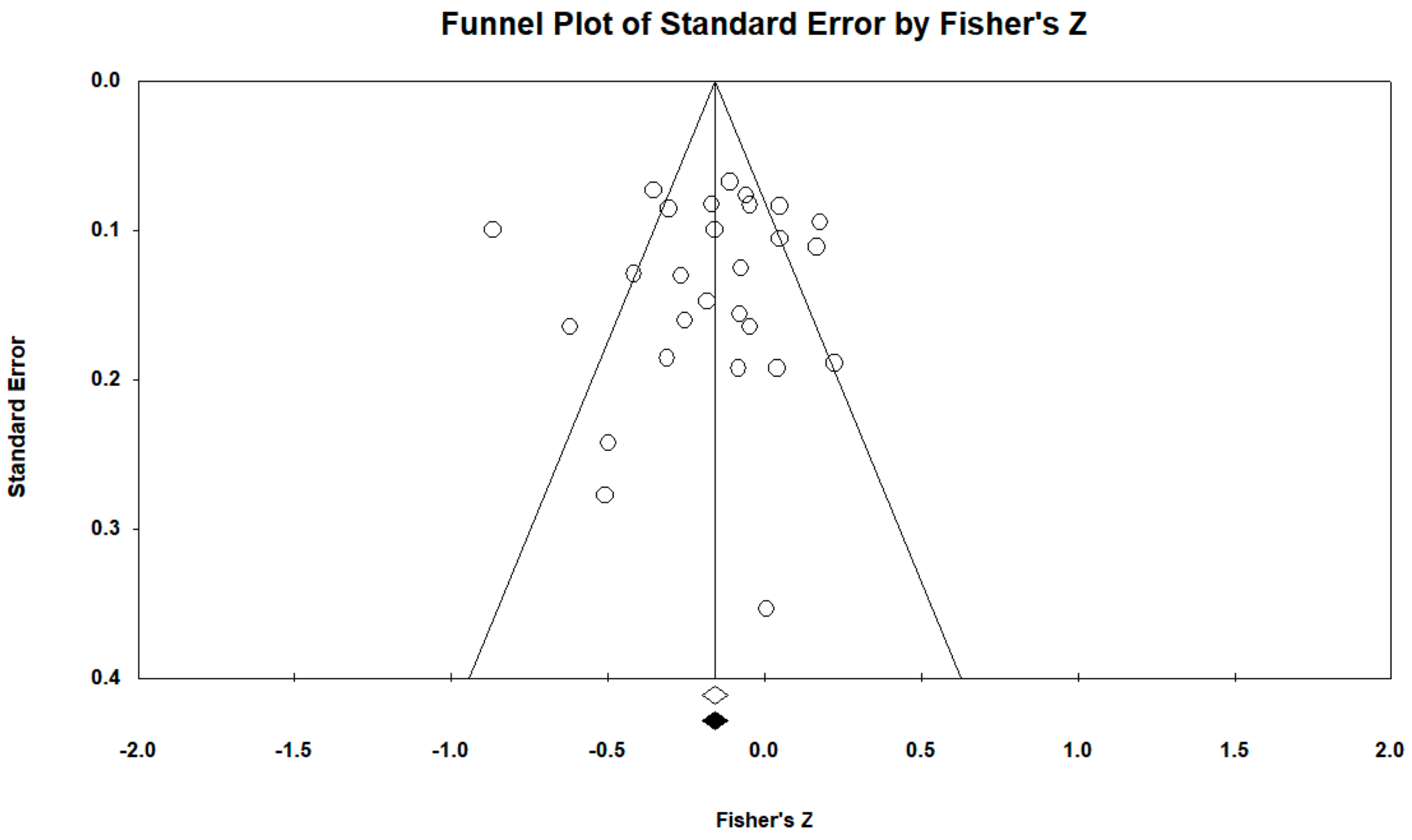

Supplementary document: Protocol (12/9/2021) + Update to protocol 4/1/2022 (PROSPERO) + Final Protocol (version 4/1/2022)

Supplementary document: Protocol : The relationship between immune and cognitive dysfunction in mood and psychotic disorder: a systematic review and a meta-analysis
Submitted PROSPERO: 12/9/2021

The current meta-analysis aims to evaluate the presence of an association between immune-related aberrances and neuropsychological deficits in mood and psychotic disorders. The impact of reporting and publication bias will be evaluated.

Review Questions

1) Are neuropsychological deficits associated to centrally assessed immune-related biomarkers in mood and psychotic disorder?
2) Are neuropsychological deficits associated to peripherally assessed immune-related biomarkers in mood and psychotic disorders?
3) If present, are these associations different in mood versus psychotic disorders?
4) Are these associations moderated by publication year, age, duration of illness, gender, smoking status or BMI?

Studies included in the review:

- Cross-sectional or longitudinal studies in peer reviewed journals
- Studies investigating a patient group with either psychotic or mood disorder, i.e. Primary diagnosis of Schizophrenia, schizophreniform disorder, Schizoaffective disorder, Psychotic disorder NOS, Brief Psychotic Disorder, Major Depressive disorder, Bipolar Disorder, Dysthymia
- Inclusion of at least 1 neuropsychological measure (NP)
- Inclusion of at least 1 immune-related marker (IM)

Exclusion criteria

- Case reports, case series
- Abstracts, conference proceedings
- Systematic review, narrative review
- Opinion paper
- Meta-analysis

Search strategy

platform	Search strategy
Pubmed	(schizophreni* OR schizoaffective OR psychotic OR psychosis OR MDD OR “depressive disorder” OR “major depression” OR manic OR mania OR bipolar OR “mood disorder” OR “affective disorder” OR dysthym*) AND (immun* OR chemokine* OR cytokine* OR interleukin* OR CRP OR C-reactive OR IL-1b OR IL-1β OR IL-1ra OR IL-2 OR IL-4 OR IL-6 OR IL-8 OR IL-10 OR IL-12 OR “tumor necrosis factor” OR TNF OR IFN OR IFN-γ OR Interferon* OR kynuren* OR quinolinic OR xanthurenic OR picolinic OR anthranilic OR HLA OR lymphocyt* OR monocyt* OR Leukocyt* OR neutrophil* OR basophil* or eosinophil*) AND (cogniti* OR attenti* OR vigilan* OR “executive functioning” OR reasoning OR “problem solving” OR memory OR learning OR “processing speed”) NOT (review [Publication type])
Web of Science	(ALL=(schizophreni*) OR ALL=(schizoaffective) OR ALL=(psychotic) OR ALL=(psychosis) OR ALL=(MDD) OR ALL=(depressive disorder) OR ALL=(major depression) OR ALL=(manic) OR ALL=(mania) OR ALL=(bipolar) OR ALL=(mood disorder) OR ALL=(affective disorder) OR ALL=(dysthym*)) AND (ALL=(immun*) OR ALL=(chemokine*) OR ALL=(cytokine*) OR ALL=(interleukin*) OR ALL=(CRP) OR ALL=(C-reactive) OR ALL=(IL-1b) OR ALL=(IL-1β) OR ALL=(IL-1ra) OR ALL=(IL-2) OR ALL=(IL-4) OR ALL=(IL-6) OR ALL=(IL-8) OR ALL=(IL-10) OR ALL=(IL-12) OR ALL=(tumor necrosis factor) OR ALL=(TNF) OR ALL=(IFN) OR ALL=(IFN-γ) OR ALL=(Interferon*) OR ALL=(kynuren*) OR ALL=(quinolinic) OR ALL=(xanthurenic) OR ALL=(picolinic) OR ALL=(anthranilic) OR ALL=(HLA) OR ALL=(lymphocyt*) OR ALL=(monocyt*) OR ALL=(leukocyt*) OR ALL=(neutrophil*) OR ALL=(basophil*) OR ALL=(eosinophil*)) AND (ALL=(cognitive*) OR ALL=(attenti*) OR ALL=(vigilan*) OR ALL=(executive functioning) OR ALL=(reasoning) OR ALL=(problem solving) OR ALL=(memory) OR ALL=(learning) OR ALL=(processing speed))
Psycinfo	**Any Field**: schizophrenia *OR* **Any Field**: schizophreniform *OR* **Any Field**: psychotic *OR* **Any Field**: psychosis *OR* **Any Field**: MDD *OR* **Any Field**: depressive disorder *OR* **Any Field**: major depression *OR* **Any Field**: manic *OR* **Any Field**: mania *OR* **Any Field**: bipolar *OR* **Any Field**: mood disorder *OR* **Any Field**: affective disorder *OR* **Any Field**: dysthymia *AND* **Any Field**: Immune OR cytokine OR chemokine OR interleukin OR CRP OR IL-1b OR IL-1β OR IL-1ra OR IL-2 OR IL-4 OR IL-6 OR IL-8 OR IL-10 OR IL-12 OR tumor necrosis factor OR TNF OR IFN OR IFN-γ OR Interferon OR kynurenine OR kynurenic OR quinolinic OR xanthurenic OR picolinic OR anthranilic OR HLA OR lymphocyte OR monocyte OR Leukocyte OR neutrophil OR basophil or eosinophil *AND* **Any Field**: cognition *OR* **Any Field**: cognitive *OR* **Any Field**: attention *OR* **Any Field**: vigilance *OR* **Any Field**: executive function *OR* **Any Field**: executive functioning *OR* **Any Field**: reasoning *OR* **Any Field**: problem solving *OR* **Any Field**: memory *OR* **Any Field**: learning *OR* **Any Field**: processing speed *AND* **Peer-Reviewed Journals only**

Main outcome measure:

A correlation value (pearson's r or rho value) or regression coefficient (B value) reporting on the association between neuropsychological measure(s) (NP) and immune-related marker(s) (IM)

Data extraction:

Two reviewers (MM and CO) will independently evaluate the search results. Title and abstract of all search hits will be evaluated based on inclusion and exclusion criteria. Relevant or potentially relevant papers will be extracted:
- author, publication year, diagnosis, sample size, mean age, mean DOI, gender ratio, %smoking, mean BMI, name of cognitive domain, name of cognitive test, immune biomarker, tissue (serum, plasma, CSF, tracer, cells), bioanalytical method - correlation (pearson's r or rho value) or regression coefficient (B value) between neuropsychological measure(s) and immune-related biomarker(s), p-value.
- Number of neuropsychological tests included in the study
- number of immune biomarkers included in the study
- number of reported correlations

Data will be recorded in excel sheet.

Investigated cognitive domains and cognitive tests included

Domain	Tests
Verbal learning and memory	List learning tasks (RBANS-memory, BACS, CVLT, RaVLT,…)
Visual learning and memory	RBANS-visuospatial; pairs matching test;
Working memory	LNS, Letter sequencing, Digit backward, spatial span test, Digit sequencing test, BACS-WM, MCCB-WM, delayed match to sample test,
Attention and vigilance	RBANS-attention, MCCB-attention, BACS-attention, CANTAB-attention, CPT, Digit span, simple reaction time
Processing speed	Digit Symbol Coding tests, TMTB
Reasoning and problem solving	WCST, Tower of London, CANTAB-set shift, Stroop test, Mazes, BACS-EF, abstraction test, matrix reasoning, MCCB reasoning,
Language	RBANS-language, Verbal Fluency tests, COWAT, naming test

When in a single study more than one IM is correlated to a specific cognitive domain on a composite score will be calculated (based on FIsher's z transformation of the r values), resulting in a single r score reflecting the association of the merged correlational values between the cognitive domain and the pro-inflammatory and anti-inflammatory markers.

Authors of the papers with missing results will be contacted. In case of non-response, a reminder will be sent.

<u>Risk of Bias Assessment:</u>
Biocross will be used to assess the quality of the studies

<u>Strategy for meta-analysis</u>

- Comprehensive Meta Analysis (CMA) will be used for the meta-analysis.
- Correlation and 95% CI as well as Z-value and p value will be reported per study, as well as for the overall summary of the random model
- Analyses will be performed for:
  - Psychotic and mood disorders combined, as well as for three diagnostic groups separately (SZ, BD, MDD)
  - For pro-inflammatory markers combined
  - For anti-inflammatory markers combined
  - For biomarkers that have been investigated in at least three different, independent studies in the same diagnosis (e.g. CRP, IL-6, IL-1b) for the same cognitive domain, separate meta-analyses will be performed

Update to protocol 4/1/2022

Review Question
Given that MDD and BD should be evaluated as separate diagnoses, rather than a group of 'mood disorders', we chose to separately analyze these diagnostic groups, rather than lumping them together

Research question 3 was adapted: "If present, are these associations different in mood versus psychotic disorders?"

was adjusted into

"Are those associations different between Schizophrenia (SZ), Major Depressive Disorder (MDD) and Bipolar Disorder (BD)?"

Main outcome(s)

The following analyses were already described in the analyses plan of the original PROSPERO registration, but we chose to clarify how the pro-inflammatory index (PII) and Anti-inflammatory Index (AII) were calculated. Therefore we added the following passage in the updated registration.

The following passage needs to be added:
"The primary Immunomarker (IM) outcome variables will be two composite inflammatory indices
- Pro-inflammatory Index, PII
- Anti-Inflammatory Index, AII

If only a single IM is included in the study, that IM x Neuropsychological measure correlation coefficient will be used for the PII/AII associations. If more IM are included, PII and AII will be calculated for each cognitive domain, averaging out the associations between that specific cognitive domain on one hand and the available immunomarker-based correlations on the other (based on Fisher's z transformation of the r values)."

"Primary outcome of the study is PII/AII x composite score for all combined diagnoses.
PII/AII x individual cognitive domains for combined diagnosis and the separate diagnoses (SZ, MDD, BD) will also be calculated and reported"

Additional outcomes
The following analyses were performed as secondary outcome analyses

“Secondary IM outcome variables are individual IM when >3 studies are available.

In case of a significant association, exploratory subgroup analyses will be performed to define which of the diagnoses contributes to the statistically significant association.”

Risk of bias (quality) assessment

Because the majority of the studies were cross-sectional studies, GRADE could not be used as a tool for Quality Assessment. Therefore, we chose the more appropriate tool BIOCROSS.

“Quality assessment of eligible papers was performed with the BIOCROSS evaluation tool, which is specifically developed for biomarker-based cross-sectional studies.”

Analysis of subgroups or subsets
In line with the adjustments made in RQ3

Three patient subgroups will be analyzed:
- all diagnoses combined
- psychotic disorders and mood disorders separately

Was changed into

Three patient subgroups will be analyzed:
- all diagnoses combined
- Schizophrenia, Major Depressive Disorder and Bipolar Disorder separately

Supplementary document: Final Protocol : The relationship between immune and cognitive dysfunction in mood and psychotic disorder: a systematic review and a meta-analysis
PROSPERO 2021 CRD42021278371

Version 4/1/2022

The current meta-analysis aims to evaluate the presence of an association between immune-related aberrances and neuropsychological deficits in mood and psychotic disorders. The impact of reporting and publication bias will be evaluated.

<u>Review Questions</u>

5) Are neuropsychological deficits associated to centrally assessed immune-related biomarkers in mood and psychotic disorder?
6) Are neuropsychological deficits associated to peripherally assessed immune-related biomarkers in mood and psychotic disorders?
7) Are those associations different between Schizophrenia (SZ), Major Depressive Disorder (MDD) and Bipolar Disorder (BD)?"
8) Are these associations moderated by publication year, age, duration of illness, gender, smoking status or BMI?

<u>Studies included in the review</u>:

- Cross-sectional or longitudinal studies in peer reviewed journals
- Studies investigating a patient group with either psychotic or mood disorder, i.e. Primary diagnosis of Schizophrenia, schizophreniform disorder, Schizoaffective disorder, Psychotic disorder NOS, Brief Psychotic Disorder, Major Depressive disorder, Bipolar Disorder, Dysthymia
- Inclusion of at least 1 neuropsychological measure (NP)
- Inclusion of at least 1 immune-related marker (IM)

<u>Exclusion criteria</u>

- Case reports, case series
- Abstracts, conference proceedings
- Systematic review, narrative review
- Opinion paper
- Meta-analysis

Search strategy

platform	Search strategy
Pubmed	(schizophreni* OR schizoaffective OR psychotic OR psychosis OR MDD OR “depressive disorder” OR “major depression” OR manic OR mania OR bipolar OR “mood disorder” OR “affective disorder” OR dysthym*) AND (immun* OR chemokine* OR cytokine* OR interleukin* OR CRP OR C-reactive OR IL-1b OR IL-1β OR IL-1ra OR IL-2 OR IL-4 OR IL-6 OR IL-8 OR IL-10 OR IL-12 OR “tumor necrosis factor” OR TNF OR IFN OR IFN-γ OR Interferon* OR kynuren* OR quinolinic OR xanthurenic OR picolinic OR anthranilic OR HLA OR lymphocyt* OR monocyt* OR Leukocyt* OR neutrophil* OR basophil* or eosinophil*) AND (cogniti* OR attenti* OR vigilan* OR “executive functioning” OR reasoning OR “problem solving” OR memory OR learning OR “processing speed”) NOT (review [Publication type])
Web of Science	(ALL=(schizophreni*) OR ALL=(schizoaffective) OR ALL=(psychotic) OR ALL=(psychosis) OR ALL=(MDD) OR ALL=(depressive disorder) OR ALL=(major depression) OR ALL=(manic) OR ALL=(mania) OR ALL=(bipolar) OR ALL=(mood disorder) OR ALL=(affective disorder) OR ALL=(dysthym*)) AND (ALL=(immun*) OR ALL=(chemokine*) OR ALL=(cytokine*) OR ALL=(interleukin*) OR ALL=(CRP) OR ALL=(C-reactive) OR ALL=(IL-1b) OR ALL=(IL-1β) OR ALL=(IL-1ra) OR ALL=(IL-2) OR ALL=(IL-4) OR ALL=(IL-6) OR ALL=(IL-8) OR ALL=(IL-10) OR ALL=(IL-12) OR ALL=(tumor necrosis factor) OR ALL=(TNF) OR ALL=(IFN) OR ALL=(IFN-γ) OR ALL=(Interferon*) OR ALL=(kynuren*) OR ALL=(quinolinic) OR ALL=(xanthurenic) OR ALL=(picolinic) OR ALL=(anthranilic) OR ALL=(HLA) OR ALL=(lymphocyt*) OR ALL=(monocyt*) OR ALL=(leukocyt*) OR ALL=(neutrophil*) OR ALL=(basophil*) OR ALL=(eosinophil*)) AND (ALL=(cognitive*) OR ALL=(attenti*) OR ALL=(vigilan*) OR ALL=(executive functioning) OR ALL=(reasoning) OR ALL=(problem solving) OR ALL=(memory) OR ALL=(learning) OR ALL=(processing speed))
Psycinfo	**Any Field**: schizophrenia *OR* **Any Field**: schizophreniform *OR* **Any Field**: psychotic *OR* **Any Field**: psychosis *OR* **Any Field**: MDD *OR* **Any Field**: depressive disorder *OR* **Any Field**: major depression *OR* **Any Field**: manic *OR* **Any Field**: mania *OR* **Any Field**: bipolar *OR* **Any Field**: mood disorder *OR* **Any Field**: affective disorder *OR* **Any Field**: dysthymia *AND* **Any Field**: Immune OR cytokine OR chemokine OR interleukin OR CRP OR IL-1b OR IL-1β OR IL-1ra OR IL-2 OR IL-4 OR IL-6 OR IL-8 OR IL-10 OR IL-12 OR tumor necrosis factor OR TNF OR IFN OR IFN-γ OR Interferon OR kynurenine OR kynurenic OR quinolinic OR xanthurenic OR picolinic OR anthranilic OR HLA OR lymphocyte OR monocyte OR Leukocyte OR neutrophil OR basophil or eosinophil *AND* **Any Field**: cognition *OR* **Any Field**: cognitive *OR* **Any Field**: attention *OR* **Any Field**: vigilance *OR* **Any Field**: executive function *OR* **Any Field**: executive functioning *OR* **Any Field**: reasoning *OR* **Any Field**: problem solving *OR* **Any Field**: memory *OR* **Any Field**: learning *OR* **Any Field**: processing speed *AND* **Peer-Reviewed Journals only**

Main outcome measure:

Correlation value (pearson's r or rho value) or regression coefficient (B value) reporting on the association between neuropsychological measure(s) (NP) and immune-related marker(s) (IM) will be included in the study.

The primary Immunomarker (IM) outcome variables will be two composite inflammatory indices

- Pro-inflammatory Index, PII
- Anti-Inflammatory Index, AII

If only a single IM is included in the study, that IM x Neuropsychological measure correlation coefficient will be used for the PII/AII associations. If more IM are included, PII and AII will be calculated for each cognitive domain, averaging out the associations between that specific cognitive domain on one hand and the available immunomarker-based correlations on the other (based on Fisher's z transformation of the r values)."

Primary outcome of the study is PII/AII x composite score for all combined diagnoses.
PII/AII x individual cognitive domains for combined diagnosis and the separate diagnoses (SZ, MDD, BD) will also be calculated and reported.

Additional outcomes

Secondary IM outcome variables are individual IM when >3 studies are available.

In case of a significant association, exploratory subgroup analyses will be performed to define which of the diagnoses contributes to the statistically significant association.

Data extraction:

Two reviewers (MM and CO) will independently evaluate the search results. Title and abstract of all search hits will be evaluated based on inclusion and exclusion criteria. Relevant or potentially relevant papers will be extracted:
- author, publication year, diagnosis, sample size, mean age, mean DOI, gender ratio, %smoking, mean BMI, name of cognitive domain, name of cognitive test, immune biomarker, tissue (serum, plasma, CSF, tracer, cells), bioanalytical method - correlation (pearson's r or rho value) or regression coefficient (B value) between neuropsychological measure(s) and immune-related biomarker(s), p-value.
- Number of neuropsychological tests included in the study
- number of immune biomarkers included in the study
- number of reported correlations

Data will be recorded in excel sheet.

Investigated cognitive domains and cognitive tests included

Domain	Tests
Verbal learning and memory	List learning tasks (RBANS-memory, BACS, CVLT, RaVLT,…)
Visual learning and memory	RBANS-visuospatial; pairs matching test;
Working memory	LNS, Letter sequencing, Digit backward, spatial span test, Digit sequencing test, BACS-WM, MCCB-WM, delayed match to sample test,
Attention and vigilance	RBANS-attention, MCCB-attention, BACS-attention, CANTAB-attention, CPT, Digit span, simple reaction time
Processing speed	Digit Symbol Coding tests, TMTB
Reasoning and problem solving	WCST, Tower of London, CANTAB-set shift, Stroop test, Mazes, BACS-EF, abstraction test, matrix reasoning, MCCB reasoning,
Language	RBANS-language, Verbal Fluency tests, COWAT, naming test

Authors of the papers with missing results will be contacted. In case of non-response, a reminder will be sent.

Risk of Bias Assessment:

Quality assessment of eligible papers was performed with the BIOCROSS evaluation tool, which is specifically developed for biomarker-based cross-sectional studies

Strategy for meta-analysis

- Comprehensive Meta Analysis (CMA) will be used for the meta-analysis.
- Correlation and 95% CI as well as Z-value and p value will be reported per study, as well as for the overall summary of the random model
- Analyses will be performed for:

- Composite cognitive score as well as individual cognitive domains
- Psychotic and mood disorders combined, as well as for three diagnostic groups separately (SZ, BD, MDD)
- For pro-inflammatory markers combined
- For anti-inflammatory markers combined
- For biomarkers that have been investigated in at least three different, independent studies in the same diagnosis (e.g. CRP, IL-6, IL-1b) for the same cognitive domain, separate meta-analyses will be performed
